# Cascade alkylarylation of substituted *N*-allylbenzamides for the construction of dihydroisoquinolin-1(2*H*)-ones and isoquinoline-1,3(2*H*,4*H*)-diones

**DOI:** 10.3762/bjoc.12.32

**Published:** 2016-02-17

**Authors:** Ping Qian, Bingnan Du, Wei Jiao, Haibo Mei, Jianlin Han, Yi Pan

**Affiliations:** 1 School of Chemistry and Chemical Engineering, State Key Laboratory of Coordination Chemistry, Nanjing University, Nanjing, 210093, China; 2 Institute for Chemistry & BioMedical Sciences, Nanjing University, Nanjing, 210093, China

**Keywords:** C(sp^3^)–H bond functionalization, cyclization, dihydroisoquinolin-1(2*H*)-one, *N*-allylbenzamides, oxidation

## Abstract

An oxidative reaction for the synthesis of 4-alkyl-substituted dihydroisoquinolin-1(2*H*)-ones with *N*-allylbenzamide derivatives as starting materials has been developed. The radical alkylarylation reaction proceeds through a sequence of alkylation and intramolecular cyclization. The substituent on the C–C double bond was found to play a key role for the progress of the reaction to give the expected products with good chemical yields. Additionally, *N*-methacryloylbenzamides were also suitable substrates for the current reaction and provided the alkyl-substituted isoquinoline-1,3(2*H*,4*H*)-diones in good yield.

## Introduction

The direct and selective functionalization of an unactivated sp^3^ C–H bond, which belongs to an effective strategic approach in green and sustainable chemistry, has attracted significant research attention [1–4]. This fascinating approach has obvious advantages in functional group transformation and construction of biological heterocycles, due to its high efficiency and waste reduction [5–10]. The pioneering works were focused on the cross-dehydrogenative coupling (CDC) reactions of alkanes, which were reported by Li and other groups [11–15]. Recently, several types of reactions with alkanes as substrates have been developed, such as the Minisci reaction with heteroarenes [16–17], radical addition to unsaturated bonds [18–19], decarboxylative alkenylation of cycloalkanes with aryl vinylic carboxylic acids [20–21], trifluoromethylthiolation [22], thiolation [23–24], alkenylation [25–26], dehydrogenation−olefination and esterification [27–28], radical addition/1,2-aryl migration [29], cascade alkylation-initiated cyclization [30–31] and other radical reactions [32–34]. Due to their low polarity and high bond-dissociation energy, the functionalization of unactivated sp^3^ C–H bonds in simple alkanes remains as a challenging task.

**Scheme 1:**
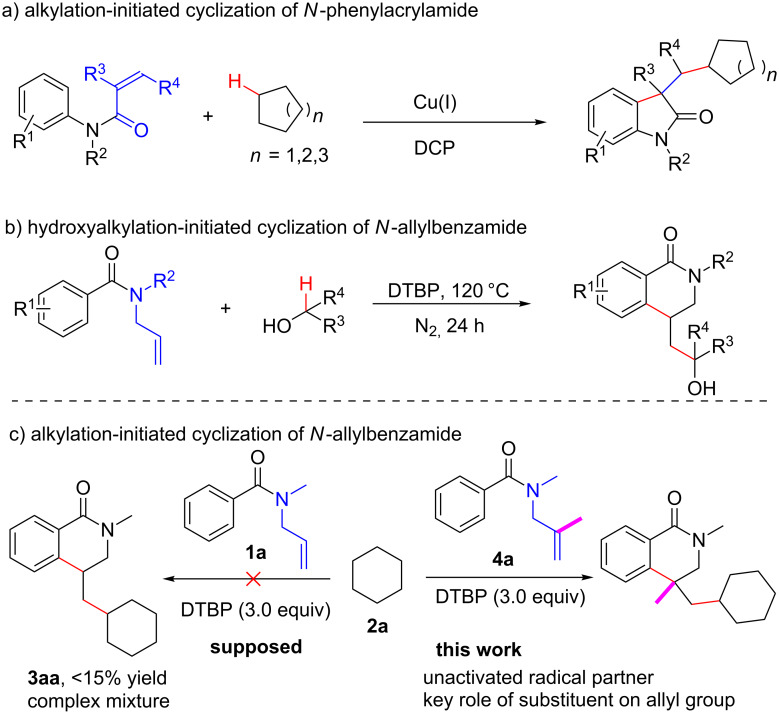
Cascade 1,2-difunctionalization and cyclization to construct heterocycles.

The direct cascade, 1,2-alkyarylation of alkenes to construct multi-substituted heterocycles has been considered as an efficient organic synthetic strategy, which is often featured by a new ring and dual C–C bond formation in one process [35–42]. Recently, the group of Liu reported a cascade alkylarylation of *N*-alkyl-*N*-phenylacryamide with simple alkanes resulting in alkyl-substituted oxindoles (Scheme 1a) [43]. However, cyclization of *N*-allylbenzamides initiated by the functionalization of sp^3^ C–H bonds of simple alkanes remains unexplored. Very recently, our group developed a metal-free hydroxyalkylation-initiated radical six-membered heterocycle formation reaction of *N*-allylbenzamide with alcohols as radical partners. This provided 4-hydroxyalkyl-substituted 3,4-dihydroisoquinolin-1(2*H*)-one derivatives (Scheme 1b) [44].

Based on the knowledge gained from previous reports on the cyclization of *N*-allylbenzamide [44], we envisioned that the unactivated cycloalkanes (instead of alcohols) could act as radical partners for this system. However, the reaction gave a complex mixture with 15% chemical yield of the expected product (Scheme 1c). Fortunately, when a methyl substituent was introduced onto the C–C double bond of the *N*-allylbenzamide substrate, the cyclization reaction proceeded smoothly (Scheme 1c). Herein, we report a metal-free cascade 1,2-alkyarylation of substituted *N*-allylbenzamides with alkanes affording 4-alkyl-substituted dihydroisoquinoline-1(2*H*)-ones as the product.

## Results and Discussion

Initially, we selected *N*-methyl-*N*-(2-methylallyl)benzamide (**4a**) and cyclohexane (**2a**) as model compounds (Table 1). As shown in Table 1, we found that the reactions did not happen or gave only a trace amount of the desired product with K_2_S_2_O_8_, AIBN, BPO and TBHP as oxidants (Table 1, entries 1, 3–5). PhI(OAc)_2_ and DCP could be used as oxidants, providing a slightly better yield (Table 1, entries 2 and 6). Dramatically higher chemical yields were found when TBPA and TBPB were used for this reaction (Table 1, entries 7 and 8). DTBP was the best oxidant choice, which afforded the highest chemical yield (53%, Table 1, entry 9). Then, a series of transition metal catalysts, including CuI, FeCl_2_, FeBr_2_ and FeCl_3_, were added into the reaction with DTBP as the oxidant. However, no improvement was observed at all. Finally, an attempt to shorten the reaction time to 24 h or to prolong the reaction time to 72 h resulted in lower yield, thus indicating that 48 h was appropriate for the completion of the reaction (Table 1, entries 14 and 15). Changing the amount of DTBP was also not successful. This is shown by the results presented in Table 1, entries 16 and 17 that clearly suggest that 3.0 equiv is the best choice. Finally, the reaction temperature was examined, and a lower chemical yield was found when the reaction was performed at 100 °C (Table 1, entry 16).

**Table 1:** Optimization of typical reaction conditions.^a^

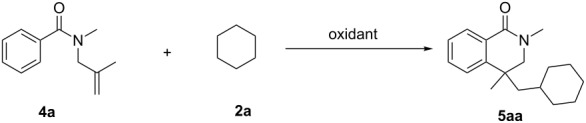					

entry	oxidant (equiv)	catalyst (mol %)	temp (°C)	time (h)	yield (%)^b^

1	K_2_S_2_O_8_ (3.0)	–	120	48	NR
2	PhI(OAc)_2_ (3.0)	–	120	48	19
3^c^	AIBN (3.0)	–	120	48	NR
4^c^	BPO (3.0)	–	120	48	trace
5^c^	TBHP (3.0)	–	120	48	trace
6^c^	DCP ( 3.0 )	–	120	48	19
7^c^	TBPA (3.0)	–	120	48	45
8^c^	TBPB (3.0)	–	120	48	38
9^c^	DTBP (3.0)	–	120	48	53
10	DTBP (3.0)	CuI (10)	120	48	49
11	DTBP (3.0)	FeCl_2_ (10)	120	48	40
12	DTBP (3.0)	FeBr_2_ (10)	120	48	45
13	DTBP (3.0)	FeCl_3_ (10)	120	48	33
14	DTBP (3.0)	–	120	24	25
15	DTBP (3.0)	–	120	72	50
16	DTBP (2.0)	–	120	48	30
17	DTBP (4.0)	–	120	48	46
18	DTBP (3.0)	–	100	48	43

^a^Reaction conditions: **4a** (0.2 mmol), cyclohexane (**2a**, 2.0 mL), oxidant, 120 °C, under N_2_. ^b^Isolated yield based on **4a**. ^c^AIBN = azodiisobutyronitrile; BPO = benzoyl peroxide; TBHP = *tert*-butyl hydroperoxide, 70% in water; DCP = dicumyl peroxide; TBPA = *tert*-butyl peracetate; TBPB = *tert*-butyl peroxybenzoate; DTBP = di-*tert*-butyl peroxide.

**Scheme 2:**
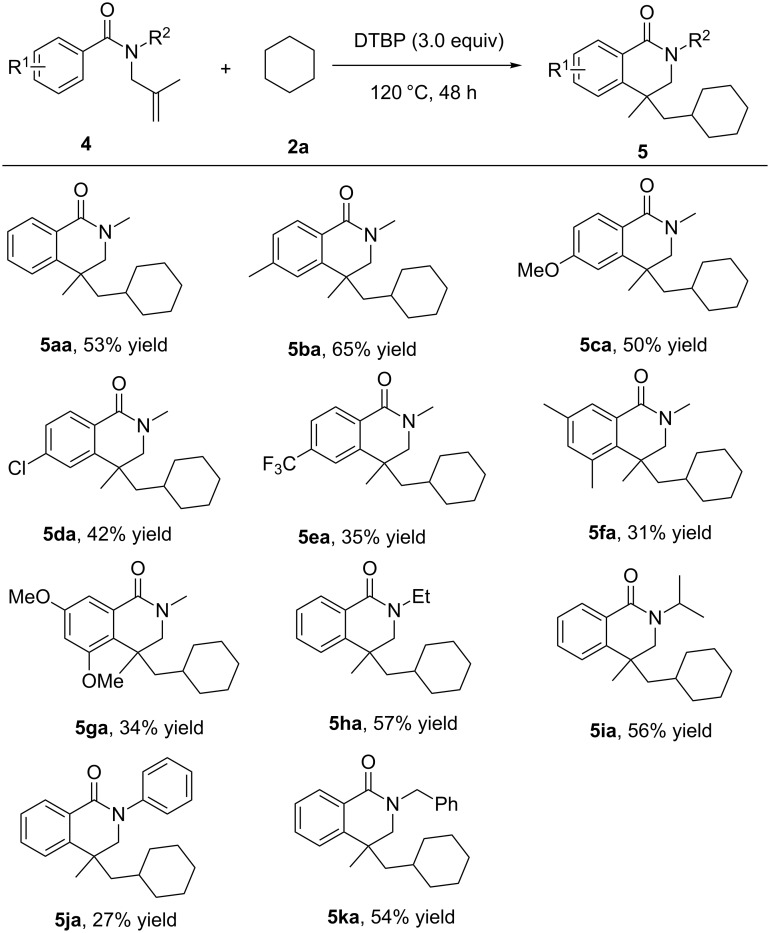
Cyclization of cyclohexane (**2a**) with substituted *N*-(2-methylallyl)benzamide (reaction conditions: **4** (0.2 mmol), cyclohexane (**2a**, 2 mL), DTBP (0.6 mmol), 120 °C, 48 h under nitrogen atmosphere. Isolated yield based on **4**).

With the optimized conditions developed, we then carried out a substrate generality study using various types of *N*-(2-methylallyl)benzamides **4** to react with cyclohexane (**2a**). As shown in Scheme 2, these cascade radical cyclization reactions are of general use for the preparation of 4-alkyldihydroisoquinolin-1(2*H*)-one derivatives **5**. The substrates bearing methyl, methoxy, halo and trifluoromethyl groups on the aromatic ring all worked well in the reaction, providing the target products with 31–65% yield. It should be noted that the reactions of substrates bearing disubstituted aromatic rings were possible but resulted in lower yield (**5fa** and **5ga**). On the other hand, the variation of the substituent on the nitrogen atom has also been examined. In the cases of *N*-ethyl (**4h**), *N*-isopropyl (**4i**), and *N*-benzyl (**4k**), no obvious effect was found and almost the same level of yield was obtained as for **4a**. However, a dramatically lower yield was obtained when a substrate with a *N*-phenyl group (**4j**) was used.

**Scheme 3:**
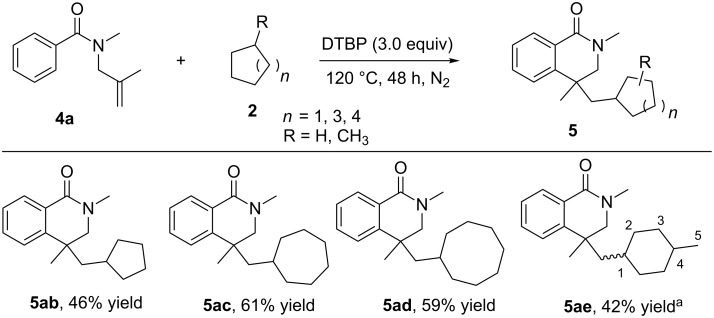
Cyclization of cycloalkanes with *N*-methyl-*N*-(2-methylallyl)benzamide (reaction conditions: **4a** (0.2 mmol), cycloalkanes **2** (2 mL), DTBP (0.6 mmol), 120 °C, 48 h under nitrogen atmosphere. Isolated yield based on **4a**). ^a^The total yield of isomers.

We then carried out another substrate scope examination for the radical reactions using various cycloalkanes **2** and *N*-methyl-*N*-(2-methylallyl)benzamide (**4a**). As indicated in Scheme 3, several cycloalkanes were well-tolerated in this radical reaction resulting in the corresponding product. In the case of cyclopentane (**2b**), a slightly lower chemical yield was obtained (46%, **5ab**), while the reactions of seven- and eight-membered ring cycloalkanes afforded the same level yield of the corresponding product (**5ac** and **5ad**). Finally, methylcyclohexane **2e** was used as the substrate for the investigation of the regioselectivity. The reaction almost showed no regio- and stereoselectivity and afforded the corresponding products (**5ae1–5ae5**) with 42% total yield.

**Scheme 4:**
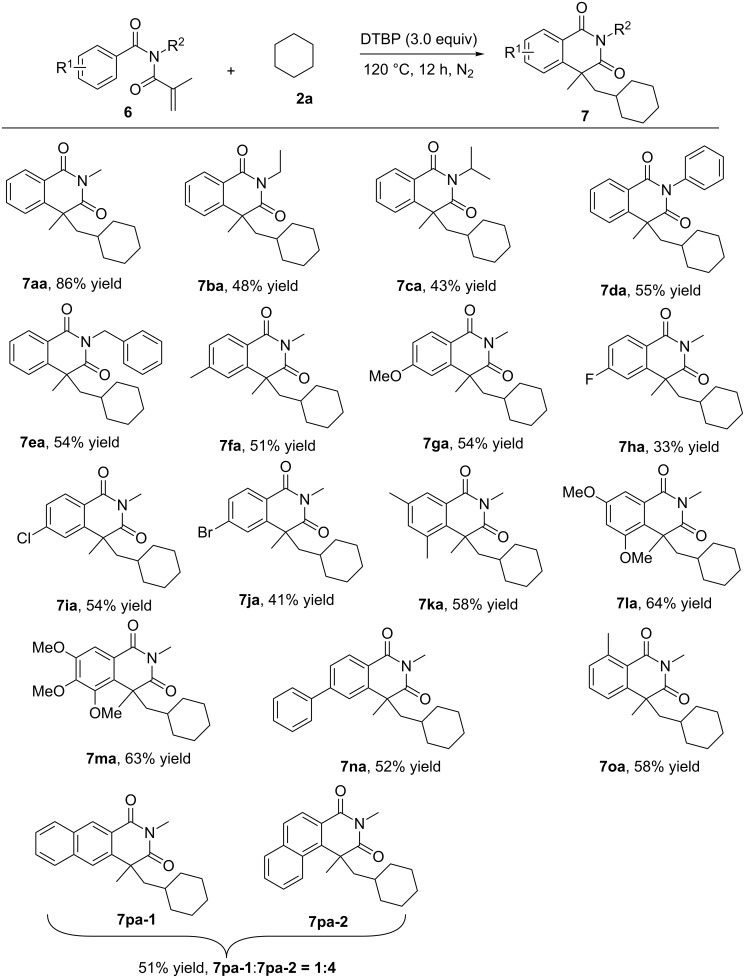
Cyclization reaction of **6** with cyclohexane **2a** (reaction conditions: **6** (0.2 mmol), cyclohexane **2a** (2 mL), DTBP (0.6 mmol), 120 °C, 12 h under nitrogen atmosphere. Isolated yield based on **6**).

To extend the synthetic utility of this radical cyclization reaction, *N*-methacryloyl-*N*-methylbenzamide derivatives **6** were then tried as substrates for this reaction (Scheme 4). It should be mentioned that only a few radical precursors, such as the TMSCF_3_ reagent [45] and ethers [46], were developed for such a cyclization system. Fortunately, the substrate with the introduction of a carbonyl group worked well in this system with moderate to good chemical yields (33–86%). Due to the existence of the carbonyl group, the reaction time could be shortened to 12 h where all of the starting material **6** is consumed. Firstly, the variation of the substituents on the nitrogen atom was investigated. We found that by changing the methyl group into one of the bulkier groups, the chemical yield significantly decreased (**7ba**–**7ea**). It is worth mentioning that the *N*-phenyl-*N*-methacryl-substituted substrate **6d** works much better than **4j** in this system, resulting in higher yield (55%, **5ja**). The substituent on the aromatic ring did not greatly affect the reaction efficiency, and methyl (**7fa** and **7ka**), methoxy (**7ga**, **7la** and **7ma**), chloro (**7ia**), bromo (**7ja**), and phenyl (**7na**) were well-tolerated in this system. However, in the case of the strong electron-withdrawing group (fluoro, **7ha**), the yield clearly decreased, and only 31% yield was obtained. It was noted that the reactions showed almost no evident regioselectivity, and the ratio of 1:4 (**7pa-1**:**7pa-2**) was found.

**Scheme 5:**
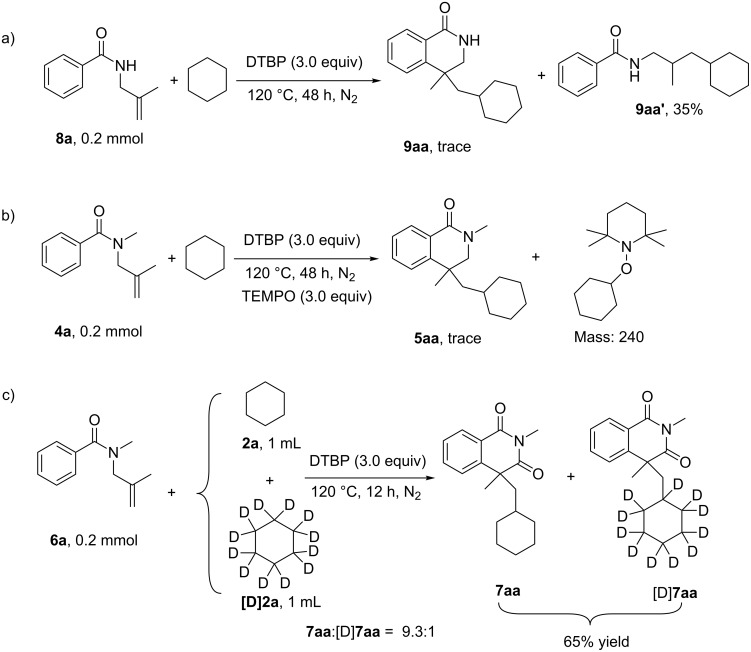
Control experiments for the mechanism studies. a) Reaction with *N*-unprotected substrate **8a**; b) reaction with the addition of radical-trapping reagent TEMPO; c) KIE study.

The final study of this reaction was the investigation of the mechanism. Firstly, a substrate (**8a**) bearing a hydrogen atom at the nitrogen was tried for the current system. The cyclohexane radical addition product **9aa’**, instead of a cyclization product, was observed with 35% yield (Scheme 5a). This result is consistent with our previous report [19], which discloses that the alkylation of the C–C double bond initiates the radical process. Furthermore, a radical-trapping reagent, 2,2,6,6-tetramethyl-1-piperidinyloxy (TEMPO), was added to the reaction, and the reaction was completely inhibited, affording a cyclohexane radical-trapped compound (Scheme 5b). This implies that the current transformation is a radical process. Finally, an obvious competing kinetic isotope effect (KIE) was found with the ratio of 9.3:1 (*k*_H_:*k*_D_) when the reaction of **6a** was performed with cyclohexane and [D]-cyclohexane (Scheme 5c). This discloses that the cleavage of the C(sp^3^)−H bond to form the radical may be involved in the turnover-limiting steps of this procedure.

**Scheme 6:**
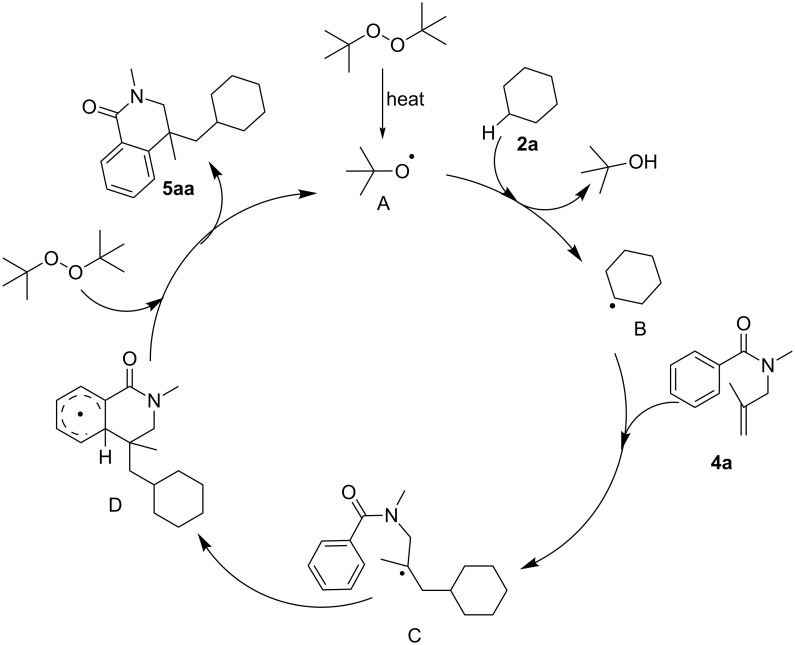
Proposed mechanism.

Based on the previous radical cyclization reactions [44,47–48] and the results obtained above, a plausible mechanism accounting for this cascade radical cyclization reaction was proposed (Scheme 6). Initially, DTBP undergoes homolytic cleavage to form the *tert*-butoxy radical **A**, which reacts with cyclohexane (**2a**) affording intermediate **B**. Then, intermediate **B** adds to *N*-methyl-*N*-(2-methylallyl)benzamide (**4a**), giving radical intermediate **C**. Intermediate **C** proceeds through intramolecular cyclization to give intermediate **D**. Finally, H-atom abstraction occurs between **D** and TBPB directly, which gives the product **5aa** and regenerates radical **A** for the next cycle.

## Conclusion

In summary, a metal-free cascade functionalization of unactivated C(sp^3^)–H bonds and cyclization reactions of *N*-substituted allylbenzamides were developed. The reaction involved cleavage of the C(sp^3^)–H bond, alkylation and intramolecular cyclization, affording the 4-alkyl-substituted dihydroisoquinolin-1(2*H*)-one derivatives with moderate to good chemical yield. The substituent on the C–C double bond was found to play a key role for the formation of the desired products. Also, *N*-methacryloylbenzamides worked well in the current reaction, which provides an easy way for the preparation of alkyl-subsituted isoquinoline-1,3(2*H*,4*H*)-diones.

## Experimental

**General procedure for the radical cyclization between *N*-allylbenzamide and *N*-methacryloylbenzamides with cycloalkanes:** Into an oven-dried reaction vial flushed with N_2_, substrate **4** or **6** (0.2 mmol), cycloalkanes **2** (2 mL), and DTBP (0.6 mmol) were added. Then the reaction mixture was stirred for 12–48 h at 120 °C under nitrogen atmosphere. After cooling, the reaction was quenched by a saturated NaCl solution (1 × 5 mL). Ethyl acetate (30 mL) was added to the system, and the mixture was washed with water (1 × 30 mL) and brine solution (1 × 30 mL). After drying over anhydrous Na_2_SO_4_, the solvent was removed. The crude mixture was charged onto silica gel and purified by flash chromatography to furnish the corresponding products **5** and **7**.

## Supporting Information

File 1 Experimental details and spectral data.
