# Supplementary material for: Cascade alkylarylation of substituted N-allylbenzamides for the construction of dihydroisoquinolin-1(2H)-ones and isoquinoline-1,3(2H,4H)-diones
Source: Beilstein J Org Chem. 2016 Feb 17;12:301–8. doi: 10.3762/bjoc.12.32 (PMC4778529; doi:10.3762/bjoc.12.32)

Supporting Information

for

Cascade alkylarylation of substituted *N*-allylbenzamides for the construction of dihydroisoquinolin-1(2*H*)-ones and isoquinoline-1,3(2*H*,4*H*)-diones

Ping Qian[1], Bingnan Du[1], Wei Jiao[1,2], Haibo Mei[1], Jianlin Han*[1] and Yi Pan*[1]

Address: [1]School of Chemistry and Chemical Engineering, State Key Laboratory of Coordination Chemistry, Nanjing University, Nanjing, 210093, China and [2]Institute for Chemistry & BioMedical Sciences, Nanjing University, Nanjing, 210093, China

Email: Jianlin Han* - hanjl@nju.edu.cn; Yi Pan - yipan@nju.edu.cn

*Corresponding author

Experimental details and spectral data

1. **General information...S2**

2. **Characterization data for compound 3, 5, 7 and 9.......................................S2**

3. **ESI-Mass analysis of TEMPO adduct product..S16**

4. **KIE studies of the cyclization reaction.. S17**

5. **^{1}H and ^{13}C NMR spectra for compound 3, 5, 7 and 9..................................S18**

1. General informaiton

The reactions were conducted under an atmosphere of N_2 and were monitored by TLC. Solvents were dried and distilled prior to use. Flash chromatography was performed using silica gel 60 (300–400 mesh). ^{1}H, ^{13}C and ^{19}F NMR spectra were recorded on a Bruker AVANCE400M spectrometer. Melting points were uncorrected. Infrared spectra were obtained on a Bruker Vector 22 in KBr pellets. HRMS were conducted on an Agilent 6540Q-TOF LC/MS equipped with an electrospray ionization (ESI) probe operating in positive ion mode.

2. Characterization data for compound 3, 5, 7 and 9

*4-(Cyclohexylmethyl)-2-methyl-3,4-dihydroisoquinolin-1(2H)-one (**3aa**)*: Colorless oil. ^{1}H NMR (400 MHz, $CDCl_3$) δ 8.07 (dd, J = 7.7, 1.1 Hz, 1H), 7.41 (td, J = 7.5, 1.4 Hz, 1H), 7.32 (td, J = 7.6, 1.0 Hz, 1H), 7.13 (d, J = 7.4 Hz, 1H), 3.77 (dd, J = 12.5, 4.3 Hz, 1H), 3.26 (dd, J = 12.5, 3.2 Hz, 1H), 3.15 (s, 3H), 2.95 (m, 1H), 1.75–1.42 (m, 6H), 1.50–1.42 (m, 2H), 1.19–0.95 (m, 3H), 0.94–0.85 (m, 2H). ^{13}C NMR (101 MHz, $CDCl_3$) δ 164.5, 142.8, 131.4, 128.6, 128.3, 126.8, 126.4, 52.3, 41.3, 35.5, 34.8, 34.5, 33.8, 33.0, 26.5, 26.2, 26.1. HRMS (TOF MS ESI): calcd for $C_{17}H_{24}NO$ $[M+H]^+$ 258.1858, found 258.1857.

*4-(Cyclohexylmethyl)-2,4-dimethyl-3,4-dihydroisoquinolin-1(2H)-one (**5aa**)*: Colorless oil (28.7 mg, 53%), ^{1}H NMR (400 MHz, $CDCl_3$) δ 8.09 (dd, J = 7.7, 1.4 Hz, 1H), 7.44 (td, J = 7.6, 1.5 Hz, 1H), 7.32 (td, J = 7.6, 1.1 Hz, 1H), 7.27 (m, 1H), 3.43 (d, J = 12.5 Hz, 1H), 3.22 (d, J = 12.5 Hz, 1H), 3.15 (s, 3H), 1.56–1.54 (m, 7H), 1.32 (s, 3H), 1.19–1.14 (m, 2H), 1.08–1.04 (m, 2H), 0.97–0.93 (m, 1H), 0.78–0.76 (m, 1H). ^{13}C NMR (101 MHz, $CDCl_3$) δ 164.6, 146.0, 131.5, 128.4, 128.2, 126.6, 124.0, 58.7, 46.6, 37.5, 35.7, 35.4, 35.3, 33.8, 26.5, 26.2, 26.0, 23.5. IR (cm^{-1}): ν 2922, 2850, 1651, 1603, 1494, 1448, 1332, 1256, 1081, 762, 702. HRMS (TOF MS ESI):

calcd for $C_{18}H_{26}NO$ $[M+H]^+$ 272.2014, found 272.2013.

*4-(Cyclohexylmethyl)-2,4,6-trimethyl-3,4-dihydroisoquinolin-1(2H)-one (**5ba**)***:** Colorless oil (37.0 mg, 65%). ^{1}H NMR (400 MHz, $CDCl_3$) δ 7.97 (d, J = 7.9 Hz, 1H), 7.12 (dd, J = 7.9, 0.8 Hz, 1H), 7.06 (s, 1H), 3.40 (d, J = 12.5 Hz, 1H), 3.21 (d, J = 12.5 Hz, 1H), 3.14 (s, 3H), 2.39 (s, 3H), 1.62–1.54 (m, 7H), 1.31 (s, 3H), 1.24–1.12 (m, 2H), 1.09–1.06 (m, 2H), 0.98–0.96 (m, 1H), 0.83–0.80 (m, 1H). ^{13}C NMR (101 MHz, $CDCl_3$) δ 164.7, 146.2, 141.9, 128.5, 127.4, 125.6, 124.6, 58.6, 46.6, 37.4, 35.7, 35.5, 35.2, 33.8, 26.5, 26.2, 26.0, 23.4, 21.8. IR (cm^{-1}): v 2922, 2850, 1651, 1611, 1488, 1448, 1398, 1331, 1258, 1067, 837, 781, 704. HRMS (TOF MS ESI): calcd for $C_{19}H_{28}NO$ $[M+H]^+$ 286.2171, found 286.2172.

*4-(Cyclohexylmethyl)-6-methoxy-2,4-dimethyl-3,4-dihydroisoquinolin-1(2H)-one (**5ca**)* **:** Colorless oil (30.2 mg, 50%), ^{1}H NMR (400 MHz, $CDCl_3$) δ 8.05 (d, J = 8.6 Hz, 1H), 6.83 (dd, J = 8.6, 2.4 Hz, 1H), 6.76 (d, J = 2.4 Hz, 1H), 3.85 (s, 3H), 3.39 (d, J = 12.4 Hz, 1H), 3.22 (d, J = 12.4 Hz, 1H), 3.13 (s, 3H), 1.57–1.49 (m, 7H), 1.30 (s, 3H), 1.26–1.22 (m, 2H), 1.10–1.03 (m, 2H), 0.96–0.94 (m, 1H), 0.86–0.83 (m, 1H). ^{13}C NMR (101 MHz, $CDCl_3$) δ 162.2, 148.3, 130.6, 121.2, 113.5, 111.1, 110.1, 58.6, 55.3, 46.4, 37.7, 35.7, 35.5, 35.1, 33.8, 26.5, 26.3, 26.0, 23.6. IR (cm^{-1}): v 2922, 2849, 1651, 1605, 1486, 1447, 1333, 1259, 1036, 779, 703. HRMS (TOF MS ESI): calcd for $C_{19}H_{27}NO_2Na$ $[M+Na]^+$ 324.1939, found 324.1940.

*6-Chloro-4-(cyclohexylmethyl)-2,4-dimethyl-3,4-dihydroisoquinolin-1(2H)-one (**5da**):* Colorless oil (25.8 mg, 42%), ^{1}H NMR (400 MHz, $CDCl_3$) δ 8.01 (d, J = 8.3 Hz, 1H), 7.29–7.26 (m, 1H), 7.22 (d, J = 2.0 Hz, 1H), 3.41 (d, J = 12.6 Hz, 1H), 3.22 (d, J = 12.6 Hz, 1H), 3.13 (s, 3H), 1.30–1.18 (m, 6H), 1.30 (s, 3H), 1.20–1.18 (m, 3H), 1.08–1.00 (m, 2H), 0.97–0.92 (m, 1H),

0.80–0.79 (m, 1H). ^{13}C NMR (101 MHz, $CDCl_3$) δ 163.7, 148.1, 137.8, 130.1, 126.9, 126.7, 124.3, 58.4, 46.4, 37.7, 35.7, 35.5, 35.3, 33.8, 26.4, 26.2, 25.9, 23.3. IR (cm^{-1}): ν 2923, 2851, 1660, 1595, 1494, 1448, 1332, 1259, 1163, 779, 698. HRMS (TOF MS ESI): calcd for $C_{18}H_{24}ClNONa$ $[M+Na]^+$ 328.1444, found 328.1446.

4-(Cyclohexylmethyl)-2,4-dimethyl-6-(trifluoromethyl)-3,4-dihydroisoquinolin-1(2H)-one ***(5ea)****:* Colorless oil (23.7 mg, 35%), ^{1}H NMR (400 MHz, $CDCl_3$) δ 8.20 (d, J = 8.1 Hz, 1H), 7.57 (d, J = 8.1 Hz, 1H), 7.50 (s, 1H), 3.46 (d, J = 12.6 Hz, 1H), 3.27 (d, J = 12.6 Hz, 1H), 3.17 (s, 3H), 1.55–1.47 (m, 7H), 1.36 (s, 3H), 1.17–1.09 (m, 2H), 1.07–1.04 (m, 2H), 0.97–0.93 (m, 1H), 0.80–0.75 (m, 1H). ^{19}F NMR (376 MHz, $CDCl_3$) δ -62.87. ^{13}C NMR (101 MHz, $CDCl_3$) δ 163.2, 146.9, 133.6, 133.3, 133.0, 132.7, 131.2, 129.1, 127.9, 125.2, 123.5, 123.5, 122.5, 121.2, 121.2, 119.8, 58.4, 46.4, 37.7, 35.7, 35.4, 33.9, 26.4, 26.1, 25.9, 23.3. IR (cm^{-1}): ν 2924, 2852, 1666, 1501, 1449, 1332, 1167, 1077, 897, 854, 790, 702. HRMS (TOF MS ESI): calcd for $C_{19}H_{24}F_3NONa$ $[M+Na]^+$ 362.1708, found 362.1708.

4-(Cyclohexylmethyl)-2,4,5,7-tetramethyl-3,4-dihydroisoquinolin-1(2H)-one ***(5fa)****:* Colorless oil (18.3 mg, 31%), ^{1}H NMR (400 MHz, $CDCl_3$) δ 7.83 (d, J = 1.3 Hz, 1H), 7.05 (d, J = 1.3 Hz, 1H), 3.40 (d, J = 12.7 Hz, 1H), 3.13–3.10 (m, 4H), 2.43 (s, 3H), 2.31 (s, 3H), 1.62–1.52 (m, 5H), 1.50–1.41 (m, 2H), 1.37 (s, 3H), 1.34–1.20 (m, 2H), 1.14–1.11 (m, 1H), 0.97–0.90 (m, 2H), 0.87–0.84 (m, 1H). ^{13}C NMR (101 MHz, $CDCl_3$) δ 165.4, 142.2, 137.0, 135.9, 134.6, 129.1, 127.5, 58.6, 44.3, 38.8, 35.7, 35.7, 35.0, 34.0, 26.5, 26.4, 26.1, 24.3, 23.0, 20.6. IR (cm^{-1}): ν 2922, 2851, 1658, 1608, 1495, 1448, 1335, 1292, 870, 789, 721. IR (cm^{-1}): ν 2922, 2851, 1658, 1608, 1495, 1448, 1335, 1292, 870, 789, 721. HRMS (TOF MS ESI): calcd for $C_{20}H_{30}NO$ $[M+H]^+$ 300.2327,

found: 300.2340.

4-(Cyclohexylmethyl)-5,7-dimethoxy-2,4-dimethyl-3,4-dihydroisoquinolin-1(2H)-one ***(5ga):*** Colorless oil (22.6 mg, 34%), ^{1}H NMR (400 MHz, $CDCl_3$) δ 7.23 (s, 1H), 6.49 (s, 1H), 3.77 (s, 3H), 3.71 (s, 3H), 3.23 (d, *J* = 12.6 Hz, 1H), 3.11 (d, *J* = 12.5 Hz, 1H), 3.04 (s, 3H), 1.52–1.49 (m, 7H), 1.31 (s, 3H), 1.08–1.03 (m, 4H), 0.81–0.78 (m, 2H). ^{13}C NMR (101 MHz, $CDCl_3$) δ 164.7, 158.9, 158.2, 130.6, 127.1, 103.3, 103.1, 59.5, 55.5, 55.2, 45.5, 37.4, 35.7, 35.4, 35.2, 34.1, 26.5, 26.4, 26.2, 24.6. IR (cm^{-1}): *ν* 2922, 2849, 1657, 1605, 1579, 1456, 1335, 1200, 1151, 1083, 1048, 937, 786, 734. HRMS (TOF MS ESI): calcd for $C_{20}H_{30}NO_3$ $[M+H]^+$ 332.2226, found 332.2241.

4-(Cyclohexylmethyl)-2-ethyl-4-methyl-3,4-dihydroisoquinolin-1(2H)-one ***(5ha):*** Colorless oil (32.8 mg, 57%), ^{1}H NMR (400 MHz, $CDCl_3$) δ 8.08 (d, *J* = 7.6 Hz, 1H), 7.42 (t, *J* = 7.4 Hz, 1H), 7.30 (t, *J* = 7.5 Hz, 1H), 7.25 (d, *J* = 7.3 Hz, 1H), 3.76–3.72 (m, 1H), 3.71–3.50 (m, 1H), 3.40 (d, *J* = 12.4 Hz, 1H), 3.20 (d, *J* = 12.4 Hz, 1H), 1.51–1.34 (m, 7H), 1.31 (s, 3H), 1.21 (t, *J* = 7.1 Hz, 3H), 1.14 (d, *J* = 12.8 Hz, 2H), 1.05 (t, *J* = 10.0 Hz, 2H), 0.96 (d, *J* = 11.0 Hz, 1H), 0.75–0.72 (m, 1H). ^{13}C NMR (101 MHz, $CDCl_3$) δ 163.8, 145.7, 131.5, 128.5, 128.5, 126.6, 124.1, 56.5, 46.4, 42.2, 37.3, 35.7, 35.4, 33.8, 26.5, 26.2, 26.0, 23.7, 12.4. IR (cm^{-1}): *ν* 2923, 2851, 1655, 1606, 1485, 1449, 1308, 1226, 1100, 763, 703. HRMS (TOF MS ESI): calcd for $C_{19}H_{28}NO$ $[M+H]^+$ 286.2172, found 286.2177.

4-(Cyclohexylmethyl)-2-isopropyl-4-methyl-3,4-dihydroisoquinolin-1(2H)-one ***(5ia):*** Colorless oil (33.8 mg, 56%), ^{1}H NMR (400 MHz, $CDCl_3$) δ 8.10 (dd, *J* = 7.7, 1.3 Hz, 1H), 7.44 (td, *J* = 7.5, 1.5 Hz, 1H), 7.32 (td, *J* = 7.6, 1.2 Hz, 1H), 7.27–7.26 (m, 1H), 5.16–5.11 (m, 1H), 3.18 (q, *J* = 12.5 Hz, 2H), 1.61–1.55 (m, 6H), 1.39–1.37 (m, 1H), 1.33 (s, 3H), 1.21 (d, *J* = 6.8 Hz, 3H), 1.17

(d, J = 6.9 Hz, 3H), 1.02–0.72 (m, 4H), 0.70–0.66 (m, 1H). ^{13}C NMR (101 MHz, $CDCl_3$) δ 163.5, 145.0, 131.4, 128.9, 128.7, 126.6, 124.1, 50.2, 46.0, 43.4, 36.9, 35.7, 35.2, 33.6, 26.53, 26.1, 26.0, 24.0, 19.5, 19.3. IR (cm^{-1}): ν 2922, 2850, 1653, 1603, 1485, 1448, 1380, 1332, 1226, 1091, 762, 700. HRMS (TOF MS ESI): calcd for $C_{20}H_{30}NO$ $[M+H]^+$ 300.2327, found 300.2332.

4-(cyclohexylmethyl)-4-methyl-2-phenyl-3,4-dihydroisoquinolin-1(2H)-one ***(5ja):*** Colorless oil (18.2 mg, 27%), ^{1}H NMR (400 MHz, $CDCl_3$) δ 8.10 (dd, J = 7.7, 1.2 Hz, 1H), 7.43 (td, J = 7.6, 1.5 Hz, 1H), 7.37–7.32 (m, 7H), 7.18–7.16 (m, 1H), 3.80 (d, J = 12.3 Hz, 1H), 3.57 (d, J = 12.3 Hz, 1H), 1.65–1.44 (m, 7H), 1.32 (s, 3H), 0.99–0.76 (m, 5H), 0.73–0.67 (m, 1H). ^{13}C NMR (101 MHz, $CDCl_3$) δ 164.0, 146.2, 143.1, 132.1, 129.0, 129.0, 128.6, 126.8, 126.3, 125.3, 124.2, 60.0, 46.5, 38.0, 35.7, 35.4, 33.7, 26.4, 26.2, 26.0, 23.7. IR (cm^{-1}): ν 2922, 2850, 1661, 1598, 1495, 1471, 1411, 1323, 1259, 761, 695. HRMS (TOF MS ESI): calcd for $C_{23}H_{27}NONa$ $[M+Na]^+$ 356.1990, found: 356.2003.

2-Benzyl-4-(cyclohexylmethyl)-4-methyl-3,4-dihydroisoquinolin-1(2H)-one ***(5ka):*** Colorless oil (37.9 mg, 54%), ^{1}H NMR (400 MHz, $CDCl_3$) δ 8.16 (dd, J = 7.7, 1.3 Hz, 1H), 7.45 (td, J = 7.6, 1.5 Hz, 1H), 7.30–7.24 (m, 7H), 4.89 (d, J = 14.4 Hz, 1H), 4.66 (d, J = 14.4 Hz, 1H), 3.32 (d, J = 12.5 Hz, 1H), 3.16 (d, J = 12.5 Hz, 1H), 1.48–1.45 (m, 5H), 1.29–1.26 (m, 1H), 1.23 (s, 3H), 1.11–1.02 (m, 3H), 1.01–0.99 (m, 2H), 0.83–0.77 (m, 1H), 0.68–0.65 (m, 1H). ^{13}C NMR (101 MHz, $CDCl_3$) δ 164.3, 146.0, 137.0, 131.7, 128.7, 128.6, 128.6, 128.2, 127.5, 126.6, 124.2, 56.2, 50.8, 46.5, 37.3, 35.5, 35.2, 33.6, 26.4, 26.1, 26.0, 23.6. IR (cm^{-1}): ν 2921, 2849, 1651, 1603, 1481, 1448, 1311, 1254, 1155, 1078, 1030, 762, 702. HRMS (TOF MS ESI): calcd for $C_{24}H_{30}NO$ $[M+H]^+$ 348.2327, found: 348.2331.

*4-(Cyclopentylmethyl)-2,4-dimethyl-3,4-dihydroisoquinolin-1(2H)-one (**5ab**):* Colorless oil (23.8 mg, 46%), ^{1}H NMR (400 MHz, $CDCl_3$) δ 8.02 (dd, J = 7.7, 1.2 Hz, 1H), 7.39–7.37 (m, 1H), 7.26–7.24 (m, 1H), 7.19 (d, J = 7.9 Hz, 1H), 3.38 (d, J = 12.5 Hz, 1H), 3.16 (d, J = 12.5 Hz, 1H), 3.08 (s, 3H), 1.66–1.51 (m, 4H), 1.43–1.33 (m, 5H), 1.27 (s, 3H), 0.83–0.80 (m, 1H), 0.78–0.73 (m, 1H). ^{13}C NMR (101 MHz, $CDCl_3$) δ 164.6, 146.1, 131.6, 128.4, 128.2, 126.6, 124.1, 58.9, 46.0, 37.5, 36.2, 35.3, 34.8, 34.6, 25.2, 25.0, 23.6. IR (cm^{-1}): ν 2949, 2868, 1652, 1603, 1494, 1466, 1400, 1333, 1299, 1257, 1035, 764, 702. HRMS (TOF MS ESI): calcd for $C_{17}H_{24}NO$ [M+H]$^+$ 258.1858, found 258.1865.

*4-(Cycloheptylmethyl)-2,4-dimethyl-3,4-dihydroisoquinolin-1(2H)-one (**5ac**):* Colorless oil (35.0 mg, 61%), ^{1}H NMR (400 MHz, $CDCl_3$) δ 8.02 (dd, J = 7.7, 1.3 Hz, 1H), 7.37 (td, J = 7.6, 1.4 Hz, 1H), 7.24 (td, J = 7.6, 1.1 Hz, 1H), 7.22–7.19 (m, 1H), 3.39 (d, J = 12.5 Hz, 1H), 3.39 (d, J = 12.5 Hz, 1H), 3.08 (s, 4H), 1.54–1.43 (m, 3H), 1.37–1.32 (m, 8H), 1.25 (s, 3H), 1.09–0.92 (m, 2H), 0.92–0.82 (m, 1H), 0.79–0.77 (m, 1H). ^{13}C NMR (101 MHz, $CDCl_3$) δ 164.6, 145.8, 131.5, 128.4, 128.2, 126.6, 124.1, 58.8, 47.7, 37.6, 37.5, 36.6, 35.3, 35.2, 28.4, 28.0, 26.4, 26.1, 23.2. IR (cm^{-1}): ν 2921, 2852, 1652, 1603, 1493, 1460, 1400, 1333, 1258, 1036, 764, 702. HRMS (TOF MS ESI): calcd for $C_{19}H_{28}NO$ [M+H]$^+$ 286.2171, found 286.2178.

*4-(Cyclooctylmethyl)-2,4-dimethyl-3,4-dihydroisoquinolin-1(2H)-one (**5ad**):* Colorless oil (35.1 mg, 59%), ^{1}H NMR (400 MHz, $CDCl_3$) δ 8.03–8.01 (m, 1H), 7.39–7.37 (m, 1H), 7.26–7.24 (m, 1H), 7.20–7.19 (m, 1H), 3.40 (d, J = 12.5 Hz, 1H), 3.10 (d, J = 15.0 Hz, 1H), 3.08 (s, 3H), 1.50–1.42 (m, 14H), 1.26 (s, 3H), 1.05–0.98 (m, 2H), 0.79–0.76 (m, 1H). ^{13}C NMR (101 MHz, $CDCl_3$) δ 164.6, 145.9, 131.5, 128.4, 128.2, 126.6, 124.2, 58.8, 47.9, 37.6, 35.3, 35.1, 34.4, 33.0,

27.1, 26.9, 26.3, 25.2, 25.1, 23.1. IR (cm^{-1}): v 2920, 2852, 1659, 1603, 1493, 1446, 1330, 1255, 1035, 763, 702. HRMS (TOF MS ESI): calcd for $C_{20}H_{30}NO$ $[M+H]^+$ 300.2327, found 300.2332.

2,4-Dimethyl-4-((4-methylcyclohexyl)methyl)-3,4-dihydroisoquinolin-1(2H)-one ***(5ae-1)****;*

2,4-Dimethyl-4-((3-methylcyclohexyl)methyl)-3,4-dihydroisoquinolin-1(2H)-one ***(5ae-2)****;*

2,4-Dimethyl-4-((2-methylcyclohexyl)methyl)-3,4-dihydroisoquinolin-1(2H)-one ***(5ae-3)****;*

2,4-Dimethyl-4-((1-methylcyclohexyl)methyl)-3,4-dihydroisoquinolin-1(2H)-one ***(5ae-4)****;*

4-(2-Cyclohexylethyl)-2,4-dimethyl-3,4-dihydroisoquinolin-1(2H)-one ***(5ae-5)****:* Colorless oil (24.2 mg, 42%), ^1H NMR (400 MHz, $CDCl_3$) δ 8.01 (dd, J = 10.5, 8.5 Hz, 1H), 7.36–7.34 (m, 1H), 7.26–7.22 (m, 1H), 7.20–7.18 (m, 1H), 3.39–3.35 (m, 1H), 3.15–3.09 (m, 1H), 3.08 (m, 3H), 1.70–1.44 (m, 1H), 1.41–1.26 (m, 7H), 1.25–1.18 (m, 3H), 0.93–0.81 (m, 1H), 0.74–0.66 (m, 6H). ^{13}C NMR (101 MHz, $CDCl_3$) δ 164.8, 164.6, 146.5, 131.5, 131.4, 128.5, 128.4, 128.3, 126.6, 126.6, 124.8, 124.0, 60.5, 46.8, 46.8, 46.5, 44.8, 44.4, 43.6, 43.2, 39.8, 39.3, 38.2, 37.5, 35.4, 35.3, 35.3, 34.8, 34.6, 29.7, 26.3, 26.1, 25.0, 21.9, 21.8. IR (cm^{-1}): v 2922, 2851, 1651, 1605, 1454, 1377, 1260, 1096, 1029, 802, 762, 703. HRMS (TOF MS ESI): calcd for $C_{19}H_{27}NONa$ $[M+Na]^+$ 308.1990, found 308.1987.

4-(Cyclohexylmethyl)-2,4-dimethylisoquinoline-1,3(2H,4H)-dione ***(7aa)****:* Colorless solid (49.0 mg, 86%), mp 49–52 °C, ^1H NMR (400 MHz, $CDCl_3$) δ 8.18 (d, J = 7.8 Hz, 1H), 7.58–7.54 (m, 1H), 7.35–7.21 (m, 2H), 3.31 (d, J = 1.2 Hz, 3H), 2.26 (dd, J = 14.0, 7.3 Hz, 1H), 1.83 (dd, J = 14.1, 4.5 Hz, 1H), 1.49 (s, 3H), 1.35–1.28 (m, 3H), 1.18–1.10 (m, 2H), 0.86–0.78 (m, 4H), 0.70–0.66 (m, 2H). ^{13}C NMR (101 MHz, $CDCl_3$) δ 176.8, 164.5, 143.9, 133.7, 128.8, 127.1, 125.7, 124.5, 49.5, 46.6, 34.8, 34.2, 32.9, 31.6, 27.1, 26.0, 25.9, 25.9. IR (cm^{-1}): v 2924, 2851, 1714, 1670, 1606, 1467, 1451, 1362, 1307, 1094, 1057, 768, 702. HRMS (TOF MS ESI): calcd for $C_{18}H_{23}NO_2Na$

[M+Na]$^+$ 308.1626, found 308.1621.

*4-(Cyclohexylmethyl)-2-ethyl-4-methylisoquinoline-1,3(2H,4H)-dione (**7ba**):* Colorless oil (28.7 mg, 48%), ^{1}H NMR (400 MHz, CDCl$_3$) δ 8.25 (dd, J = 7.9, 1.2 Hz, 1H), 7.61 (td, J = 7.7, 1.4 Hz, 1H), 7.41–7.26 (m, 2H), 4.06 (q, J = 7.1 Hz, 2H), 2.33 (dd, J = 14.0, 7.4 Hz, 1H), 1.89 (dd, J = 14.0, 4.8 Hz, 1H), 1.54 (s, 3H), 1.46–1.30 (m, 3H), 1.28–1.21 (m, 1H), 1.21 (t, J = 7.1 Hz, 3H), 1.13–0.96 (m, 1H), 0.90–0.86 (m, 4H), 0.76–0.75 (m, 2H). ^{13}C NMR (101 MHz, CDCl$_3$) δ 176.3, 164.0, 143.9, 133.6, 128.8, 127.1, 125.7, 124.7, 49.4, 46.5, 35.5, 34.9, 34.2, 33.1, 31.7, 26.0, 25.9, 12.9. IR (cm^{-1}): ν 2976, 2920, 1705, 1660, 1606, 1465, 1431, 1361, 1301, 1251, 1099, 1067, 889, 771, 707. HRMS (TOF MS ESI): calcd for $C_{19}H_{25}NO_2Na$ [M+Na]$^+$ 322.1783, found: 322.1781.

*4-(Cyclohexylmethyl)-2-isopropyl-4-methylisoquinoline-1,3(2H,4H)-dione (**7ca**):* Colorless oil (26.7 mg, 43%), ^{1}H NMR (400 MHz, CDCl$_3$) δ 8.23 (dd, J = 7.9, 1.1 Hz, 1H), 7.60 (td, J = 7.8, 1.4 Hz, 1H), 7.43–7.40 (m, 2H), 5.28–5.23 (m, 1H), 2.30 (dd, J = 14.0, 7.4 Hz, 1H), 1.86 (dd, J = 14.0, 4.9 Hz, 1H), 1.54 (s, 3H), 1.48–1.30 (m, 6H), 1.29–1.26 (m, 1H), 1.15–0.98 (m, 1H), 0.87–0.76 (m, 9H). ^{13}C NMR (101 MHz, CDCl$_3$) δ 176.7, 164.5, 143.8, 133.4, 128.8, 127.0, 125.6, 125.2, 49.5, 46.8, 45.3, 34.9, 34.2, 33.2, 31.4, 26.0, 26.0, 25.9, 19.5, 19.5. IR (cm^{-1}): ν 2925, 2852, 1712, 1667, 1605, 1451, 1398, 1354, 1302, 1252, 1099, 768, 706. HRMS (TOF MS ESI): calcd for $C_{20}H_{27}NO_2Na$ [M+Na]$^+$ 336.1939, found 336.1936.

*4-(Cyclohexylmethyl)-4-methyl-2-phenylisoquinoline-1,3(2H,4H)-dione (**7da**):* Colorless oil (38.1 mg, 55%), ^{1}H NMR (400 MHz, CDCl$_3$) δ 8.31–8.29 (dd, J = 7.8, 1.1 Hz, 1H), 7.71–7.51 (m, 1H), 7.50–7.46 (m, 5H), 7.19–7.18 (m, 2H), 2.40 (dd, J = 14.1, 7.7 Hz, 1H), 2.01 (dd, J = 14.1, 5.0 Hz, 1H), 1.69 (s, 3H), 1.51–1.26 (m, 3H), 1.27–1.11 (m, 2H), 0.98–0.84 (m, 6H). ^{13}C NMR (101 MHz, CDCl$_3$) δ 176.6, 164.4, 144.1, 135.6, 134.1, 129.3, 129.2, 128.5, 128.3, 127.3, 125.9, 124.6,

49.4, 47.2, 35.0, 34.2, 33.1, 31.7, 26.1, 26.0, 25.9. IR (cm^{-1}): ν 2923, 2851, 1721, 1679, 1605, 1491, 1463, 1364, 1303, 1246, 1196, 767, 694. HRMS (TOF MS ESI): calcd for $C_{23}H_{25}NO_2Na$ $[M+Na]^+$ 370.1783, found 370.1782.

*2-Benzyl-4-(cyclohexylmethyl)-4-methylisoquinoline-1,3(2H,4H)-dione (**7ea**):* Colorless oil (38.8 mg, 54%), ^1H NMR (400 MHz, $CDCl_3$) δ 8.27 (dd, J = 7.9, 1.2 Hz, 1H), 7.62 (td, J = 7.8, 1.5 Hz, 1H), 7.43–7.38 (m, 4H), 7.25–7.20 (m, 3H), 5.21 (dd, J = 42.4, 13.7 Hz, 2H), 2.30 (dd, J = 14.0, 7.2 Hz, 1H), 1.87 (dd, J = 14.0, 5.2 Hz, 1H), 1.55 (s, 3H), 1.35–1.25 (m, 3H), 1.06–0.92 (m, 2H), 0.92–0.82 (m, 4H), 0.66–0.63 (m, 2H). ^{13}C NMR (101 MHz, $CDCl_3$) δ 176.4, 164.2, 144.0, 137.0, 133.8, 129.0, 129.0, 128.3, 127.3, 127.1, 125.7, 124.5, 49.5, 46.8, 43.6, 34.8, 34.1, 33.2, 31.7, 25.9, 25.8, 25.8. IR (cm^{-1}): ν 3065, 3034, 2924, 2851, 1713, 1668, 1606, 1465, 1452, 1384, 1353, 1317, 1226, 1161, 1077, 767, 707. HRMS (TOF MS ESI): calcd for $C_{24}H_{27}NO_2Na$ $[M+Na]^+$ 384.1939, found 384.1936.

*4-(Cyclohexylmethyl)-2,4,6-trimethylisoquinoline-1,3(2H,4H)-dione (**7fa**):* Colorless solid (30.5 mg, 51%), mp 58 – 60 ^oC, ^1H NMR (400 MHz, $CDCl_3$) δ 8.12 (d, J = 8.0 Hz, 1H), 7.26–7.22 (m, 1H), 7.16 (s, 1H), 3.35 (s, 3H), 2.44 (s, 3H), 2.29 (dd, J = 14.0, 7.4 Hz, 1H), 1.86 (dd, J = 14.0, 4.8 Hz, 1H), 1.53 (s, 3H), 1.46–1.35 (m, 3H), 1.24–1.22 (m, 1H), 1.15–1.02 (m, 1H), 0.94–0.91 (m, 4H), 0.76–0.74 (m, 2H). ^{13}C NMR (101 MHz, $CDCl_3$) δ 177.0, 164.5, 144.5, 143.9, 128.8, 128.3, 126.0, 122.0, 77.3, 77.0, 76.7, 49.6, 46.5, 34.8, 34.2, 33.0, 31.6, 27.0, 26.0, 26.0, 25.9, 22.0. IR (cm^{-1}): ν 3362, 2924, 2851, 1713, 1668, 1614, 1450, 1427, 1356, 1307, 1354, 1094, 1056, 838, 779, 704. HRMS (TOF MS ESI): calcd for $C_{19}H_{25}NO_2Na$ $[M+Na]^+$ 322.1783, found 322.1781.

*4-(Cyclohexylmethyl)-6-methoxy-2,4-dimethylisoquinoline-1,3(2H,4H)-dione (**7ga**):* Colorless oil (34.2 mg, 54%), ^1H NMR (400 MHz, $CDCl_3$) δ 8.21 (d, J = 8.8 Hz, 1H), 6.96 (dd, J = 8.8, 2.5 Hz,

1H), 6.84 (d, *J* = 2.4 Hz, 1H), 3.90 (s, 3H), 3.36 (s, 3H), 2.32 (dd, *J* = 14.0, 7.5 Hz, 1H), 1.85 (dd, *J* = 14.0, 4.8 Hz, 1H), 1.55 (s, 3H), 1.49–1.30 (m, 3H), 1.25–1.19 (m, 2H), 0.94–0.92 (m, 4H), 0.79–0.75 (m, 2H). ^{13}C NMR (101 MHz, $CDCl_3$) δ 176.9, 164.1, 163.9, 146.2, 131.2, 117.6, 113.0, 110.8, 55.5, 49.7, 46.8, 34.8, 34.3, 33.0, 31.8, 27.0, 26.0, 26.0, 25.9. IR (cm^{-1}): ν 3207, 2925, 2851, 1708, 1666, 1606, 1456, 1435, 1358, 1303, 1251, 1035, 778, 702. HRMS (TOF MS ESI): calcd for $C_{19}H_{25}NO_3Na$ [M+Na]$^+$ 338.1732, found 338.1730.

*4-(Cyclohexylmethyl)-6-fluoro-2,4-dimethylisoquinoline-1,3(2H,4H)-dione (**7ha**):* Colorless solid (19.8 mg, 33%), mp 132 – 135 ^{o}C, ^{1}H NMR (400 MHz, $CDCl_3$) δ 8.28 (dd, *J* = 8.8, 5.9 Hz, 1H), 7.15–7.12 (m, 1H), 7.07 (dd, *J* = 9.6, 2.4 Hz, 1H), 3.37 (s, 3H), 2.37 (dd, *J* = 14.1, 7.4 Hz, 1H), 1.82 (dd, *J* = 14.1, 4.7 Hz, 1H), 1.55 (s, 3H), 1.49–1.25 (m, 3H), 1.17–0.97 (m, 2H), 0.91–0.88 (m, 4H), 0.79–0.77 (m, 2H). ^{13}C NMR (101 MHz, $CDCl_3$) δ 176.3, 167.5, 165.0, 163.5, 147.1, 147.0, 132.0, 131.9, 121.0, 121.0, 121.0, 115.3, 115.1, 112.6, 112.4, 49.6, 46.9, 34.8, 34.2, 32.9, 31.6, 27.2, 26.0, 26.0, 25.9. IR (cm^{-1}): ν 3071, 2920, 2849, 1708, 1666, 1616, 1455, 1435, 1358, 1307, 1220, 1092, 1057, 853, 783, 702. HRMS (TOF MS ESI): calcd for $C_{18}H_{22}FNO_2Na$ [M+Na]$^+$ 326.1532, found 326.1539.

*6-Chloro-4-(cyclohexylmethyl)-2,4-dimethylisoquinoline-1,3(2H,4H)-dione (**7ia**):* Colorless solid (34.3 mg, 54%), mp 102 – 105 ^{o}C, ^{1}H NMR (400 MHz, $CDCl_3$) δ 8.18 (d, *J* = 8.4 Hz, 1H), 7.39–7.36 (m, 2H), 3.36 (s, 3H), 2.32 (dd, *J* = 14.2, 7.4 Hz, 1H), 1.84 (dd, *J* = 14.1, 4.8 Hz, 1H), 1.54 (s, 3H), 1.47–1.30 (m, 3H), 1.18 (dd, *J* = 14.9, 12.6 Hz, 2H), 0.97–0.92 (m, 4H), 0.86–0.77 (m, 2H). ^{13}C NMR (101 MHz, $CDCl_3$) δ 176.1, 163.6, 145.7, 140.3, 130.5, 127.8, 125.9, 123.0, 49.5, 46.7, 34.8, 34.2, 32.9, 31.5, 27.2, 25.9, 25.9, 25.9. IR (cm^{-1}): ν 3075, 2919, 2850, 1708, 1665, 1598, 1458, 1428, 1356, 1306, 1096, 1057, 855, 783, 699. HRMS (TOF MS ESI): calcd for

$C_{18}H_{22}ClNO_2Na$ [M+Na]$^+$ 342.1237, found 342.1232.

6-Bromo-4-(cyclohexylmethyl)-2,4-dimethylisoquinoline-1,3(2H,4H)-dione ***(7ja):*** Colorless oil (29.9 mg, 41%), ^{1}H NMR (400 MHz, CDCl$_3$) δ 8.05 (d, *J* = 8.4 Hz, 1H), 7.50 (dd, *J* = 8.4, 1.8 Hz, 1H), 7.48 (d, *J* = 1.7 Hz, 1H), 3.30 (s, 3H), 2.26 (dd, *J* = 14.1, 7.3 Hz, 1H), 1.78 (dd, *J* = 14.1, 4.8 Hz, 1H), 1.40 (s, 3H), 1.39–1.35 (m, 3H), 1.12–0.95 (m, 2H), 0.86–0.82 (m, 4H), 0.71–070 (m, 2H). ^{13}C NMR (101 MHz, CDCl$_3$) δ 176.1, 163.8, 145.8, 130.8, 130.5, 129.1, 128.9, 123.5, 49.6, 46.7, 34.8, 34.3, 33.0, 31.6, 27.3, 26.0 , 26.0, 25.9. IR (cm^{-1}): ν 3073, 2919, 2850, 1709, 1666, 1593, 1574, 1456, 1355, 1305, 1097, 1058, 856, 783, 701. HRMS (TOF MS ESI): calcd for $C_{18}H_{23}BrNO_2$ [M+H]$^+$ 364.0912, found 364.0917.

4-(Cyclohexylmethyl)-2,4,5,7-tetramethylisoquinoline-1,3(2H,4H)-dione ***(7ka):*** Colorless oil (36.2 mg, 58%), ^{1}H NMR (400 MHz, CDCl$_3$) δ 8.03 (d, *J* = 0.8 Hz, 1H), 7.22 (dd, *J* = 1.4, 0.5 Hz, 1H), 3.36 (s, 3H), 2.50 (s, 3H), 2.35 (s, 3H), 2.38–2.23 (m, 1H), 2.24–2.22 (dd, *J* = 14.5, 3.6 Hz, 1H), 1.59 (s, 3H), 1.44–1.30 (m, 2H), 1.25–1.24 (m, 1H), 1.12–0.97 (m, 1H), 0.97–0.88 (m, 2H), 0.77–0.68 (m, 5H). ^{13}C NMR (101 MHz, CDCl$_3$) δ 178.1, 164.8, 139.5, 138.0, 136.7, 135.6, 128.0, 125.5, 47.8, 45.7, 35.2, 33.9, 32.3, 28.34, 27.5, 26.0, 25.9, 25.9, 22.6, 20.7. IR (cm^{-1}): ν 2924, 2852, 1709, 1666, 1614, 1447, 1426, 1364, 1321, 1294, 1103, 1019, 788, 582. HRMS (TOF MS ESI): calcd for $C_{20}H_{27}NO_2Na$ [M+Na]$^+$ 336.1939, found 336.1935.

4-(Cyclohexylmethyl)-5,7-dimethoxy-2,4-dimethylisoquinoline-1,3(2H,4H)-dione ***(7la):*** Colorless oil (44.4 mg, 64%), ^{1}H NMR (400 MHz, CDCl$_3$) δ 7.35 (d, *J* = 2.6 Hz, 1H), 6.64 (d, *J* = 2.6 Hz, 1H), 3.82 (s, 3H), 3.78 (s, 3H), 3.30 (s, 3H), 2.45 (dd, *J* = 13.8, 3.8 Hz, 1H), 2.07 (dd, *J* = 13.8, 6.9 Hz, 1H), 1.53 (s, 3H), 1.40–1.39 (m, 3H), 1.12–0.93 (m, 2H), 0.88–0.75 (m, 3H), 0.70–0.68 (m, 3H). ^{13}C NMR (101 MHz, CDCl$_3$) δ 178.2, 164.6, 159.4, 158.2, 126.6, 124.6, 105.3, 102.6, 55.6,

55.5, 46.5, 45.8, 35.5, 34.0, 32.9, 27.7, 27.4, 26.1, 26.0. IR (cm^{-1}): ν 2925, 2850, 1711, 1667, 1610, 1449, 1371, 1320, 1293, 1250, 1034, 777, 702. HRMS (TOF MS ESI): calcd for $C_{20}H_{27}NO_4Na$ $[M+Na]^+$ 368.1838, found 368.1835.

4-(Cyclohexylmethyl)-5,6,7-trimethoxy-2,4-dimethylisoquinoline-1,3(2H,4H)-dione ***(7ma)****:* Colorless oil (47.0 mg, 63%), ^1H NMR (400 MHz, $CDCl_3$) δ 7.59 (s, 1H), 3.95 (s, 3H), 3.94 (s, 3H), 3.89 (s, 3H), 3.34 (s, 3H), 2.39 (dd, J = 13.7, 3.4 Hz, 1H), 2.16 (dd, J = 13.8, 6.9 Hz, 1H), 1.61 (s, 3H), 1.45–1.43 (m, 3H), 1.18–1.15 (m, 2H), 0.97–0.93 (m, 3H), 0.77–0.73 (m, 3H). ^{13}C NMR (101 MHz, $CDCl_3$) δ 178.0, 164.1, 152.8, 151.5, 147.0, 129.3, 120.2, 106.4, 60.6, 60.5, 56.0, 46.7, 46.7, 35.5, 33.9, 32.7, 28.4, 27.3, 26.1, 26.0. IR (cm^{-1}): ν 2926, 2851, 1708, 1664, 1597, 1489, 1449, 1425, 1365, 1320, 1093, 1018, 771. HRMS (TOF MS ESI): calcd for $C_{21}H_{29}NO_5Na$ $[M+Na]^+$ 398.1943, found 398.1948.

4-(Cyclohexylmethyl)-2,4-dimethyl-6-phenylisoquinoline-1,3(2H,4H)-dione ***(7na)****:* Colorless oil (37.4 mg, 52%), ^1H NMR (400 MHz, $CDCl_3$) δ 8.32 (d, J = 8.2 Hz, 1H), 7.63–7.52 (m, 4H), 7.51–7.49 (m, 2H), 7.44–7.42 (m, 1H), 3.41 (s, 3H), 2.37 (dd, J = 14.1, 7.4 Hz, 1H), 1.98 (dd, J = 14.1, 4.9 Hz, 1H), 1.62 (s, 3H), 1.49–1.35 (m, 3H), 1.30–1.25 (m, 1H), 1.20–1.18 (m, 1H), 0.96–0.93 (m, 4H), 0.83–0.79 (m, 2H). ^{13}C NMR (101 MHz, $CDCl_3$) δ 176.8, 164.3, 146.5, 144.3, 139.8, 129.4, 129.0, 128.5, 127.3, 126.1, 124.2, 123.3, 49.7, 46.8, 34.9, 34.3, 33.1, 31.6, 27.1, 26.0, 25.9, 25.9. IR (cm^{-1}): ν 3070, 2926, 1708, 1667, 1609, 1459, 1422, 1353, 1304, 1169, 1053, 757, 705. HRMS (TOF MS ESI): calcd for $C_{24}H_{27}NO_2Na$ $[M+Na]^+$ 384.1939, found 384.1940.

4-(Cyclohexylmethyl)-2,4,8-trimethylisoquinoline-1,3(2H,4H)-dione ***(7oa)****:* Colorless oil (34.7 mg, 58%), ^1H NMR (400 MHz, $CDCl_3$) δ 7.45 (t, J = 7.7 Hz, 1H), 7.27 (d, J = 7.9 Hz, 1H), 7.21 (d, J

= 7.5 Hz, 1H), 3.34 (s, 3H), 2.79 (s, 3H), 2.30 (dd, *J* = 14.1, 7.5 Hz, 1H), 1.86 (dd, *J* = 14.1, 4.7 Hz, 1H), 1.55 (s, 3H), 1.47–1.30 (m, 3H), 1.18–1.10 (m, 2H), 0.93–0.87 (m, 4H), 0.76–0.71 (m, 2H). ^{13}C NMR (101 MHz, $CDCl_3$) δ 176.6, 165.0, 145.2, 142.4, 132.5, 131.1, 124.0, 122.9, 50.0, 46.6, 34.7, 34.3, 33.0, 31.8, 27.1, 26.0, 26.0, 26.0, 24.1. IR (cm^{-1}): ν 2925, 2852, 1710, 1668, 1595, 1473, 1451, 1356, 1314, 1287, 1055, 789, 705. HRMS (TOF MS ESI): calcd for $C_{19}H_{25}NO_2Na$ $[M+Na]^+$ 322.1783, found 322.1781.

4-(Cyclohexylmethyl)-2,4-dimethylbenzo[g]isoquinoline-1,3(2H,4H)-dione ***(7pa-1)*** *and 1-(Cyclohexylmethyl)-1,3-dimethylbenzo[f]isoquinoline-2,4(1H,3H)-dione* ***(7pa-2)****:* Colorless oil (34.2 mg, 51%), ^{1}H NMR (400 MHz, $CDCl_3$) δ 8.86 (s, 0.2 H, **7pa-1**), 8.53 (d, *J* = 8.2 Hz, 0.8H, **7pa-2**), 8.36 (dd, *J* = 8.7, 2.9 Hz, 0.8H, **7pa-2**), 8.02 (d, *J* = 8.2 Hz, 0.2 H, **7pa-1**), 7.89 (dt, *J* = 26.6, 10.0 Hz, 2H, **7pa-1** & **7pa-2**), 7.58–7.53 (m, 2H, **7pa-1** & **7pa-2**), 3.44 (s, 3H, **7pa-1** & **7pa-2**), 2.75 (dd, *J* = 14.4, 4.6 Hz, 0.8 H, **7pa-2**), 2.61 (dd, *J* = 14.4, 7.3 Hz, 0.8H, **7pa-2**), 2.39–2.36 (m, 0.2H, **7pa-1**), 2.02–1.96 (m, 0.2H, **7pa-1**), 1.95 (s, 2.4H, **7pa-2**), 1.66 (s, 0.6H, **7pa-1**), 1.33–1.15 (m, 5H, **7pa-1** & **7pa-2**), 0.96–0.86 (m, 4H, **7pa-1** & **7pa-2**), 0.65–0.57 (m, 2H, **7pa-1** & **7pa-2**). ^{13}C NMR (101 MHz, $CDCl_3$) δ 178.3, 176.9, 164.7, 164.6, 141.5, 138.9, 136.9, 135.9, 131.5, 130.8, 130.1, 130.0, 129.4, 129.0, 128.8, 127.9, 127.6, 126.8, 126.7, 126.4, 124.8, 124.1, 123.3, 122.5, 77.3, 77.0, 76.7, 50.5, 49.0, 48.6, 46.8, 35.4, 34.8, 34.4, 33.7, 33.0, 32.9, 32.0, 30.0, 27.4, 26.0, 25.9, 25.8, 25.8, 25.7. IR (cm^{-1}): ν 2925, 2851, 1709, 1666, 1633, 1472, 1434, 1377, 1321, 1085, 1035, 916, 834, 761, 733. HRMS (TOF MS ESI): calcd for $C_{22}H_{25}NO_2Na$ $[M+Na]^+$ 358.1783, found 358.1779.

N-(3-Cyclohexyl-2-methylpropyl)benzamide ***(9aa')****:* Colorless oil (18.1 mg, 35%), ^{1}H NMR (400

MHz, $CDCl_3$) δ 7.77–7.75 (m, 2H), 7.47–7.41 (m, 3H), 6.14 (s, 1H), 3.42–3.36 (m, 1H), 3.26–3.19 (m, 1H), 1.87–1.81 (m, 1H), 1.67–1.63 (m, 6H), 1.36–1.23 (m, 1H), 1.22–1.14 (m, 2H), 1.08–1.06 (m, 2H), 0.95 (d, *J* = 6.7 Hz, 3H), 0.84–0.82 (m, 2H). ^{13}C NMR (101 MHz, $CDCl_3$) δ 167.6, 135.0, 131.3, 128.6, 126.8, 46.3, 42.5, 34.8, 34.2, 33.0, 30.3, 26.6, 26.4, 26.3, 18.0. IR (cm^{-1}): ν 3320, 2923, 2850, 1640, 1541, 1489, 1448, 1293, 697. HRMS (ESI): calcd for $C_{17}H_{26}NO$ $[M+H]^+$ 260.2014, found 260.2015.

3. ESI-MS analysis of TEMPO adduct product

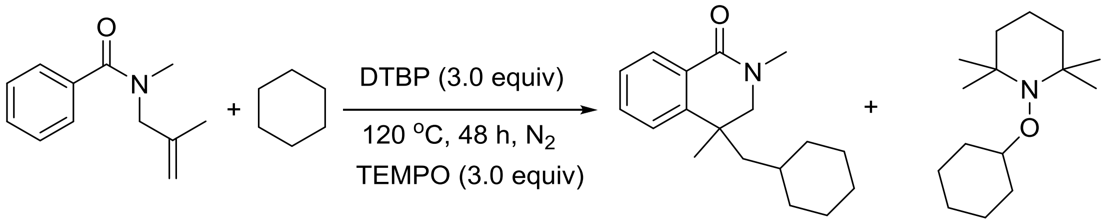

4a, 0.2 mmol   **5aa**, trace   Mass: 240

A mixture of *N*-methyl-*N*-(2-methylallyl)benzamide (**4a**, 0.2 mmol), cyclohexane (**2a**, 2 mL), DTBP (0.6 mmol), and TEMPO (0.6 mmol) was stirred at 120 °C under nitrogen atmosphere. After 48 h, the reaction mixture was subjected to ESI-MS (positive mode) spectroscopic analysis. Copied below is the ESI-MS spectrum we obtained. LCMS (ESI): calcd for $C_{15}H_{30}NO$ $[M + H]^+$ 240.23, found 240.15.

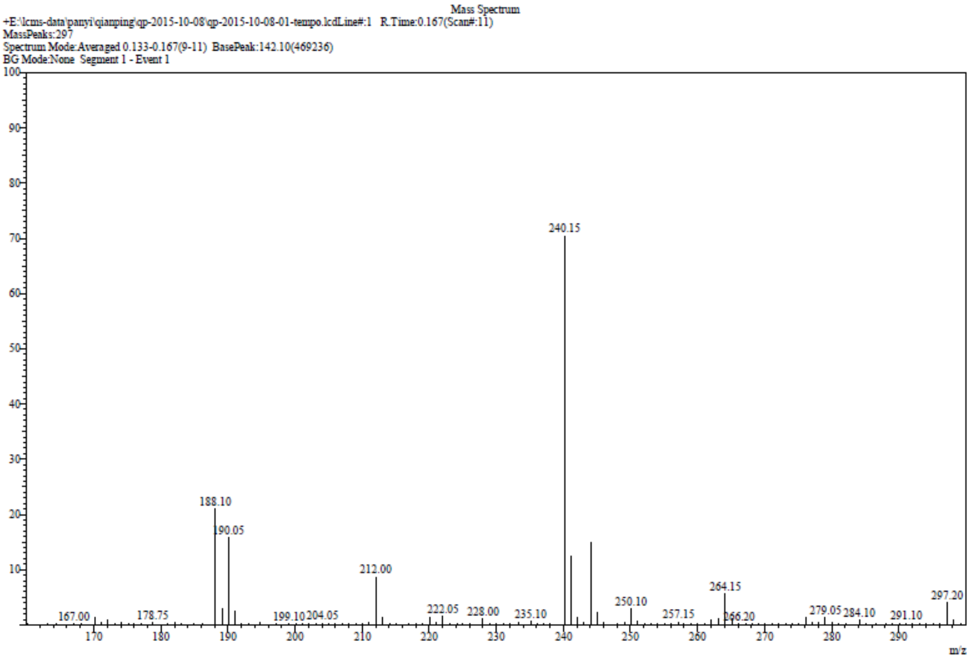

4. KIE studies of the cyclization reaction

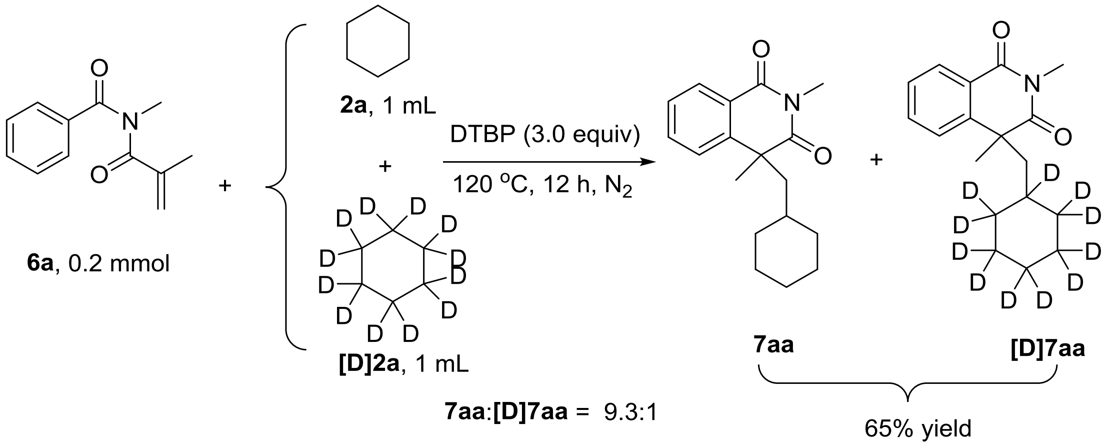

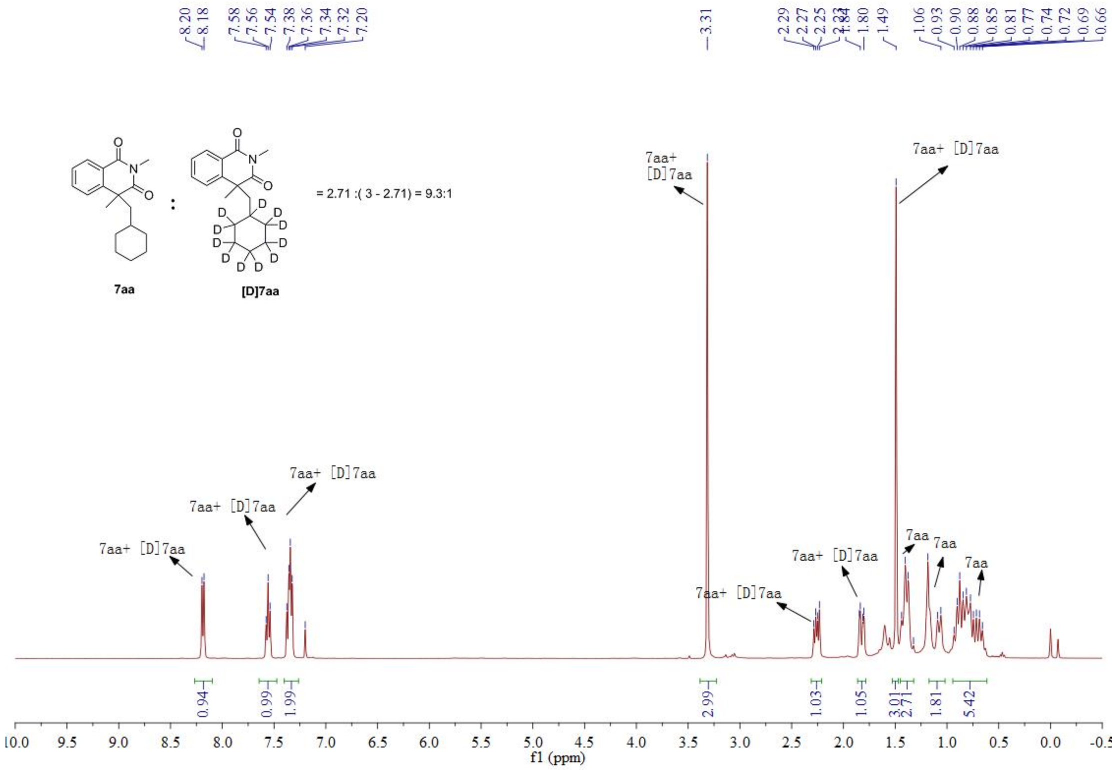

5. ^{1}H and ^{13}C NMR spectra for compound 3, 5, 7 and 9

^{1}H NMR and ^{13}C NMR spectrum of **3aa**

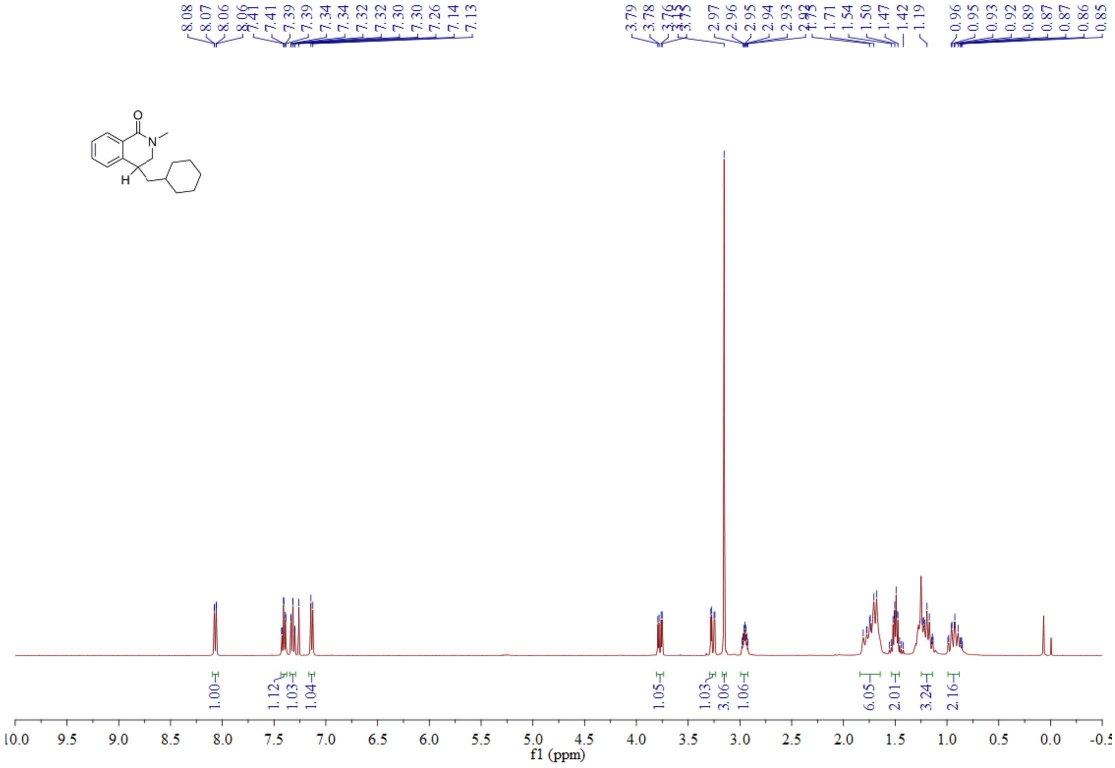

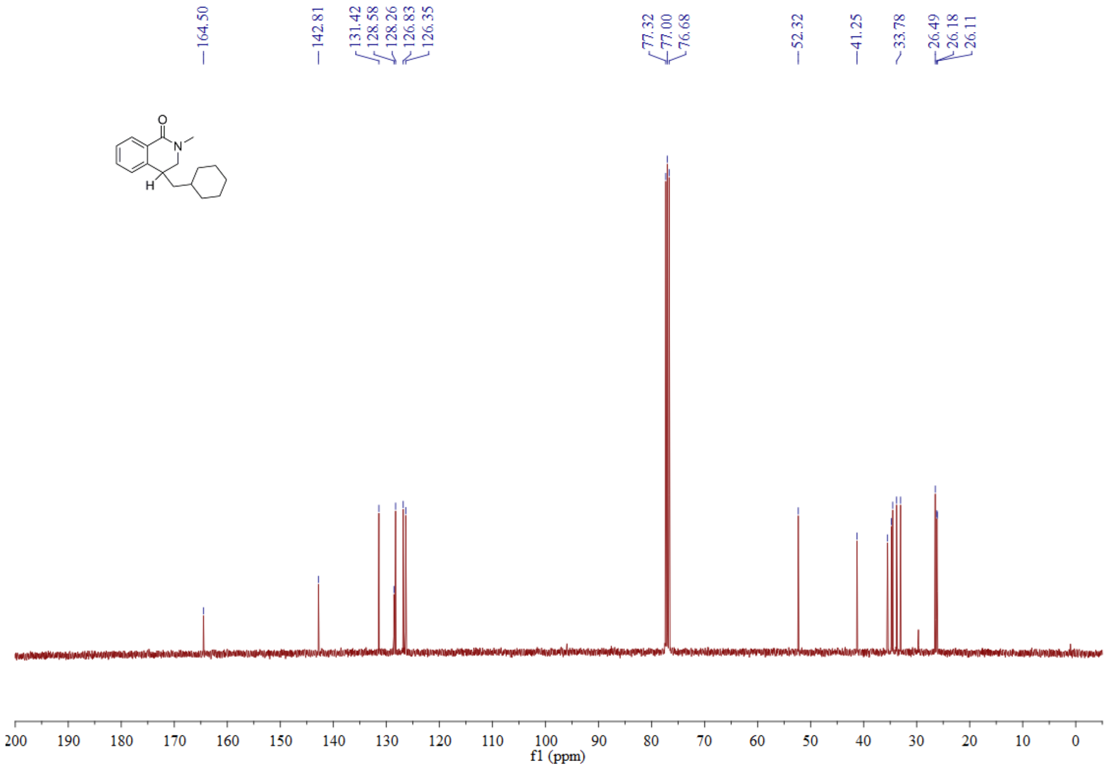

^{1}H NMR and ^{13}C NMR spectrum of **5aa**

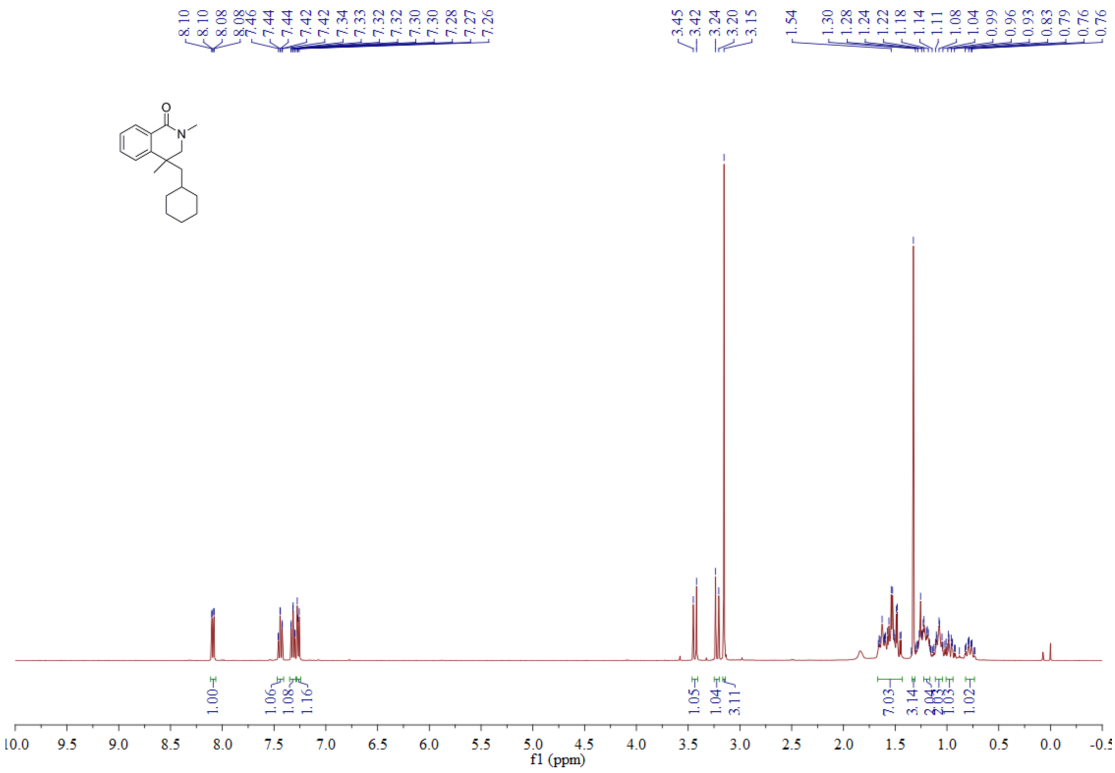

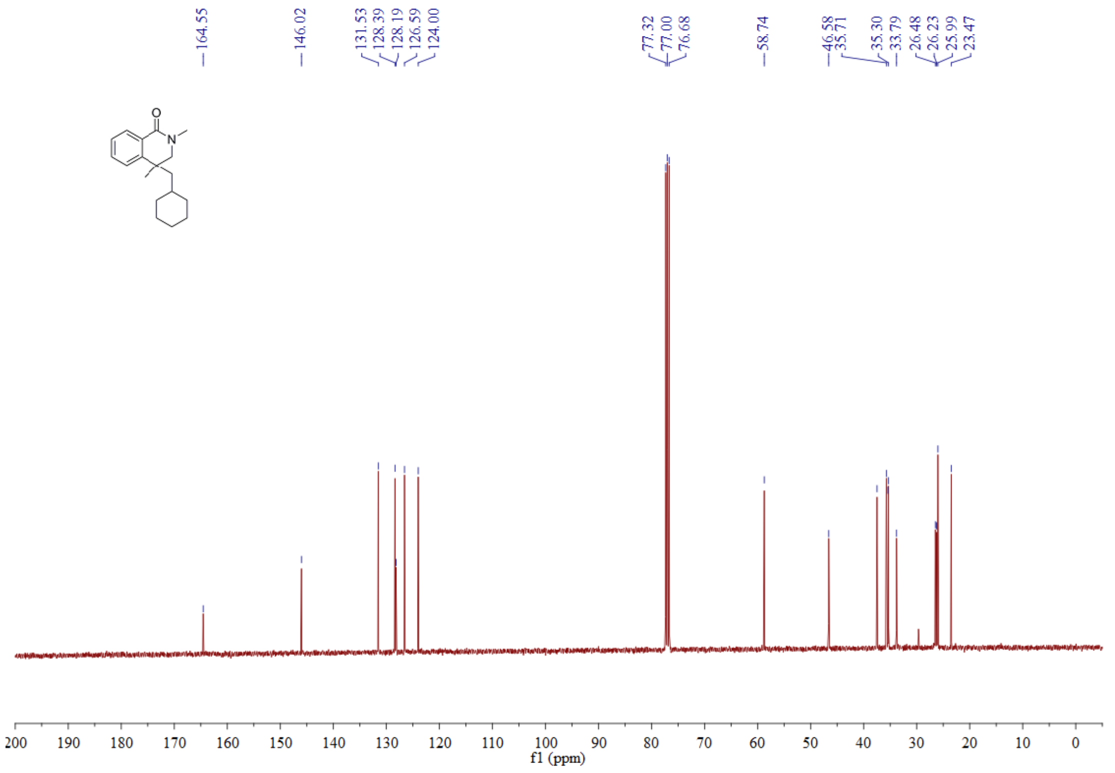

^{1}H NMR and ^{13}C NMR spectrum of **5ba**

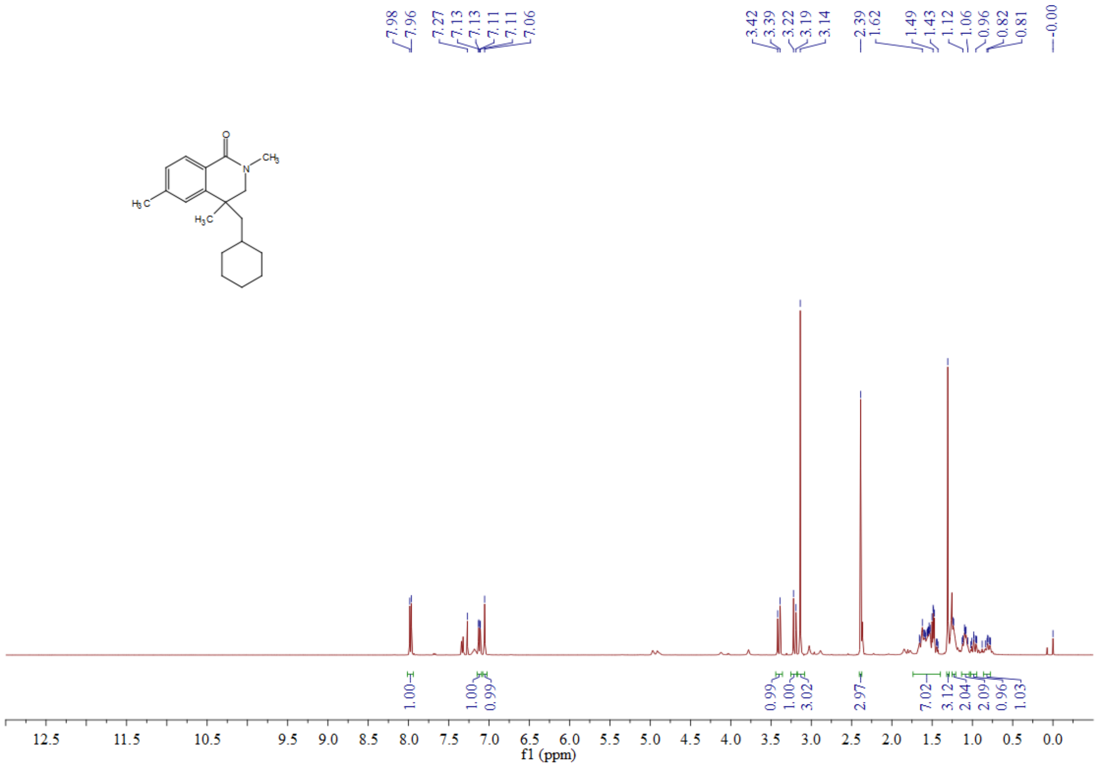

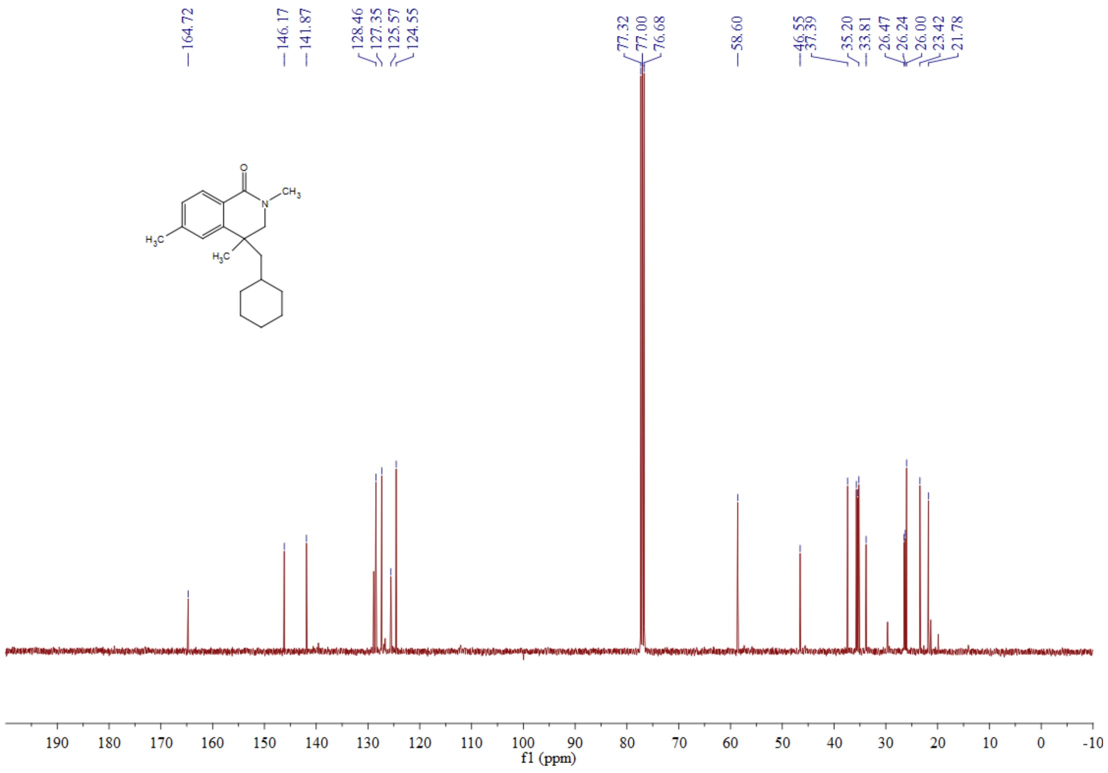

^{1}H NMR and ^{13}C NMR spectrum of **5ca**

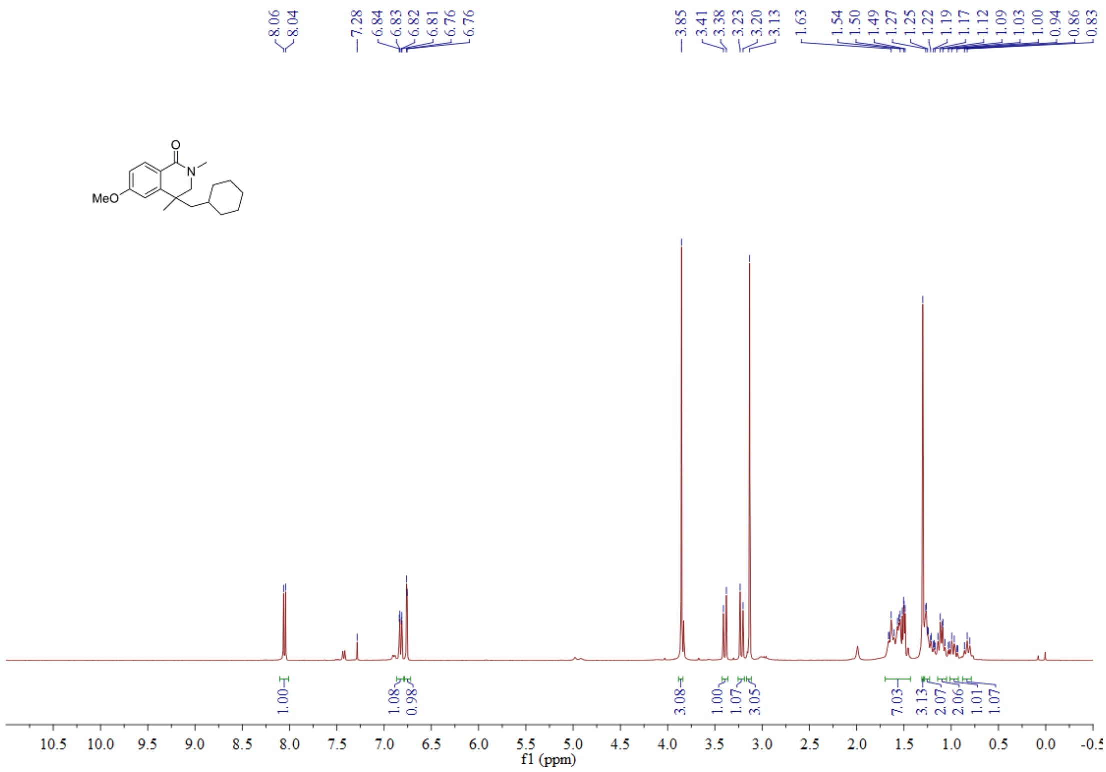

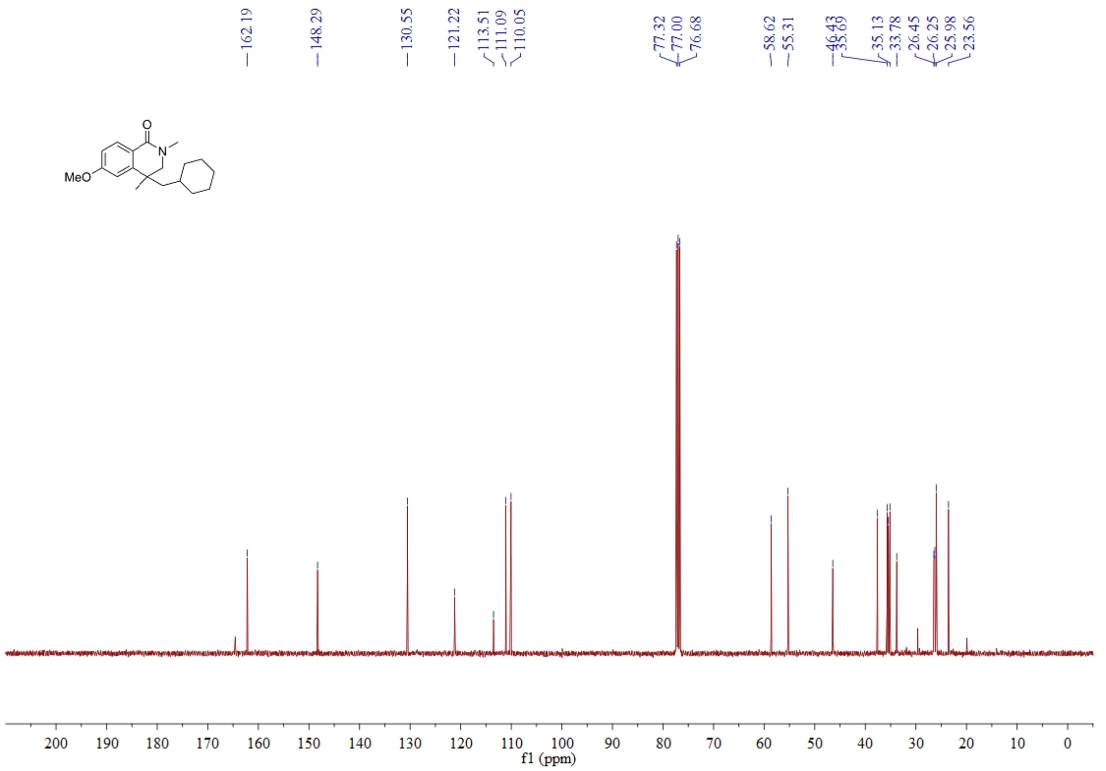

^{1}H NMR and ^{13}C NMR spectrum of **5da**

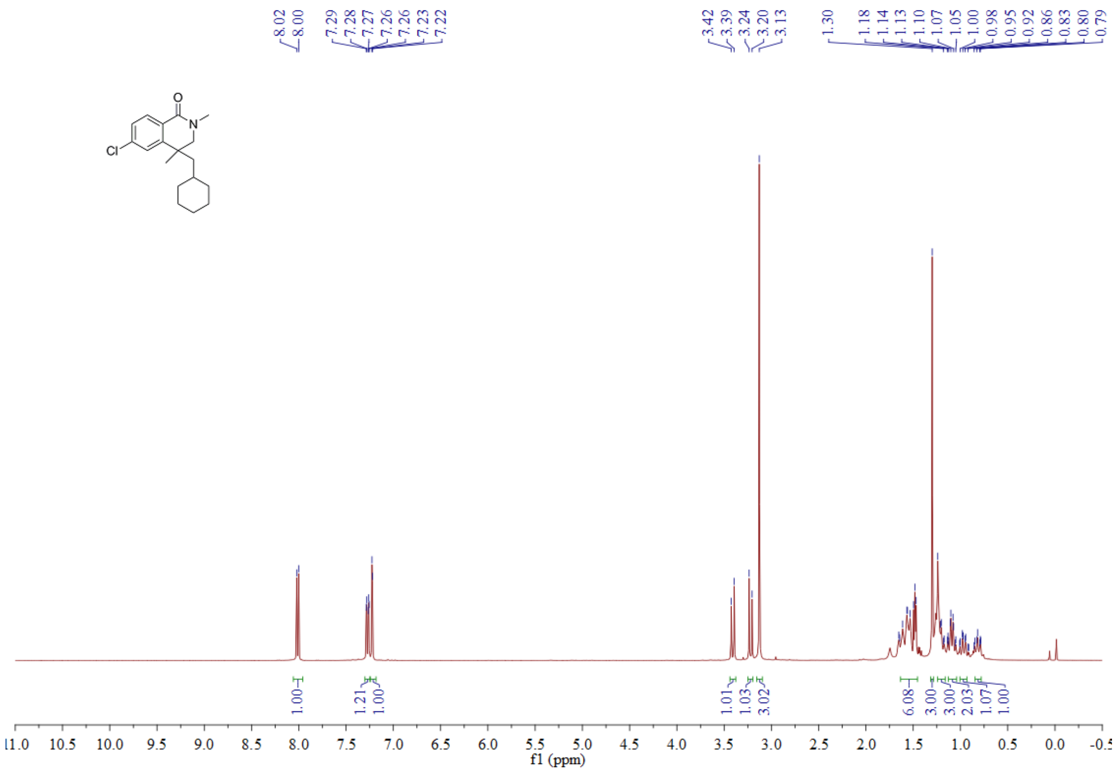

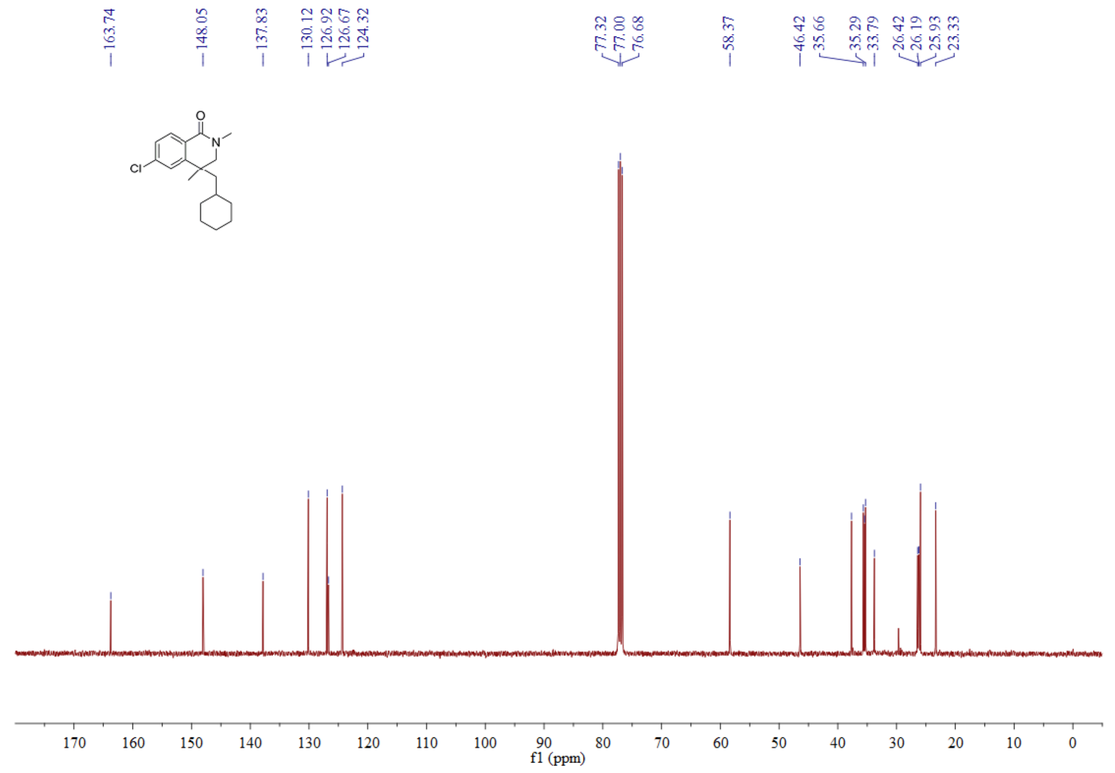

^{1}H NMR and ^{13}C NMR spectrum of **5ea**

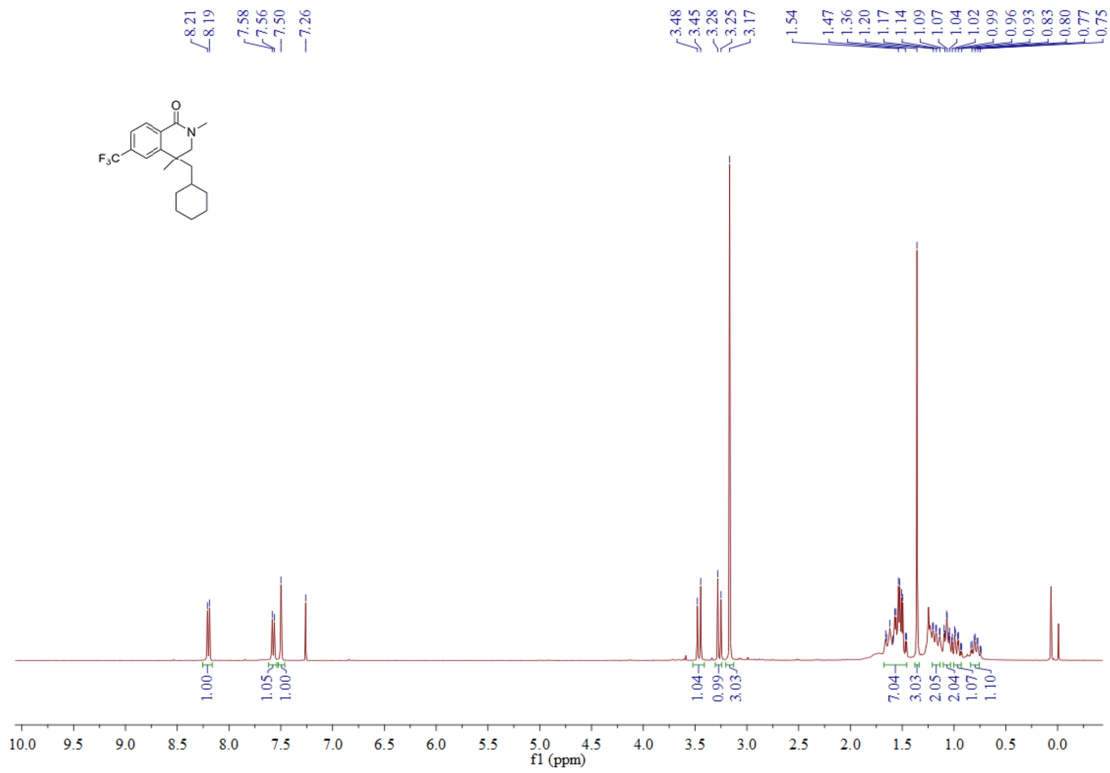

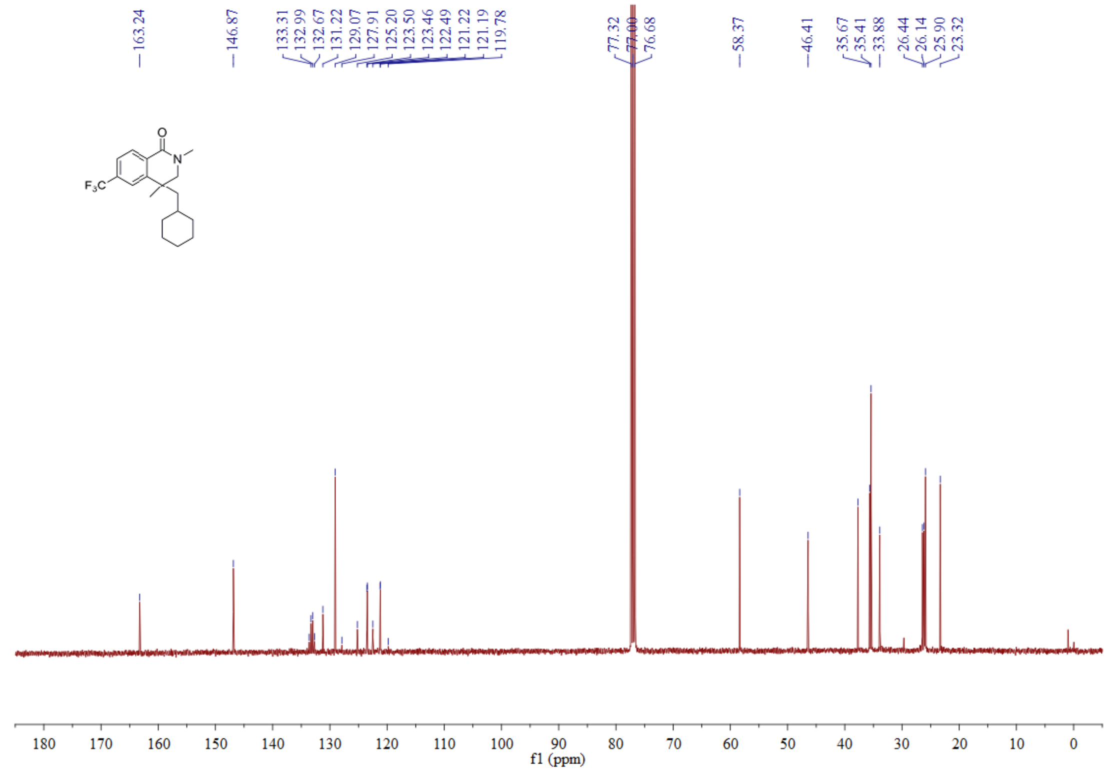

^{1}H NMR and ^{13}C NMR spectrum of **5fa**

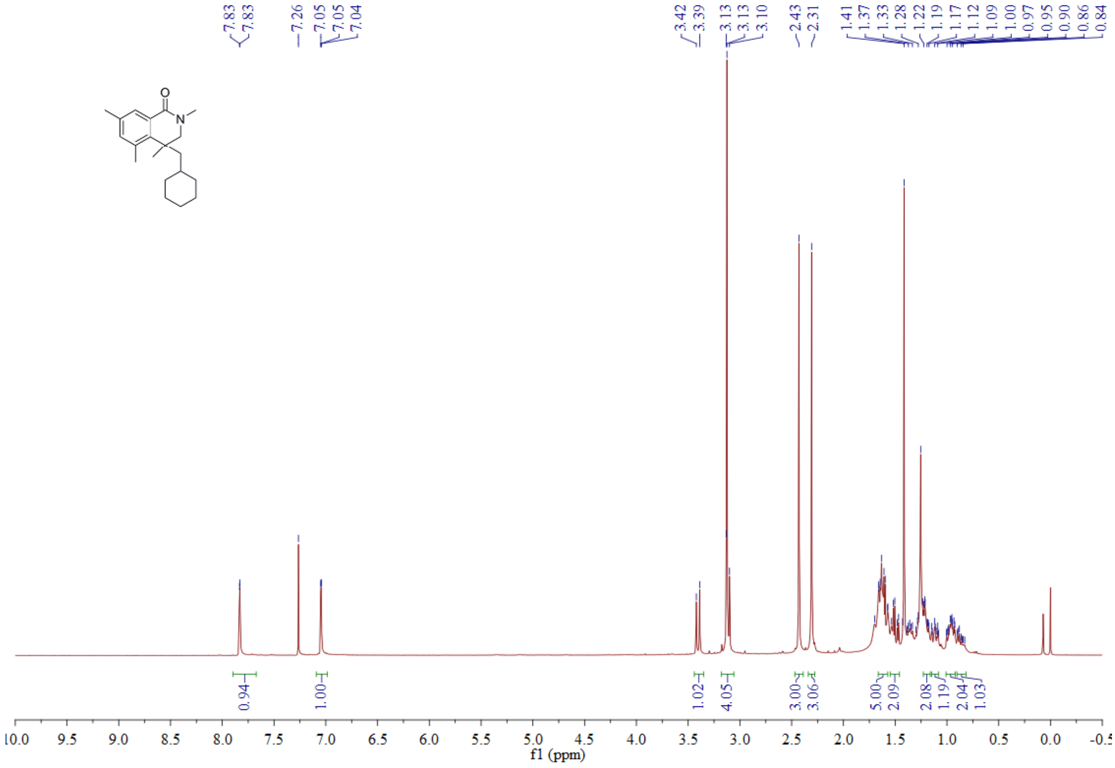

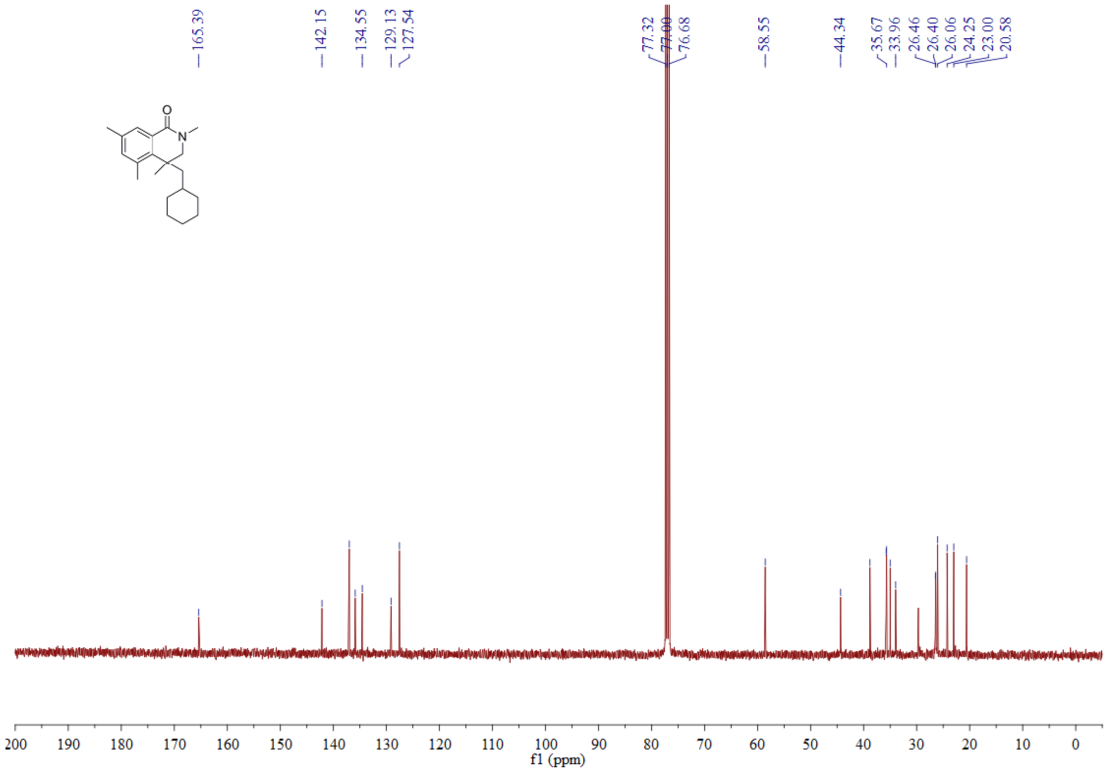

^{1}H NMR and ^{13}C NMR spectrum of **5ga**

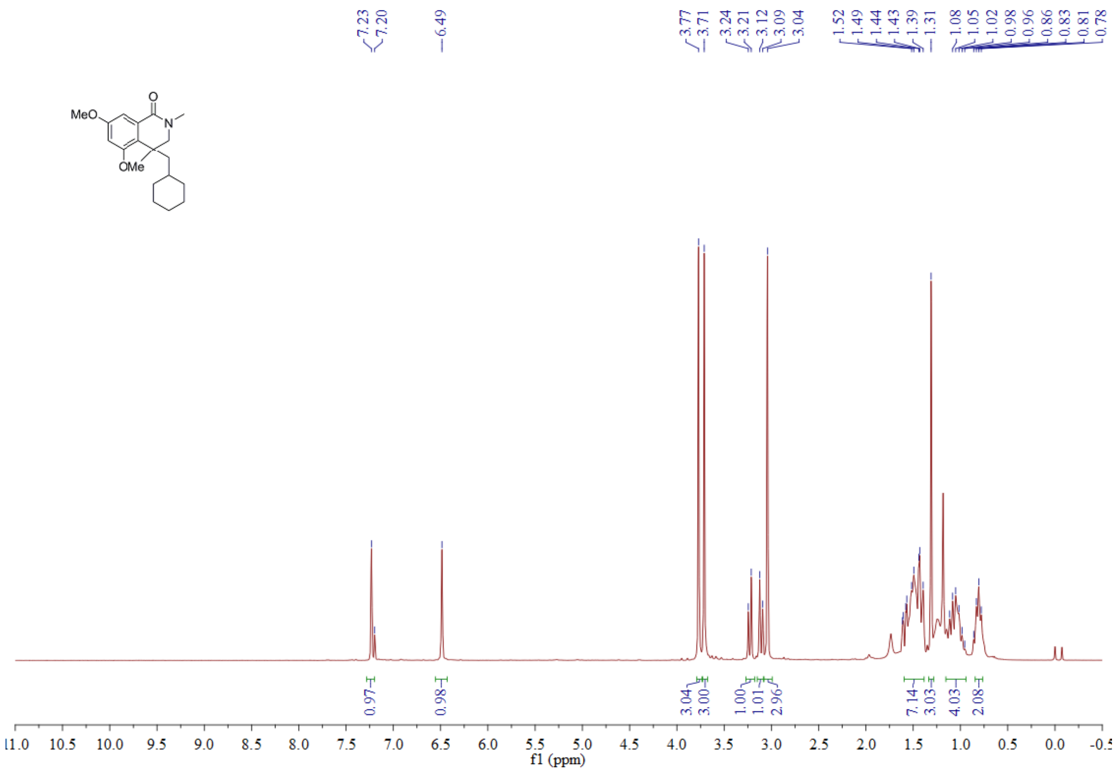

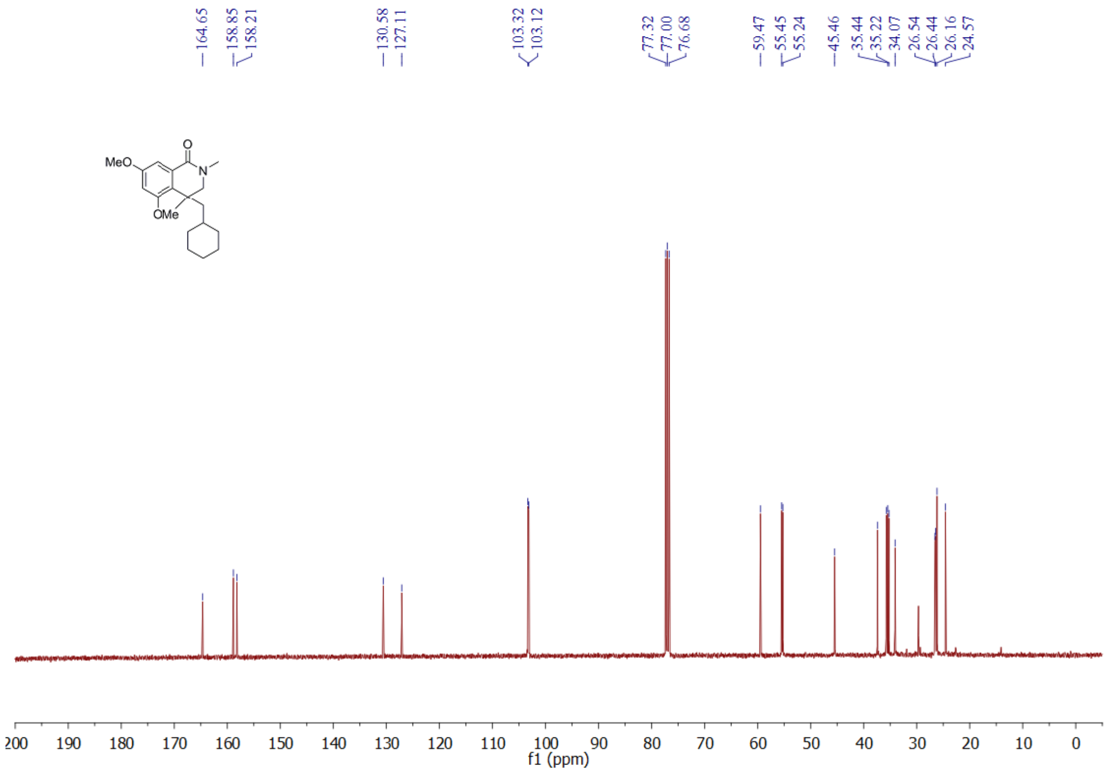

^{1}H NMR and ^{13}C NMR spectrum of **5ha**

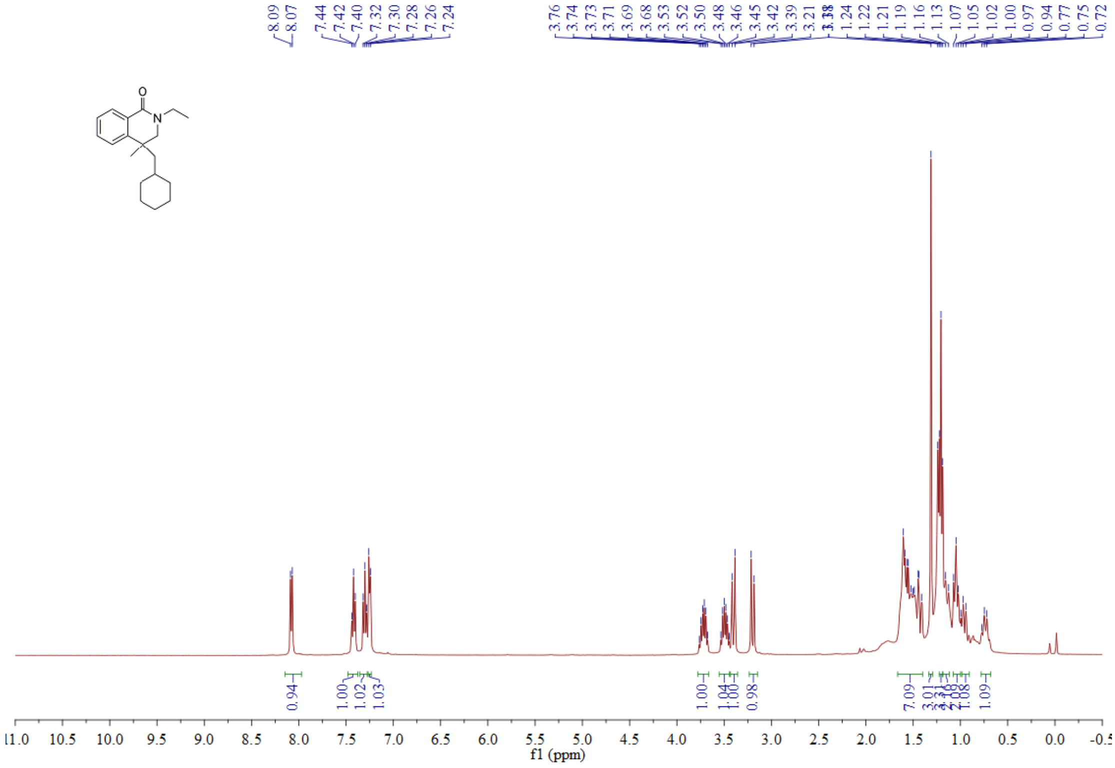

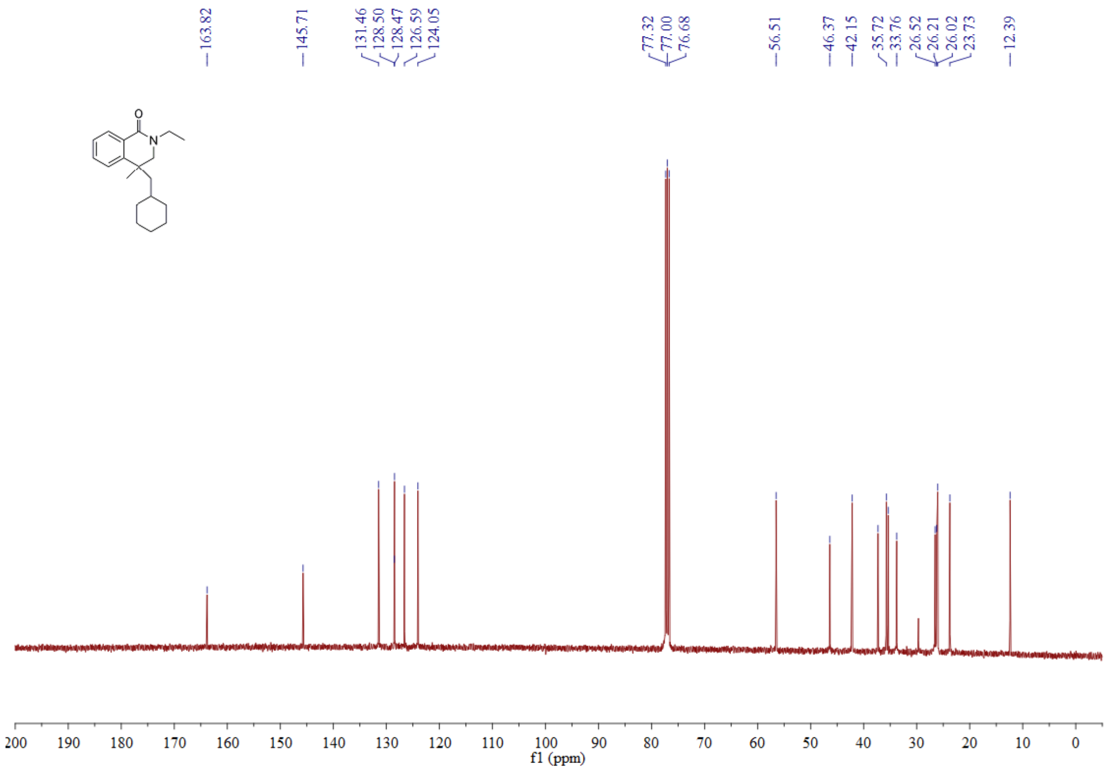

^{1}H NMR and ^{13}C NMR spectrum of **5ia**

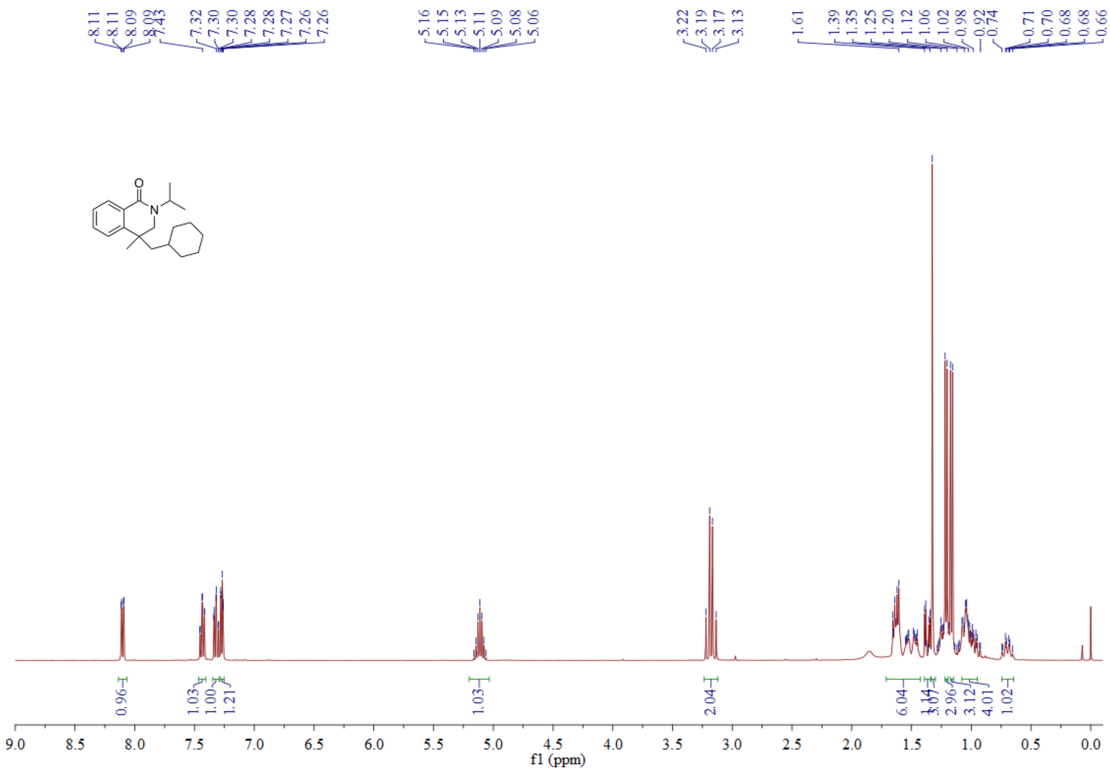

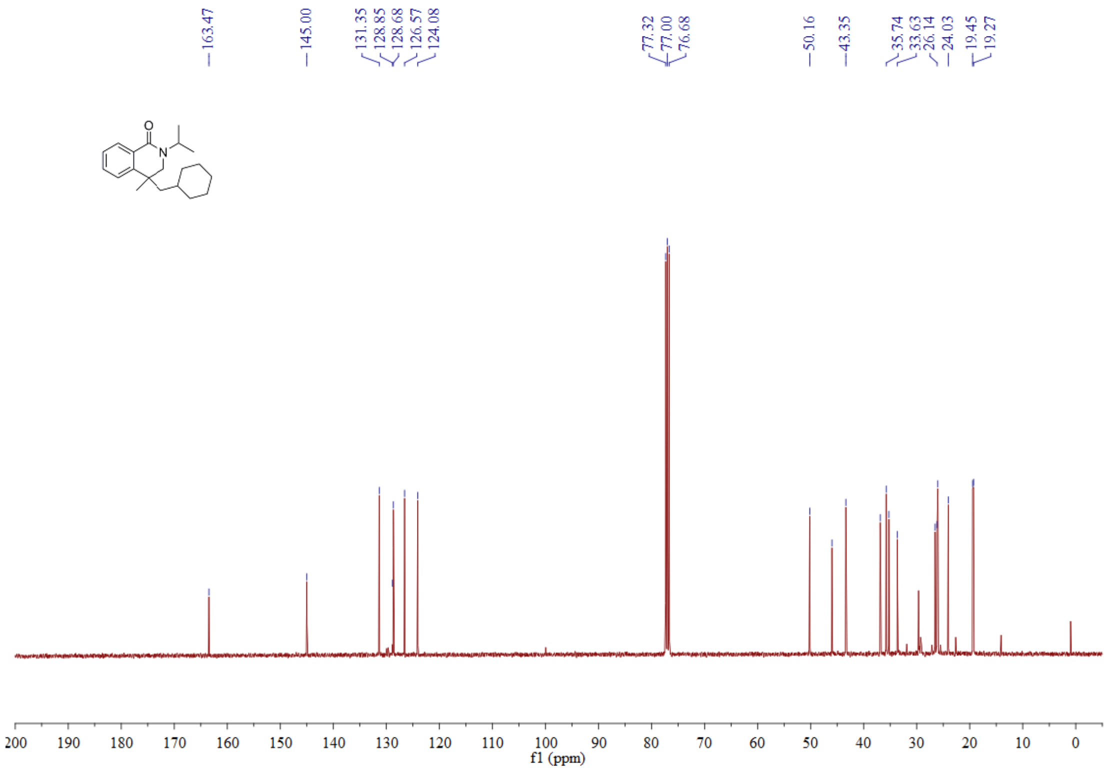

^{1}H NMR and ^{13}C NMR spectrum of **5ja**

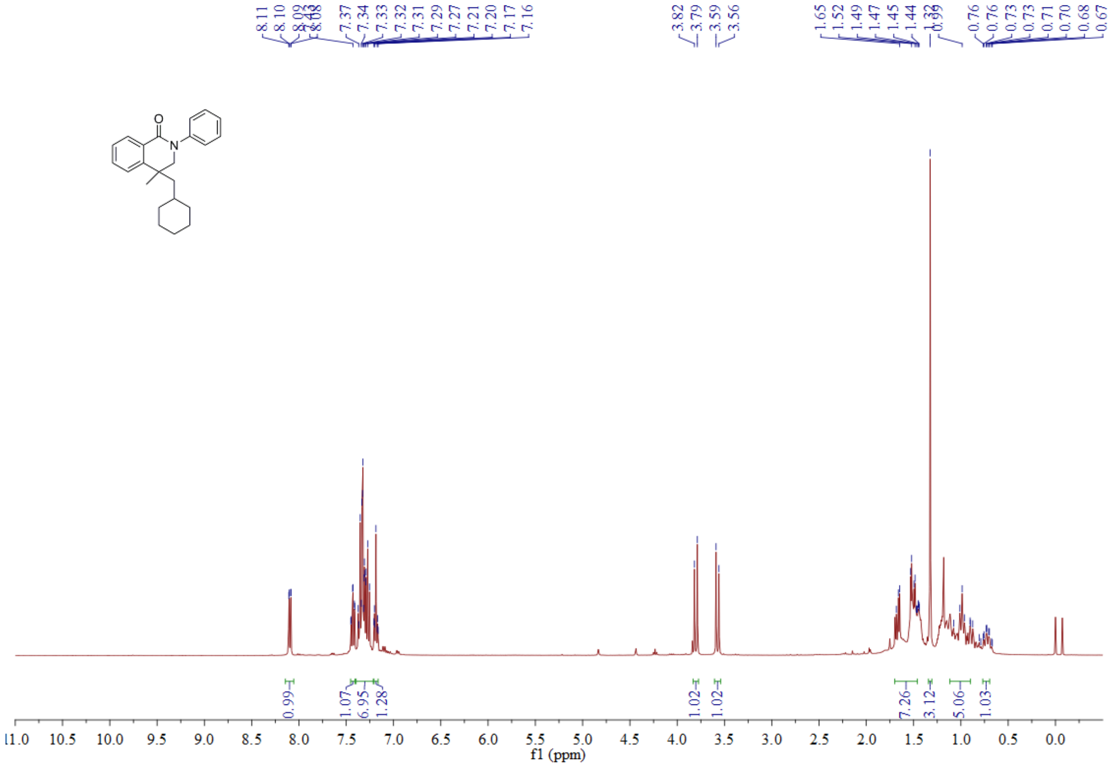

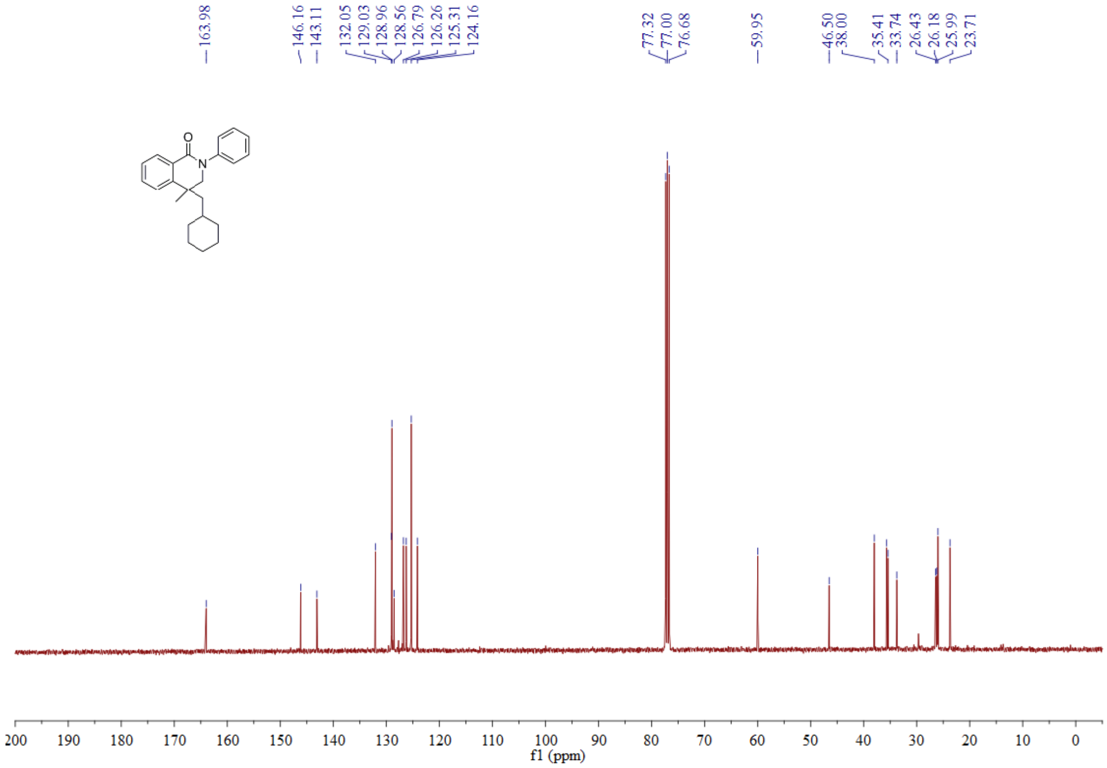

^{1}H NMR and ^{13}C NMR spectrum of **5ka**

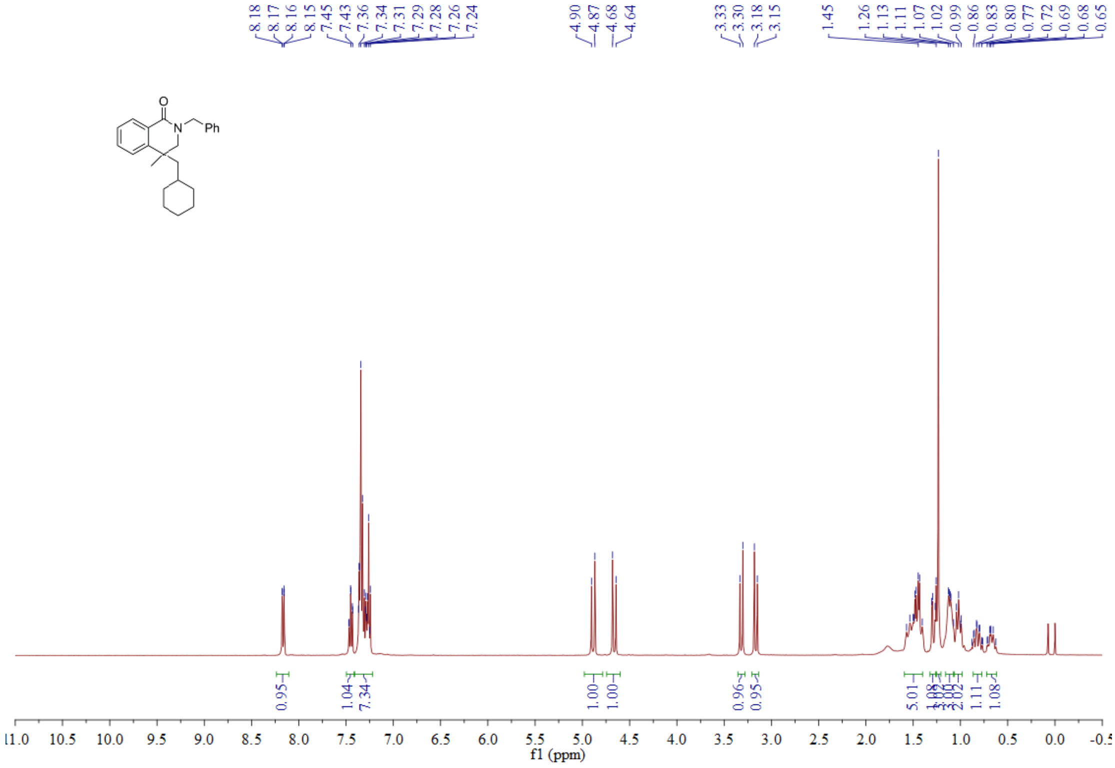

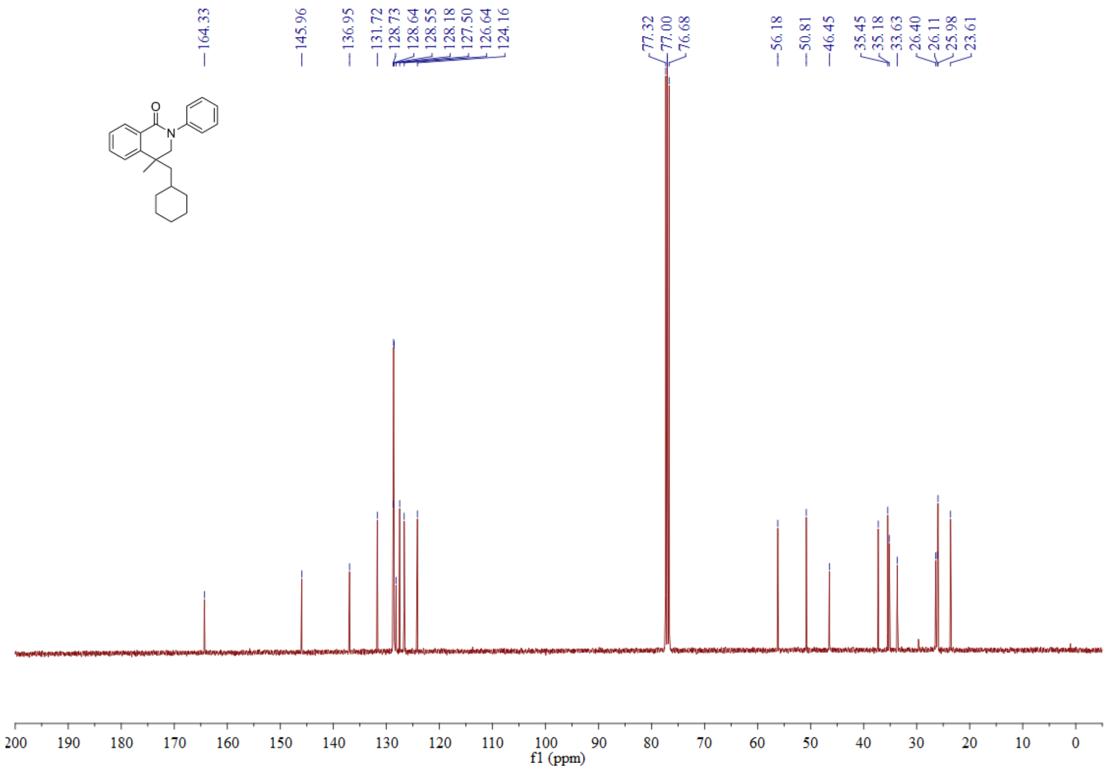

^{1}H NMR and ^{13}C NMR spectrum of **5ab**

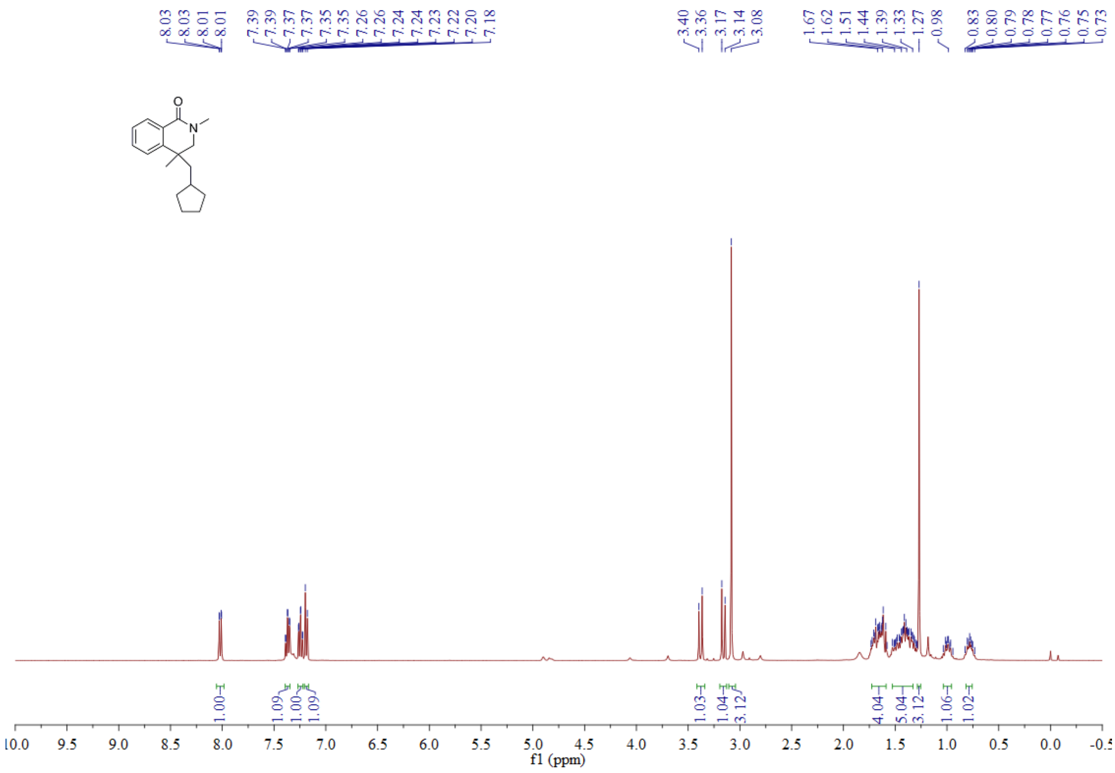

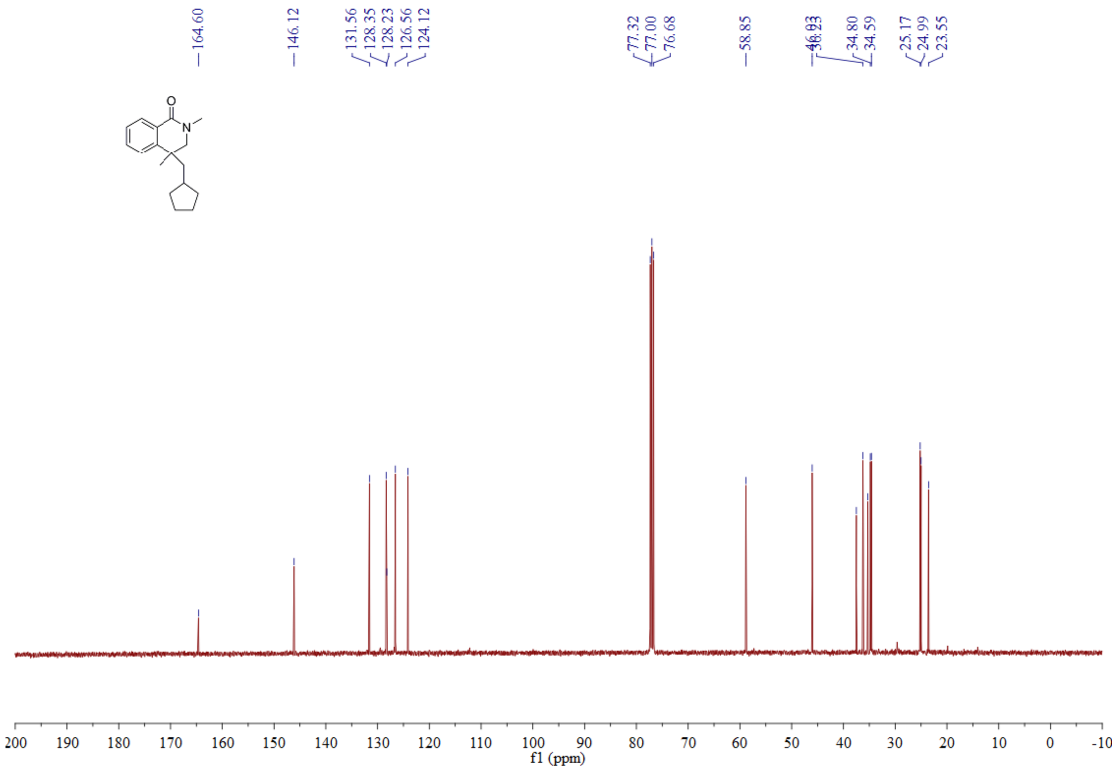

^{1}H NMR and ^{13}C NMR spectrum of **5ac**

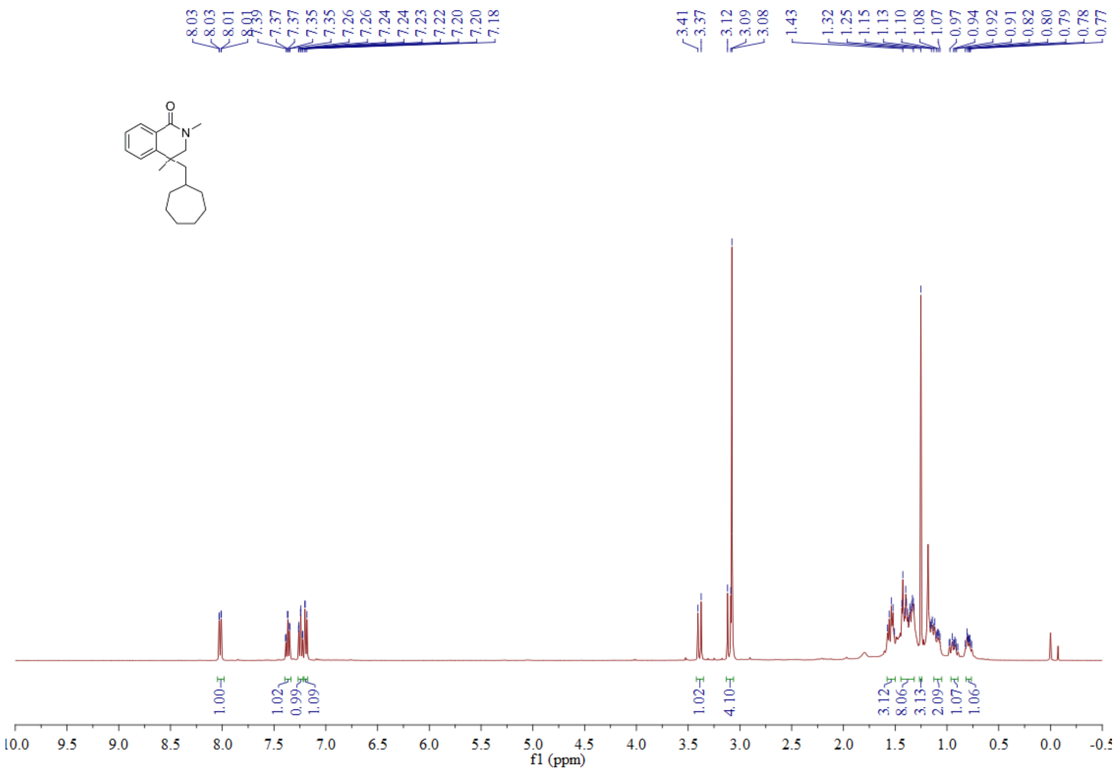

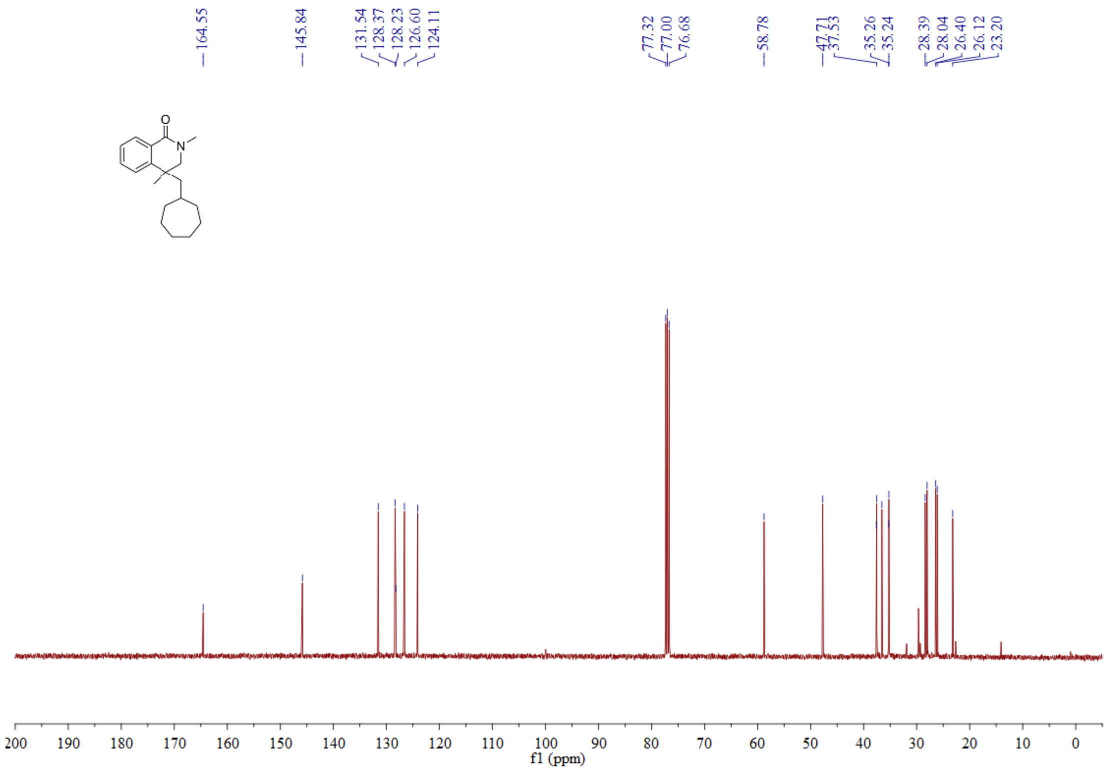

^{1}H NMR and ^{13}C NMR spectrum of **5ad**

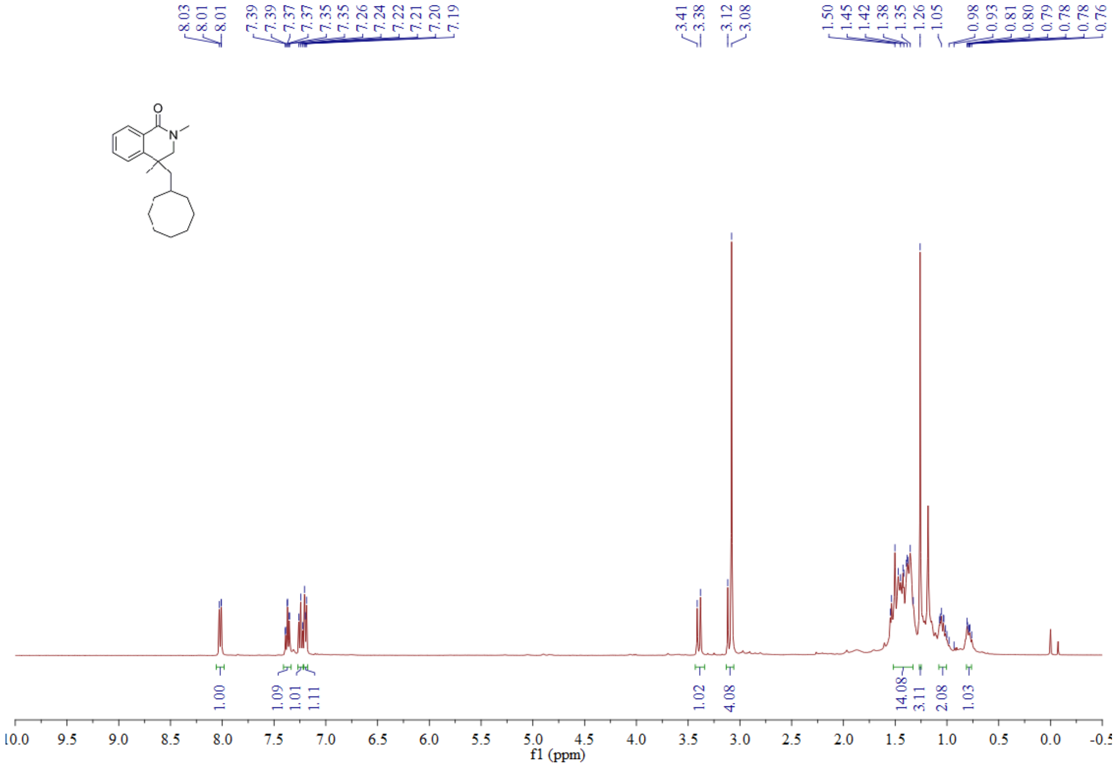

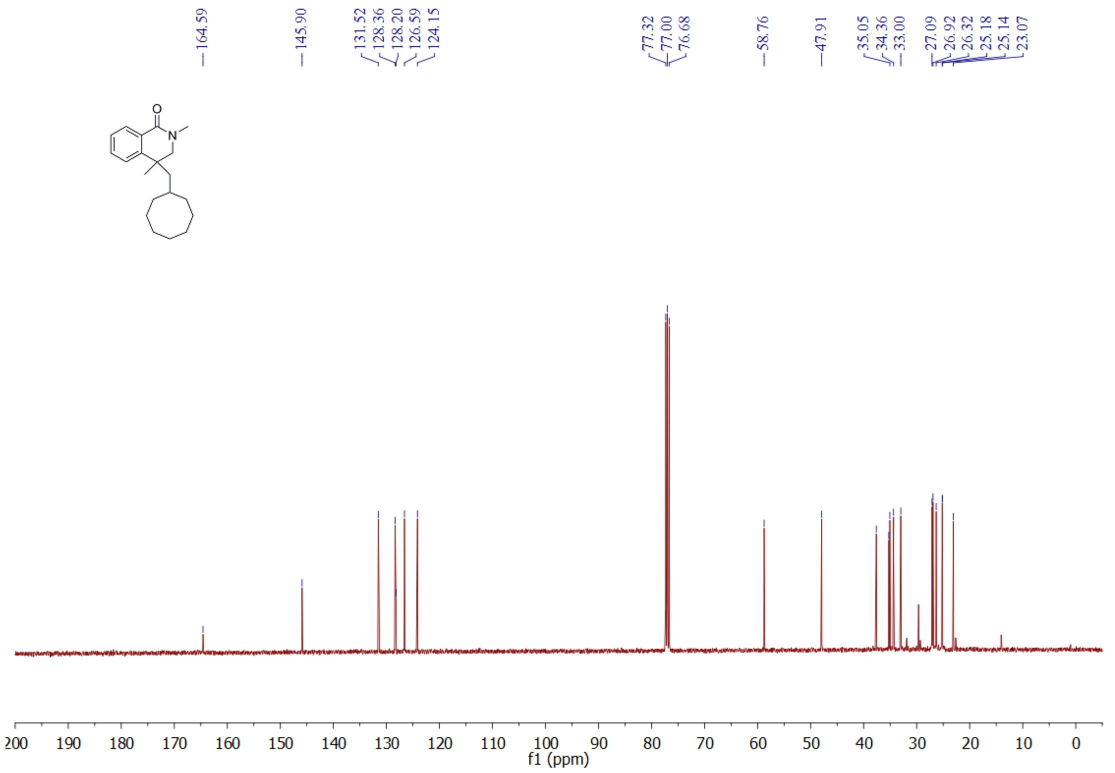

^{1}H NMR and ^{13}C NMR spectrum of **5ae1-5ae**

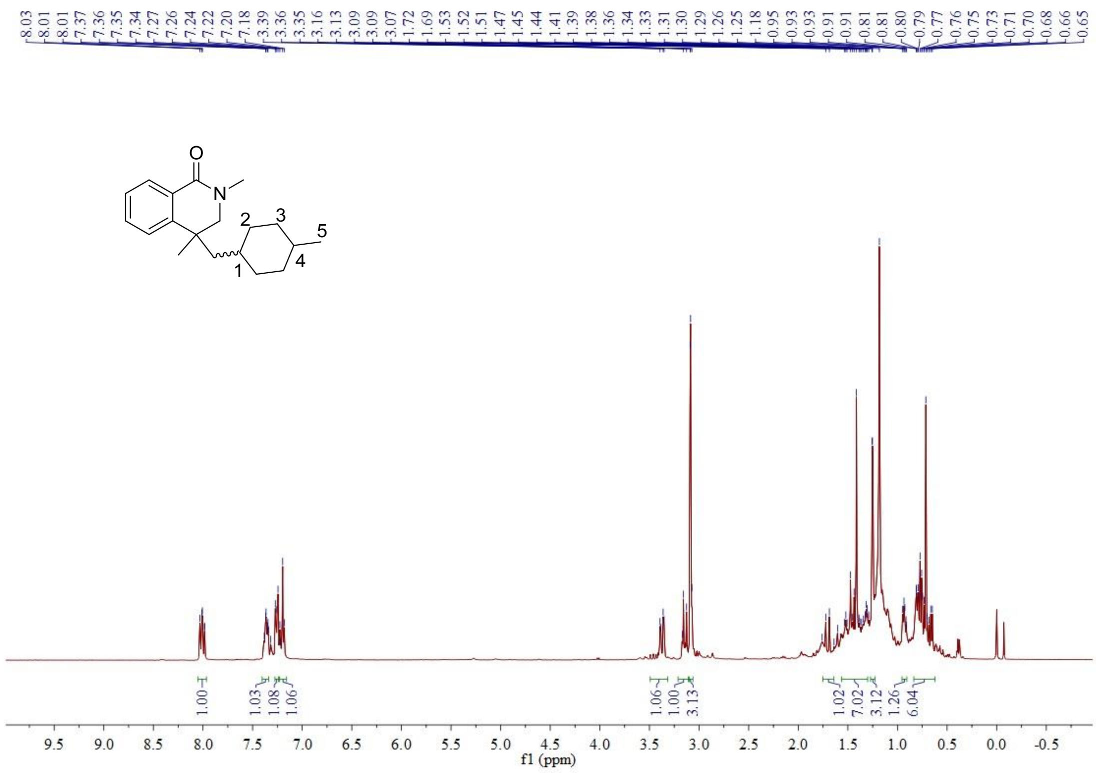

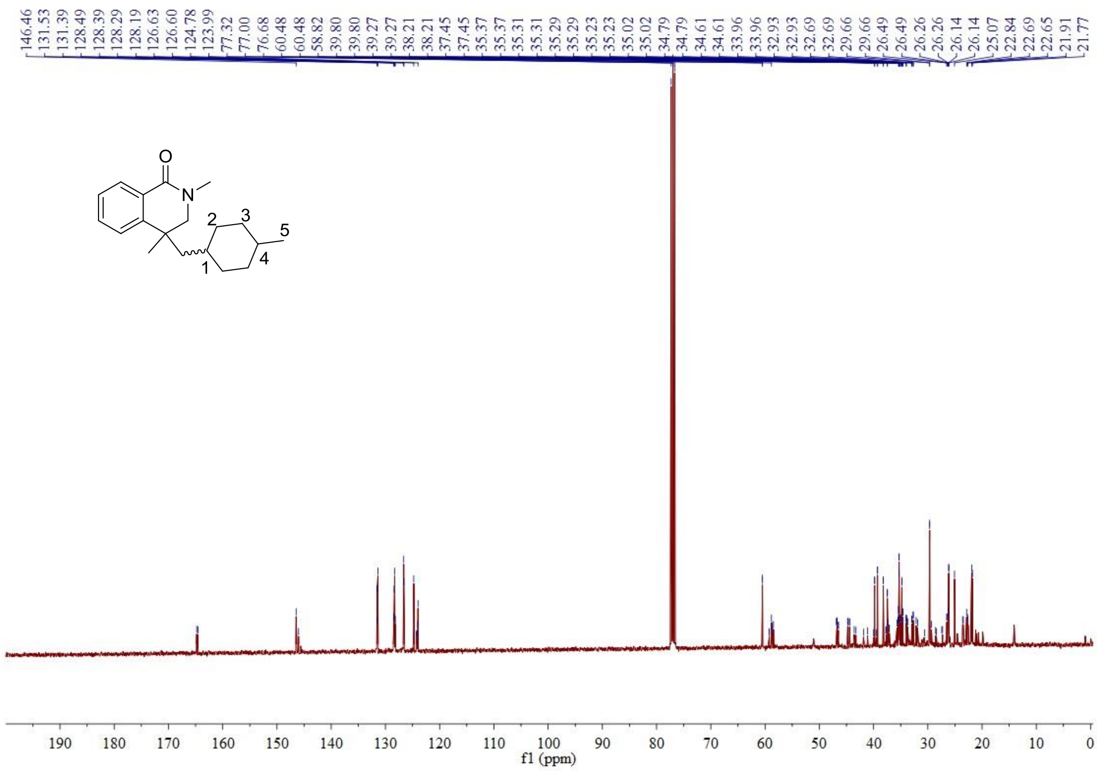

^{1}H NMR and ^{13}C NMR spectrum of **7aa**

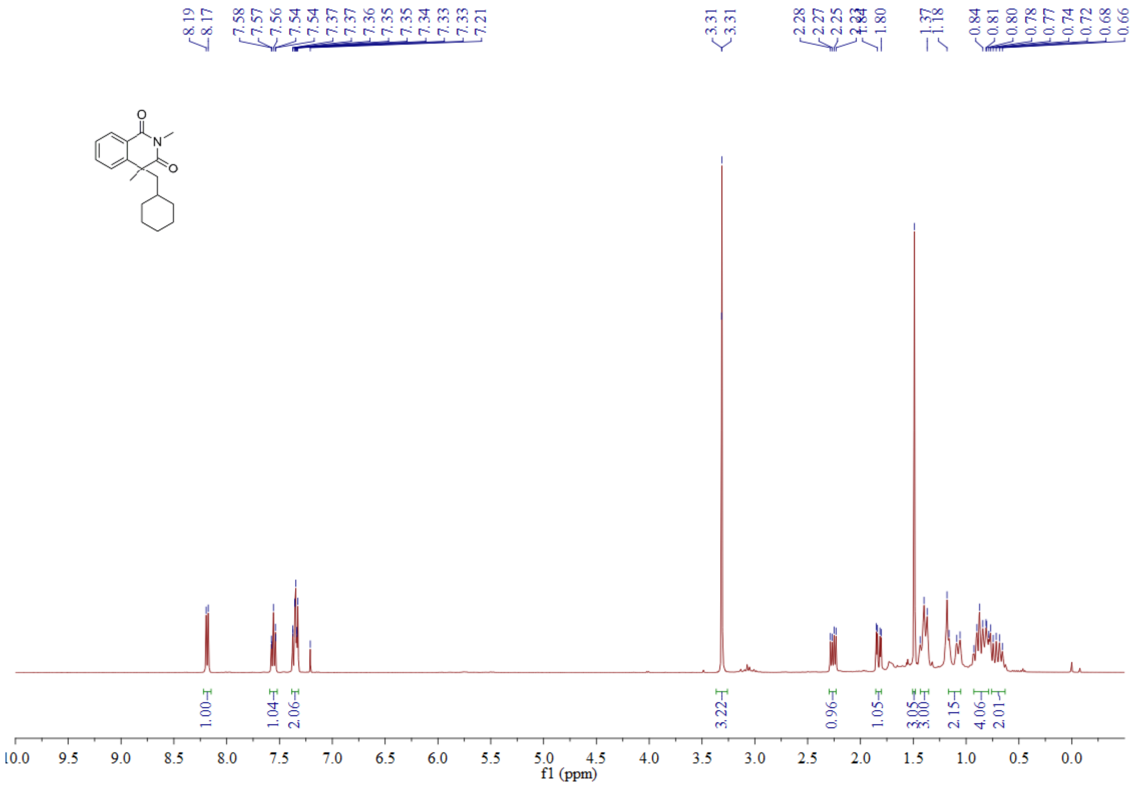

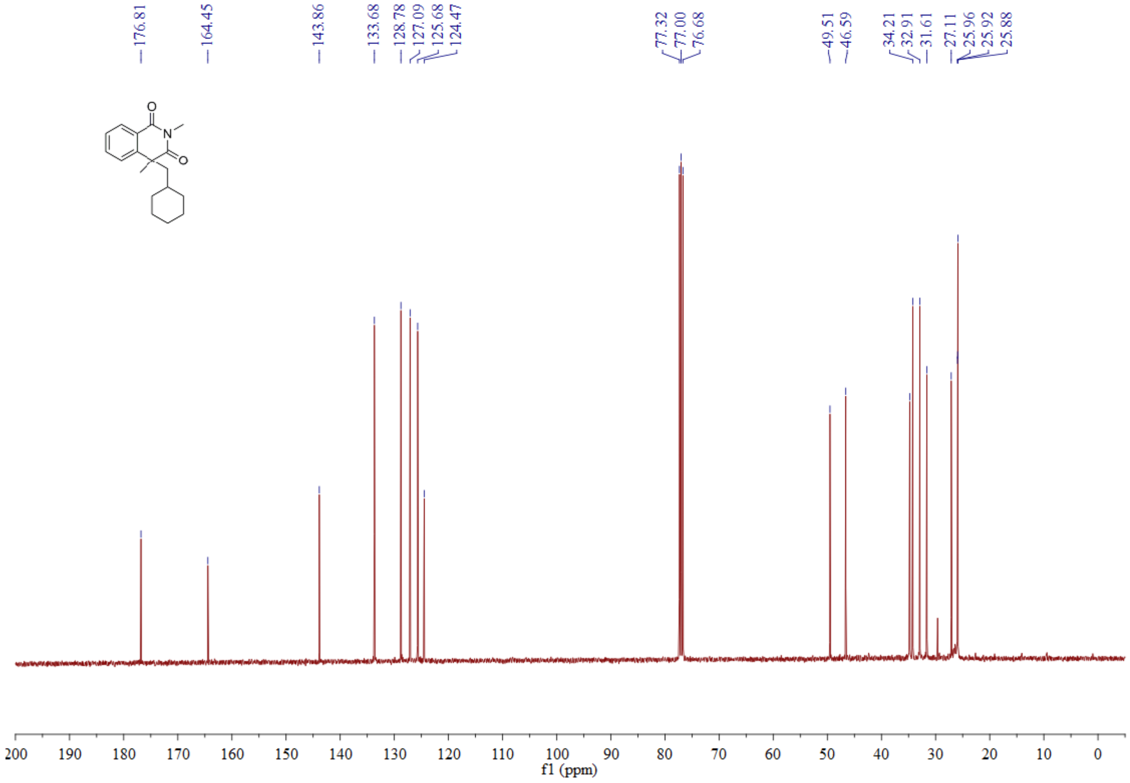

^{1}H NMR and ^{13}C NMR spectrum of **7ba**

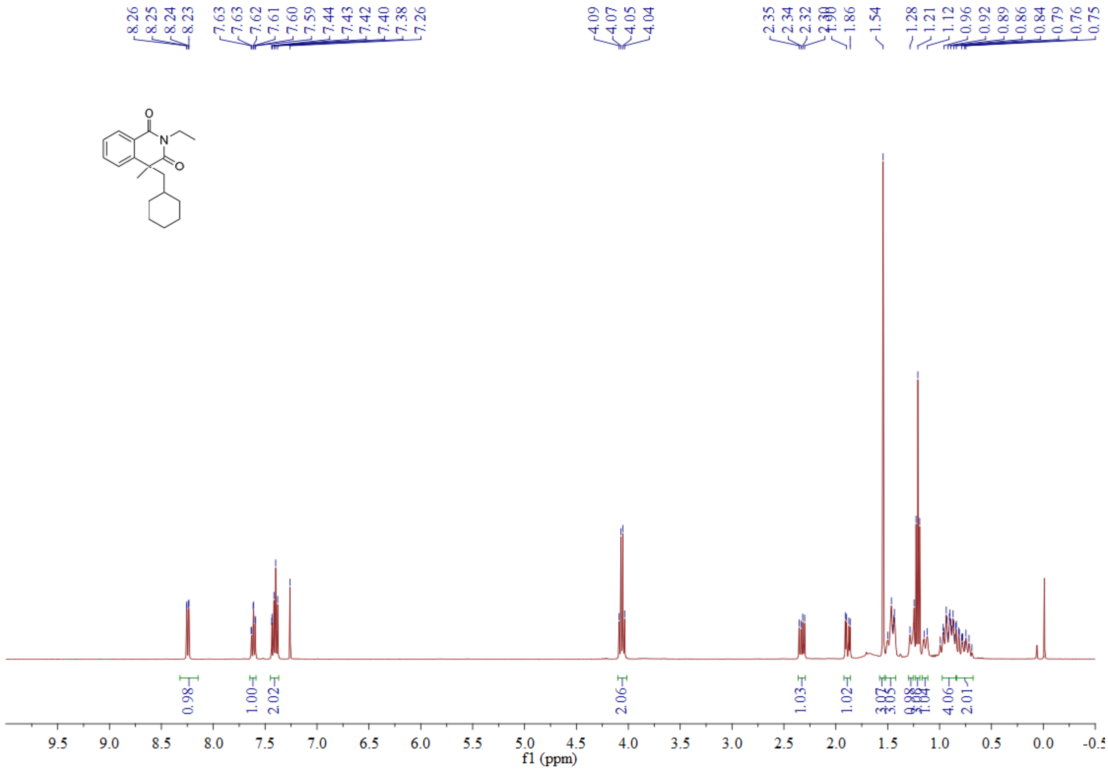

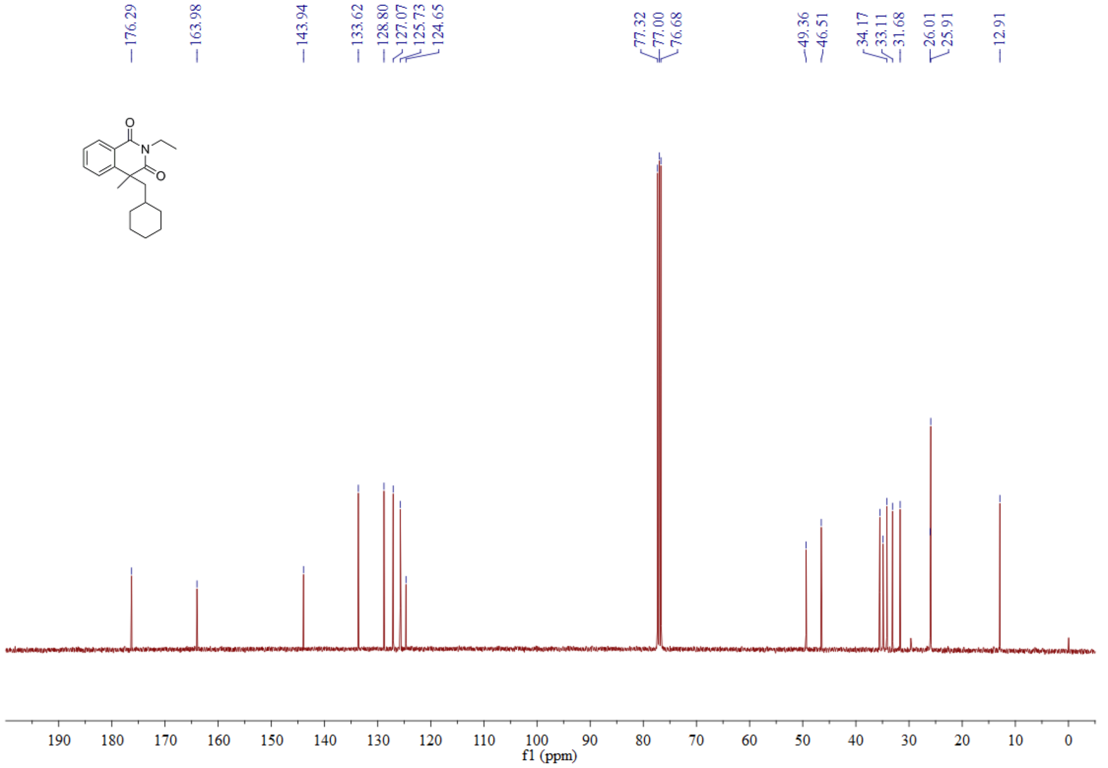

^{1}H NMR and ^{13}C NMR spectrum of **7ca**

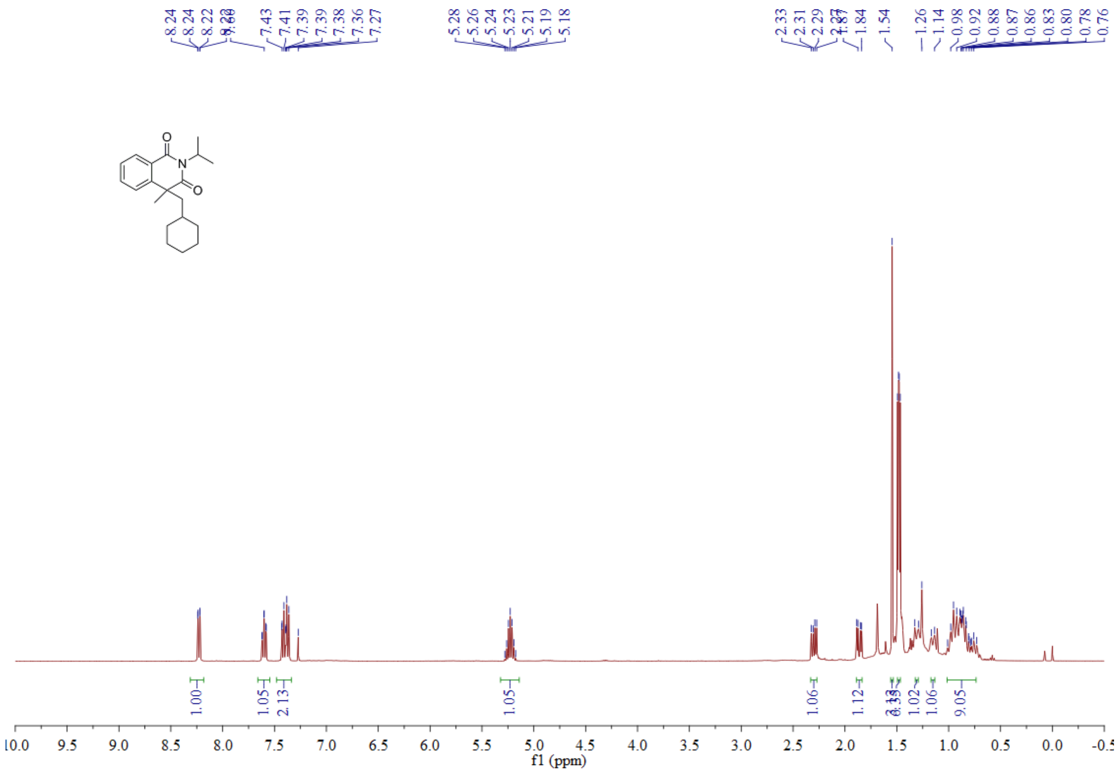

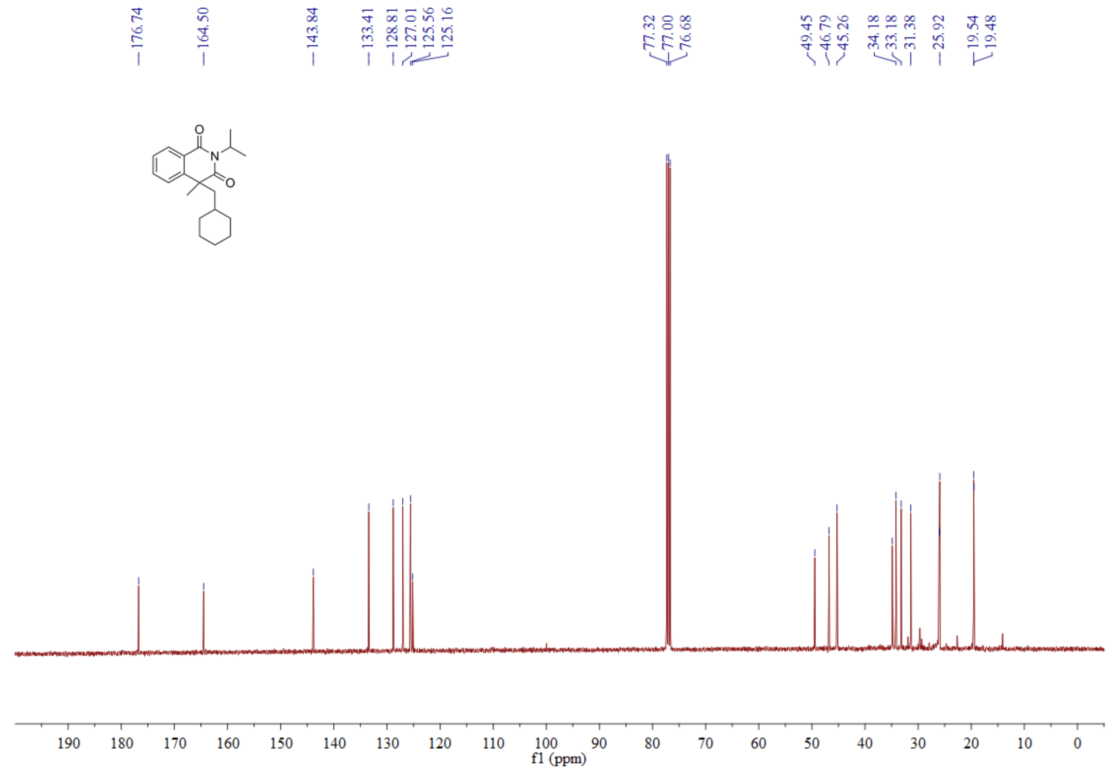

^{1}H NMR and ^{13}C NMR spectrum of **7da**

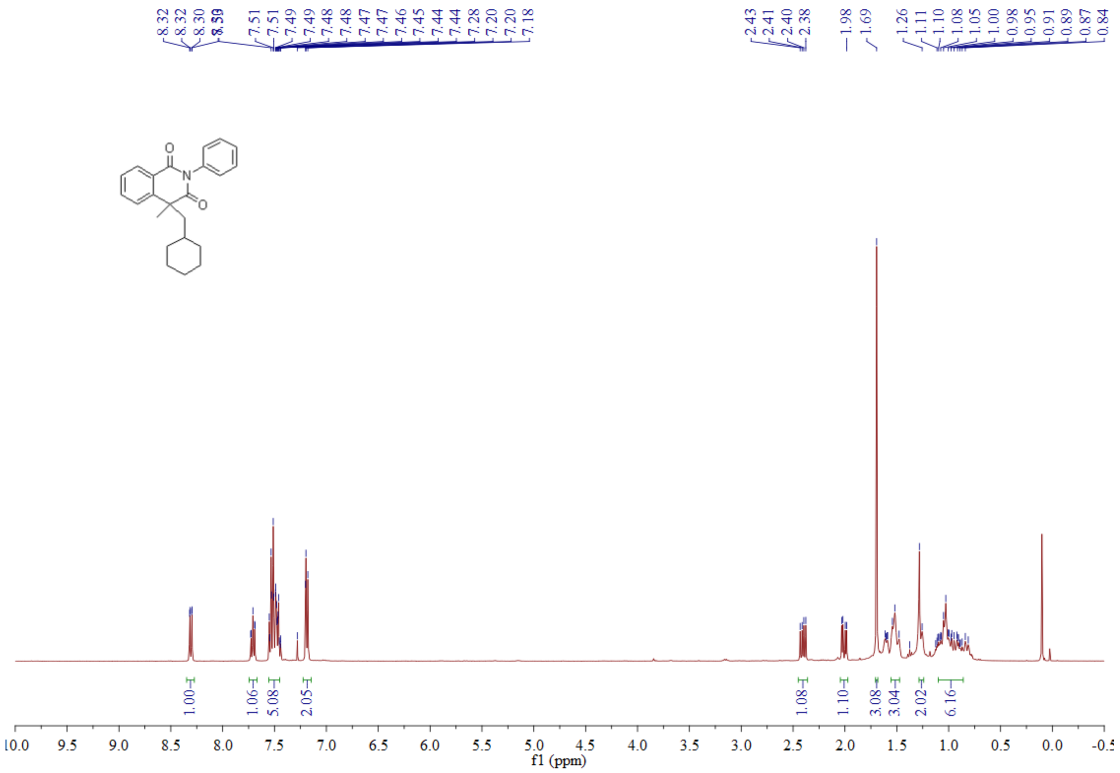

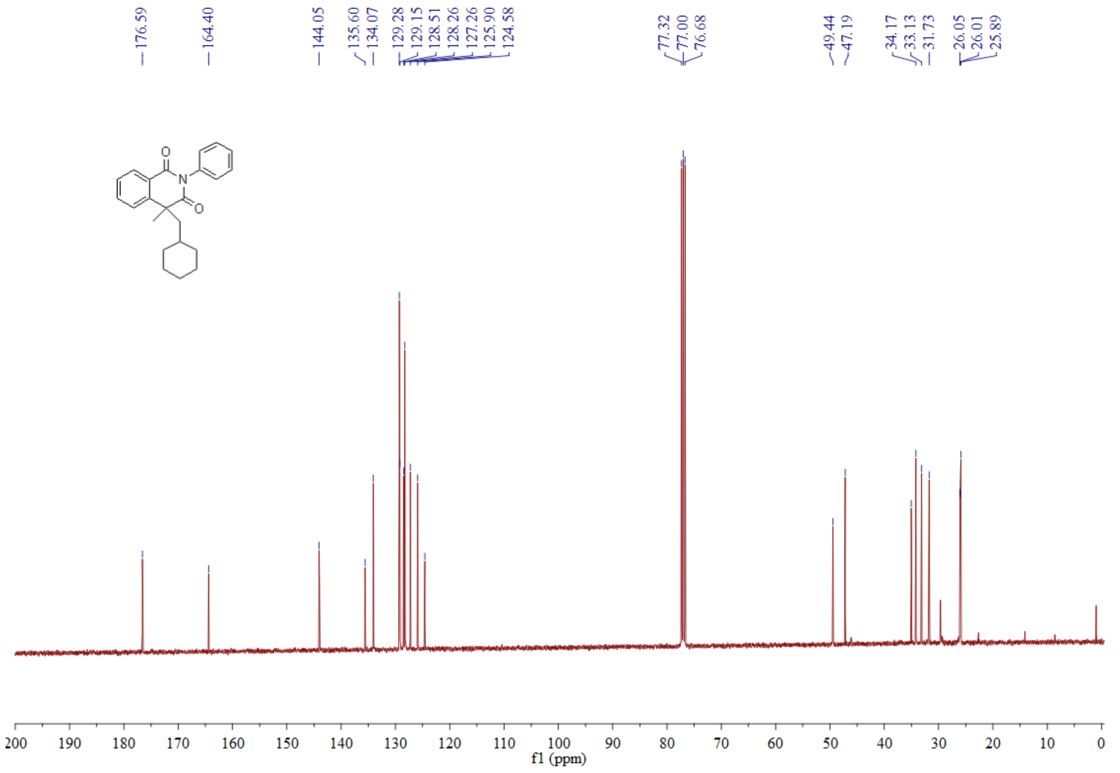

^{1}H NMR and ^{13}C NMR spectrum of **7ea**

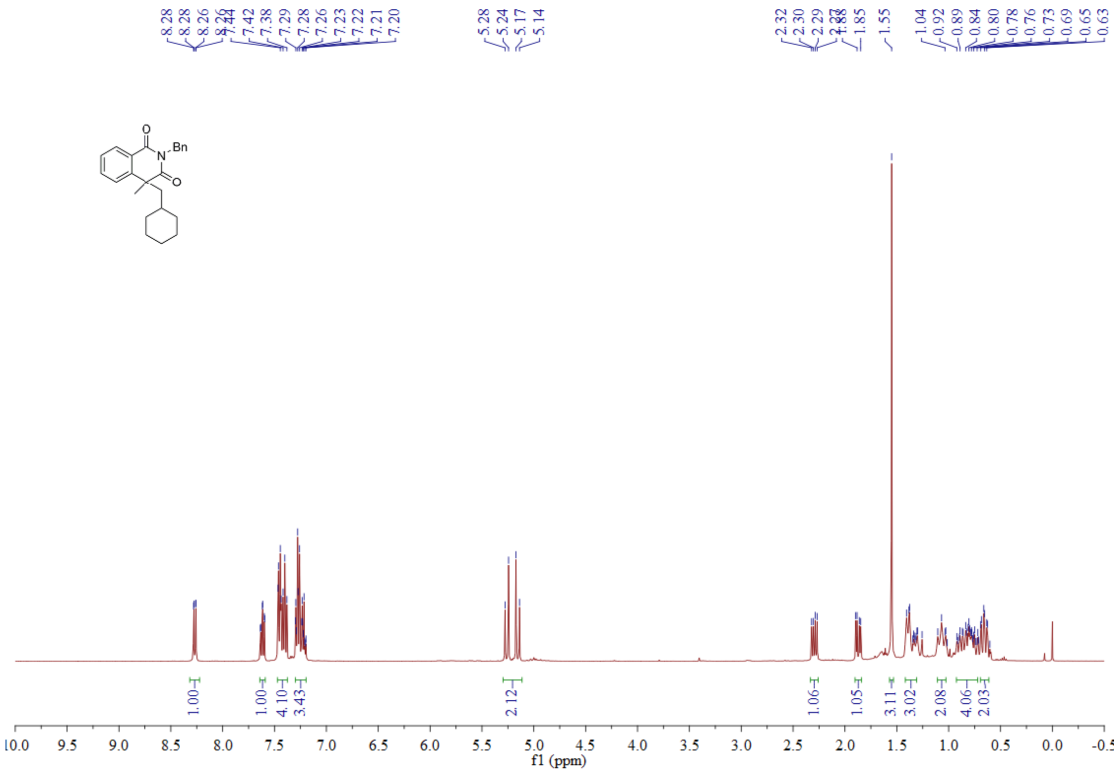

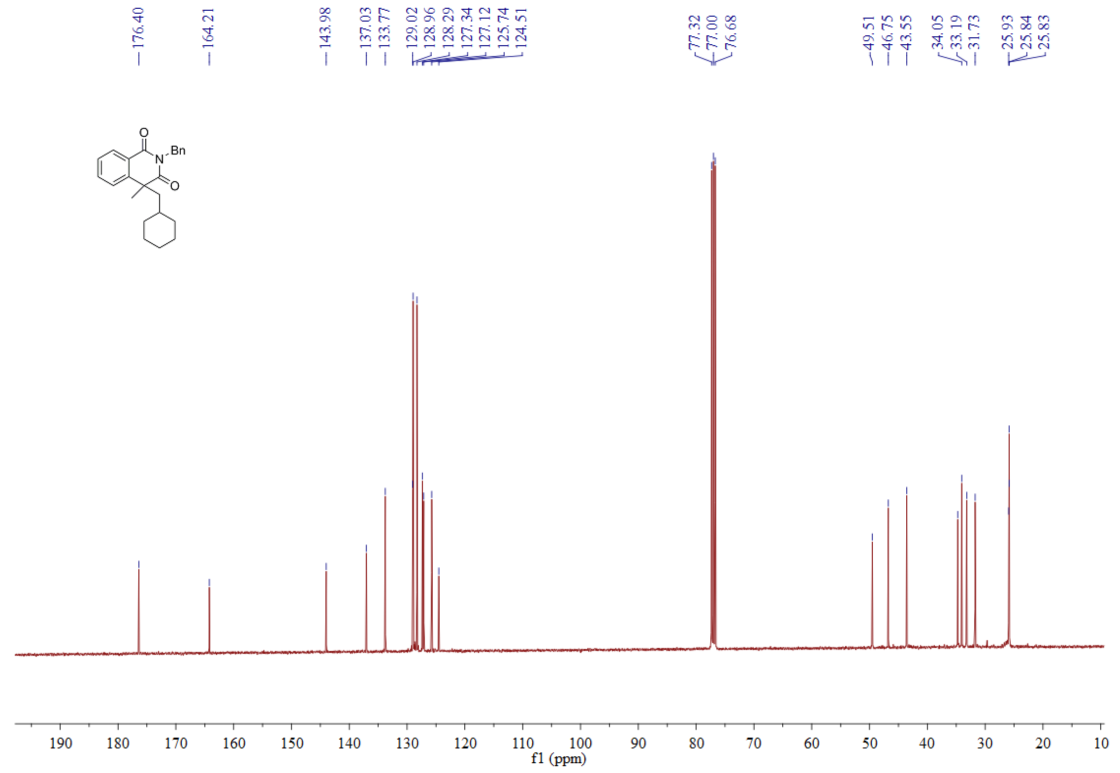

^{1}H NMR and ^{13}C NMR spectrum of **7fa**

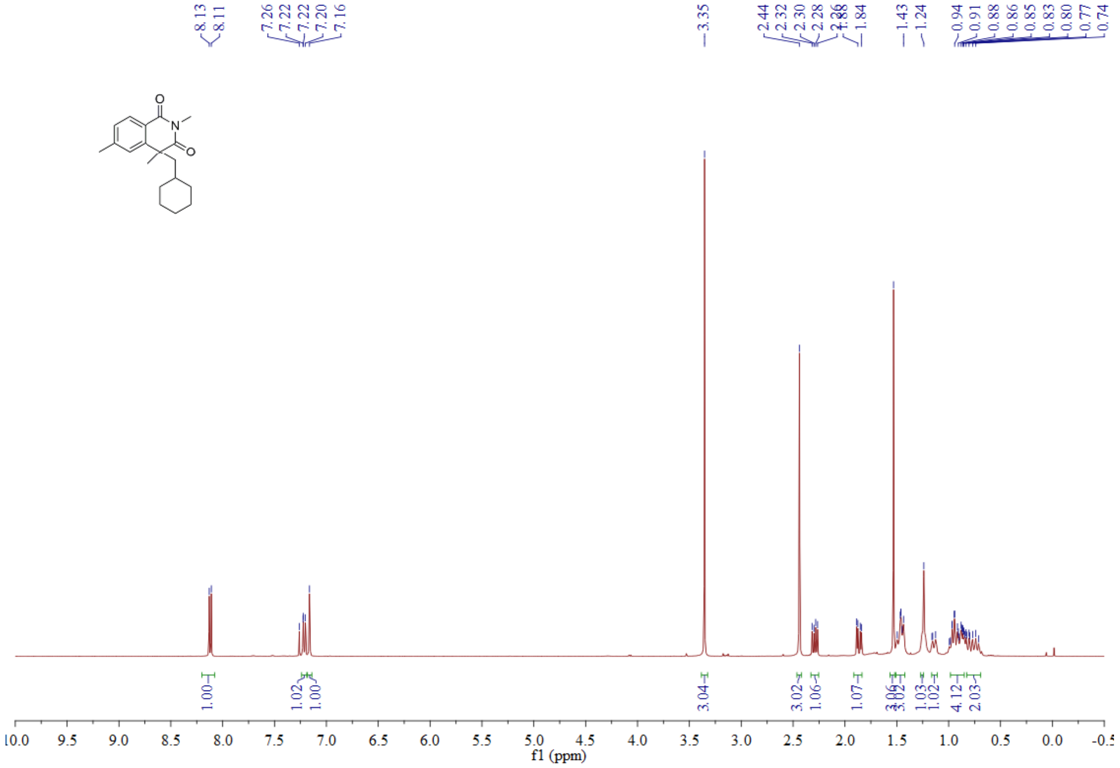

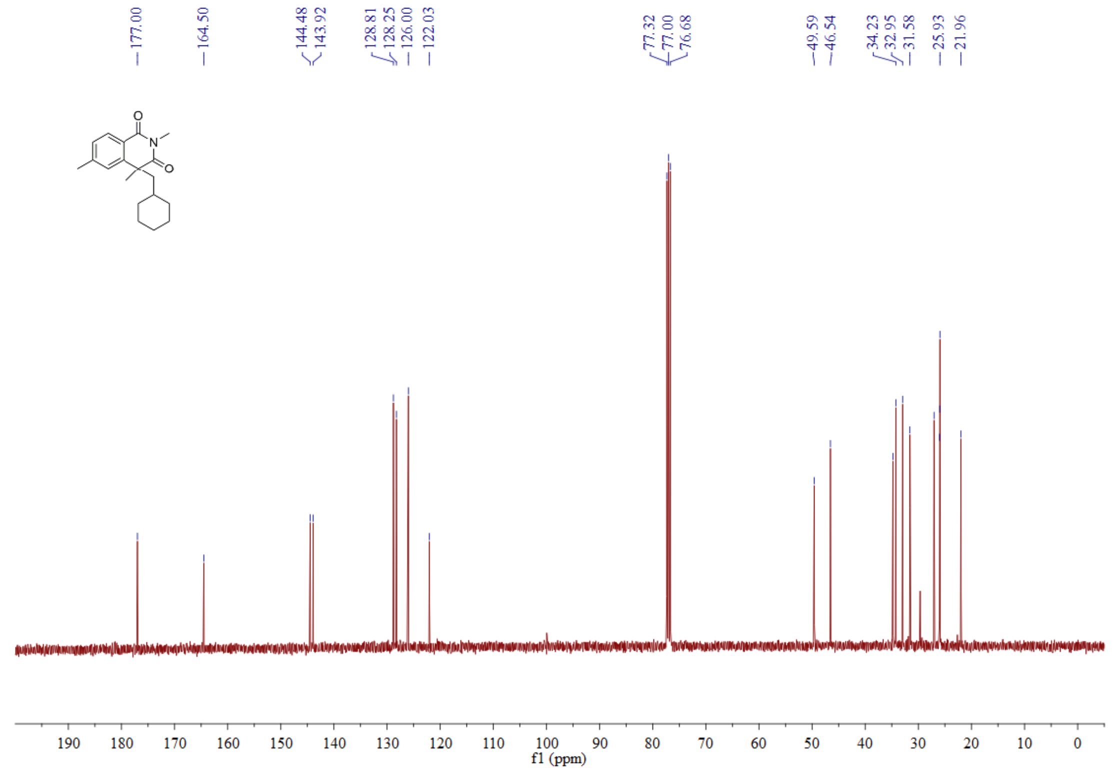

^{1}H NMR and ^{13}C NMR spectrum of **7ga**

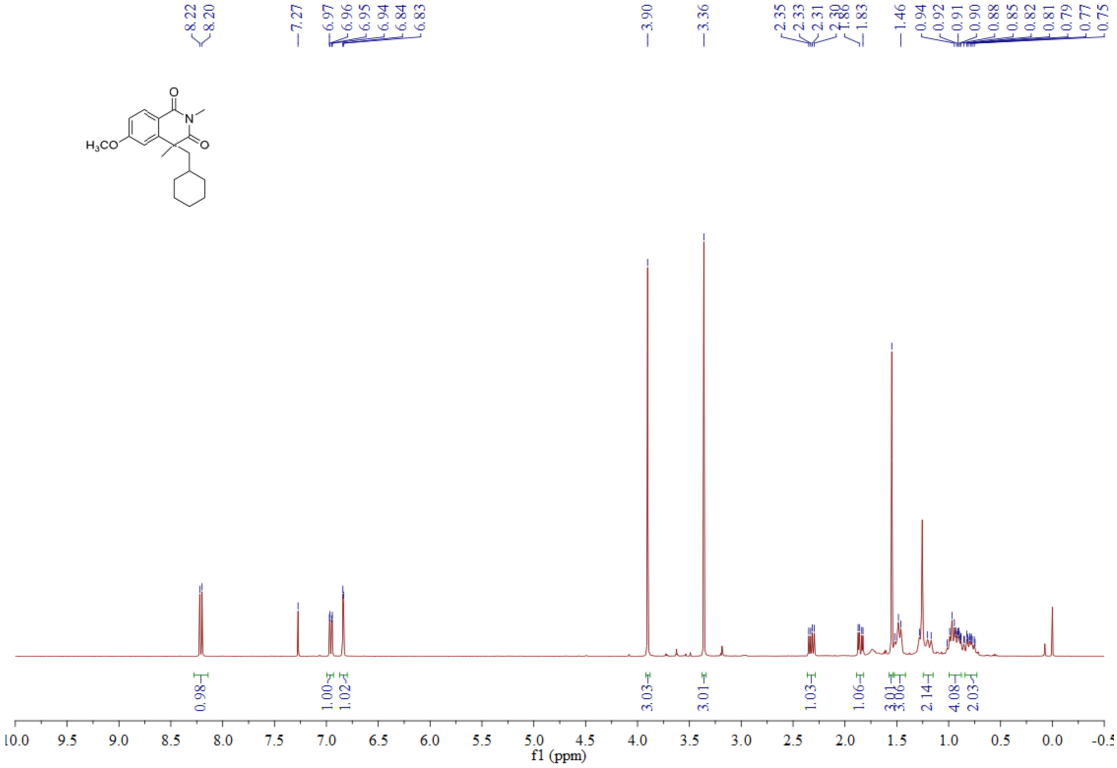

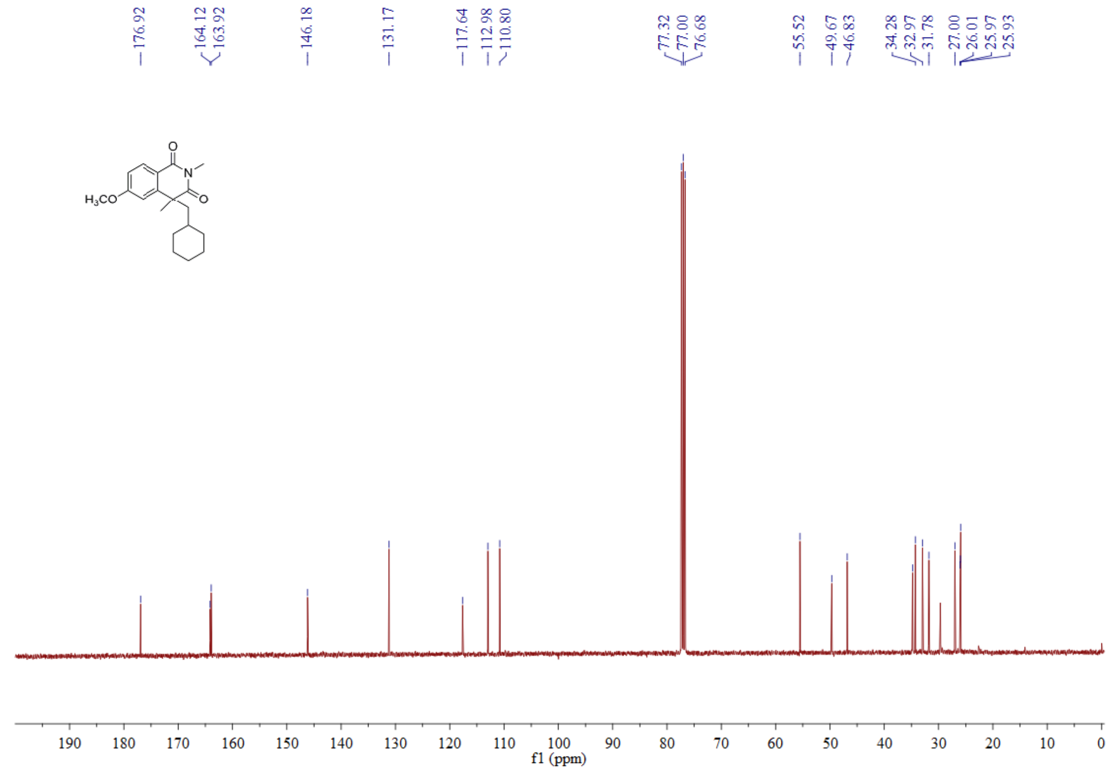

^{1}H NMR and ^{13}C NMR spectrum of **7ha**

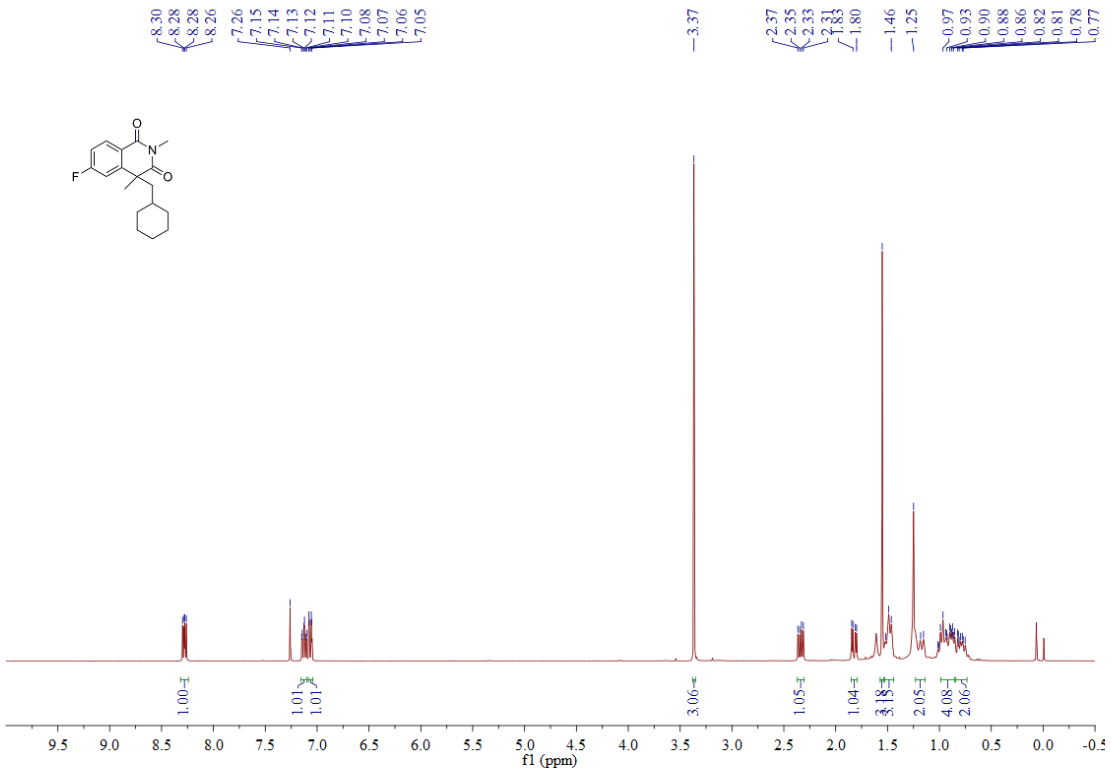

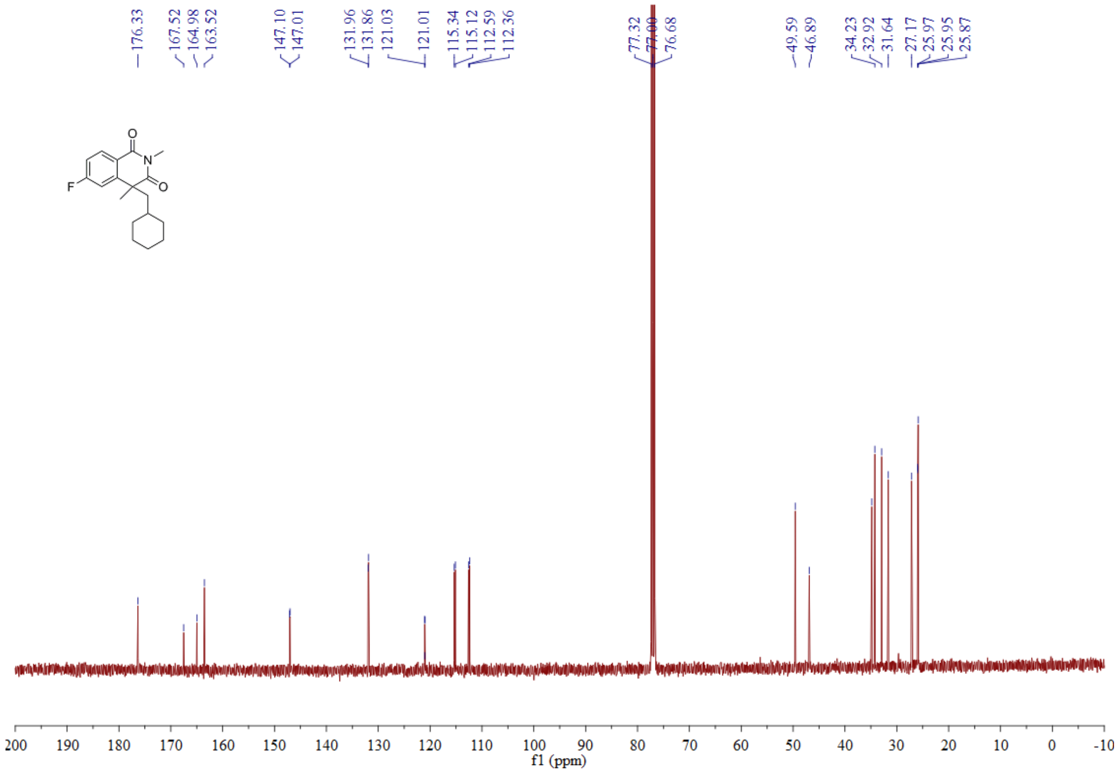

^{1}H NMR and ^{13}C NMR spectrum of **7ia**

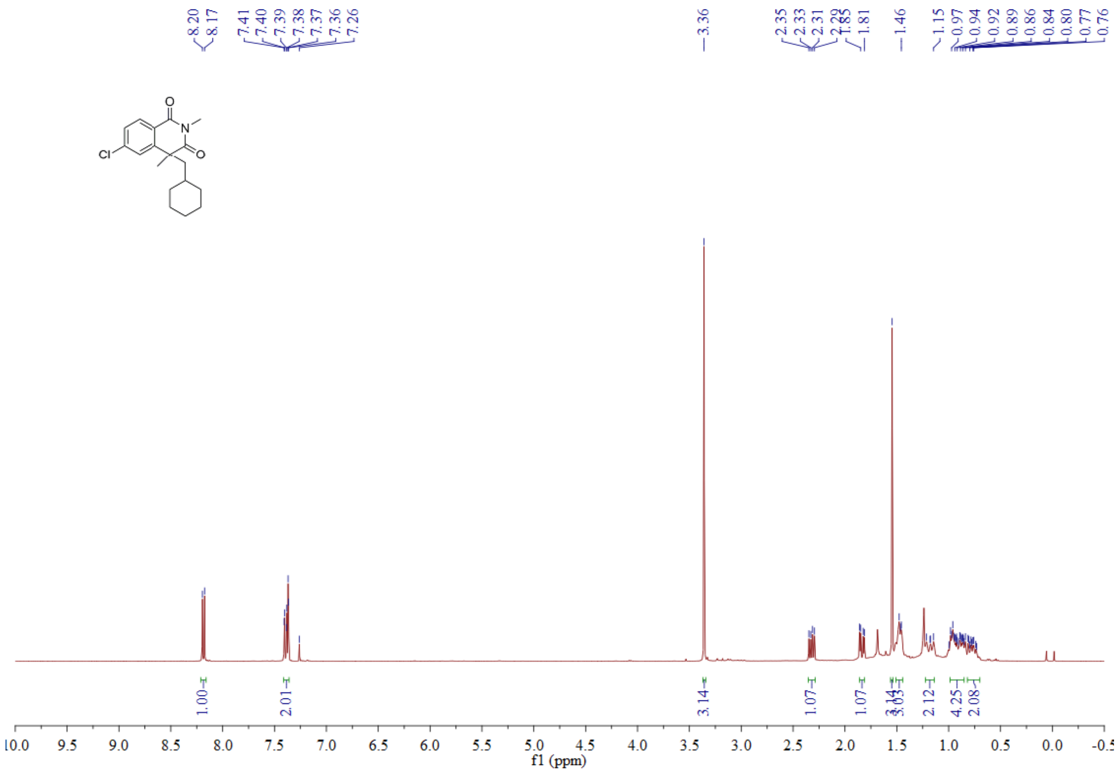

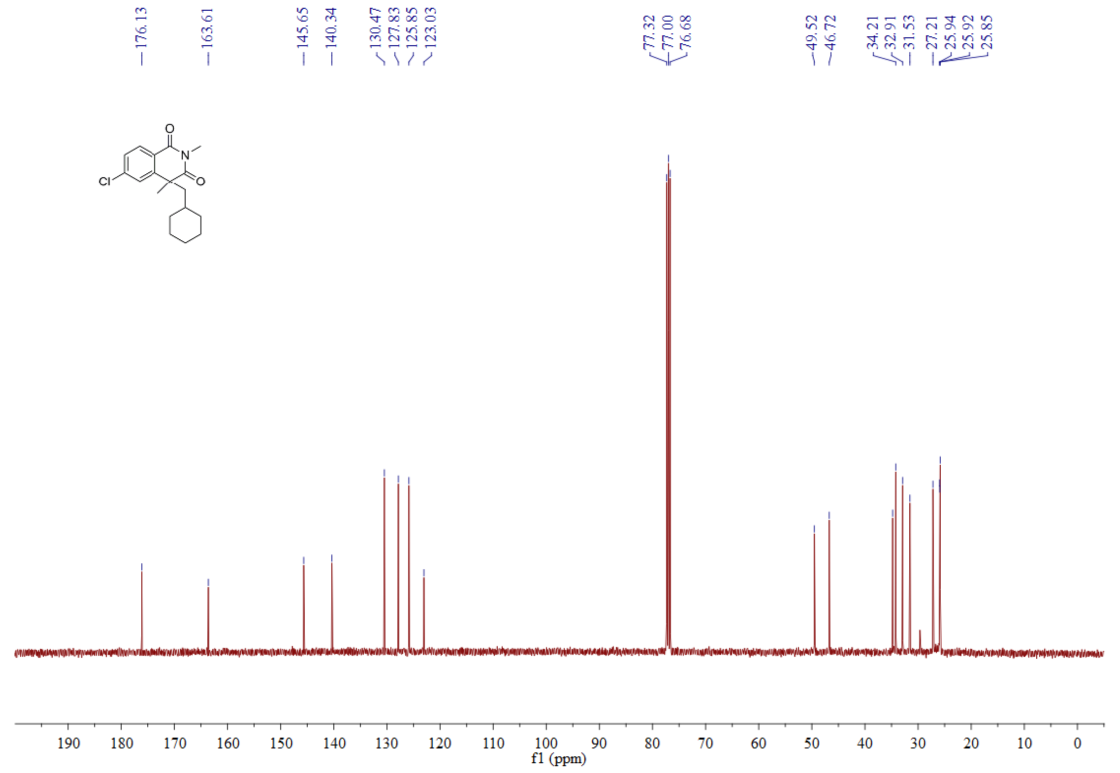

^{1}H NMR and ^{13}C NMR spectrum of **7ja**

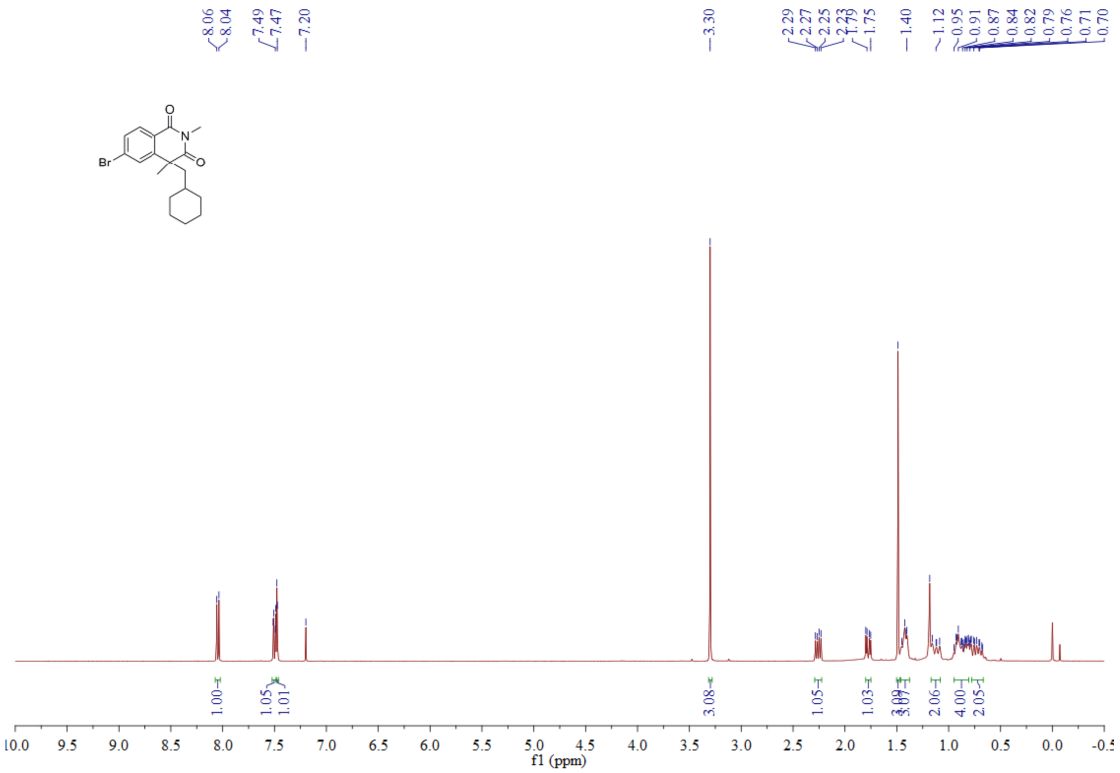

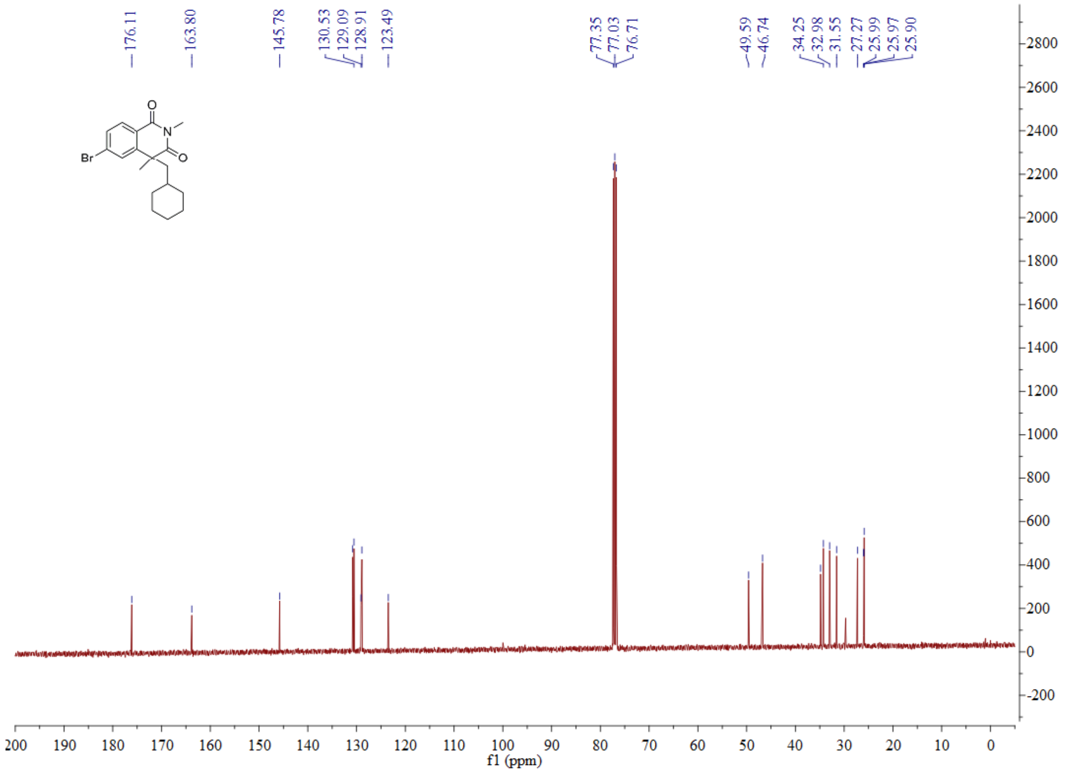

^{1}H NMR and ^{13}C NMR spectrum of **7ka**

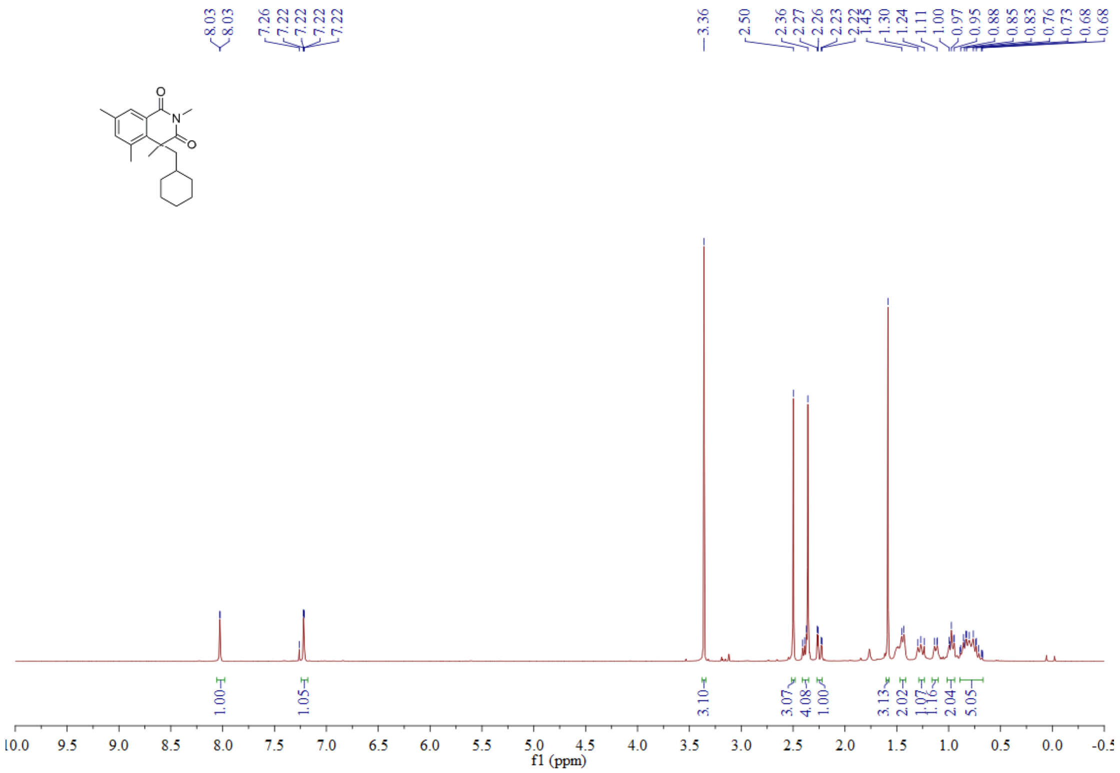

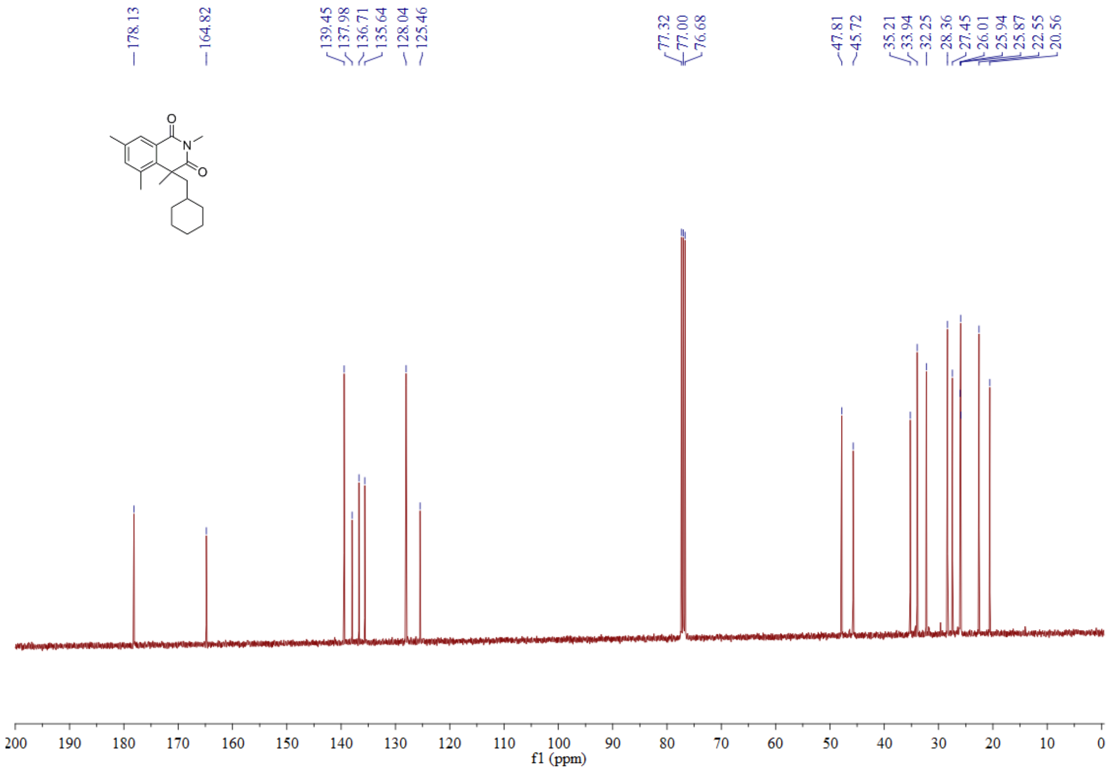

^{1}H NMR and ^{13}C NMR spectrum of **7la**

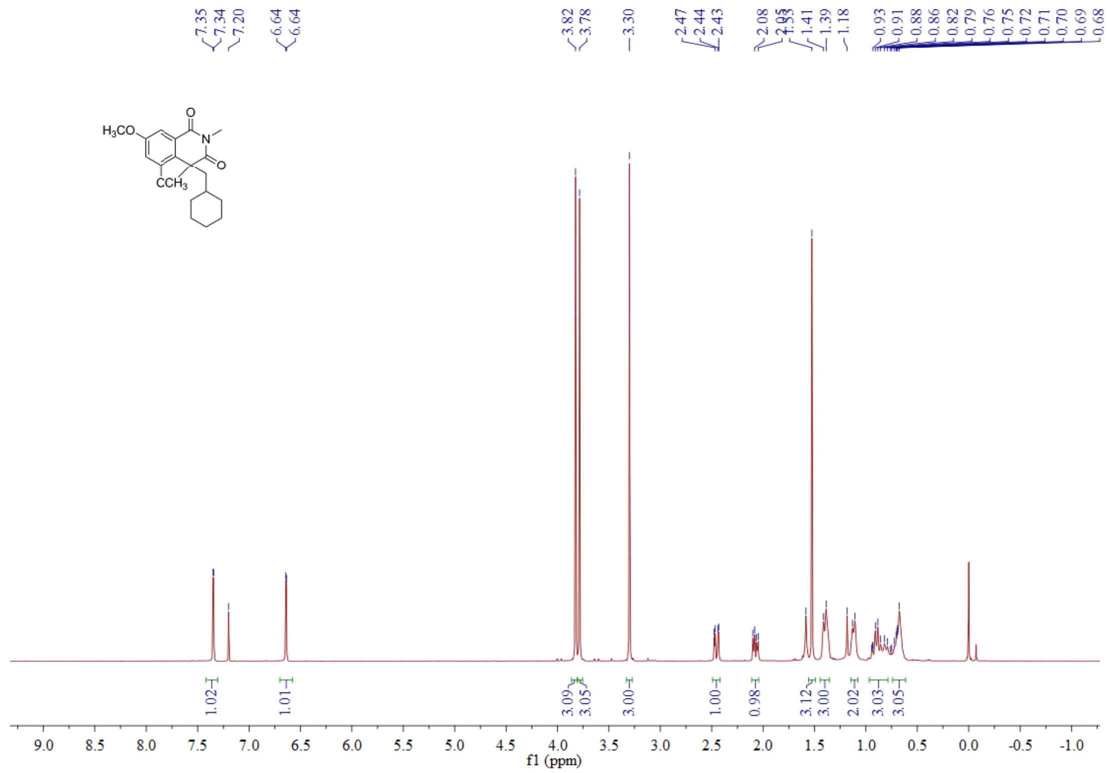

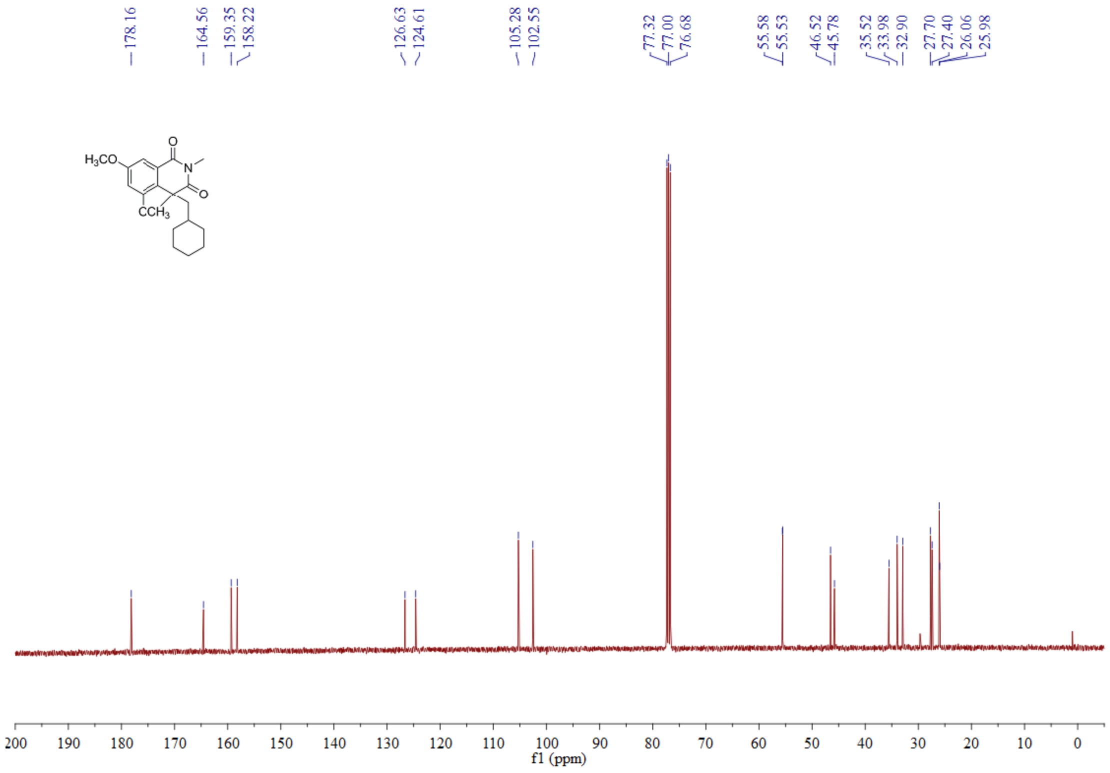

^{1}H NMR and ^{13}C NMR spectrum of **7ma**

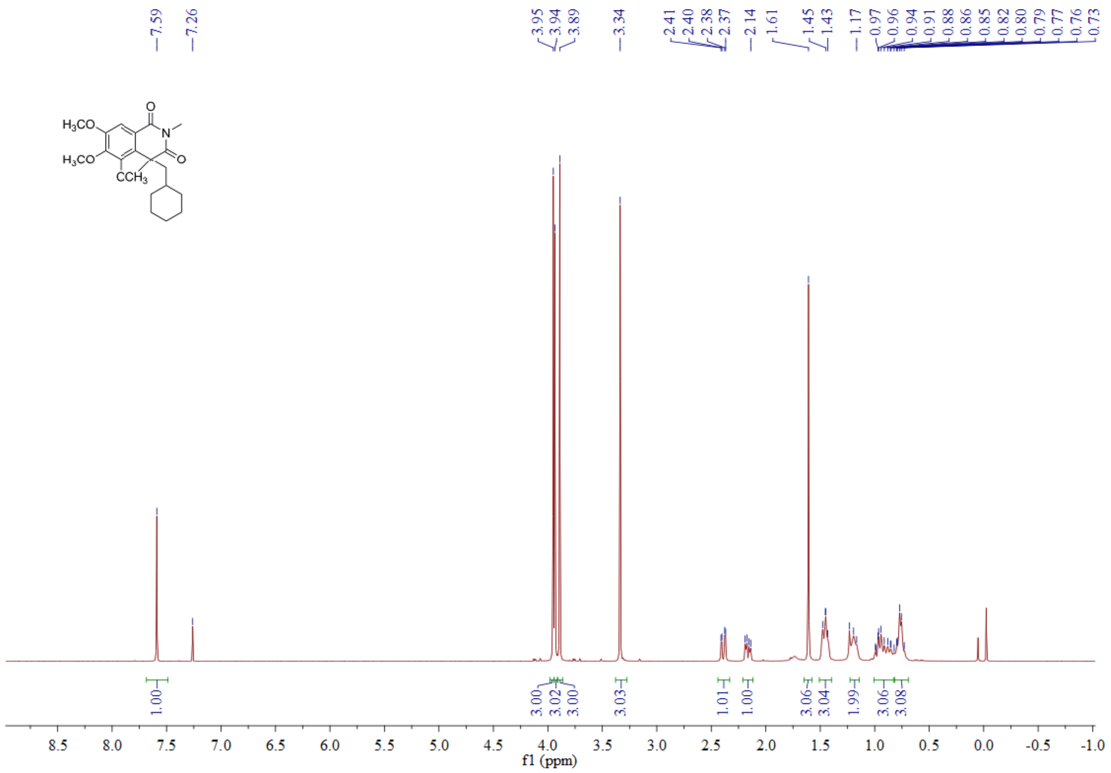

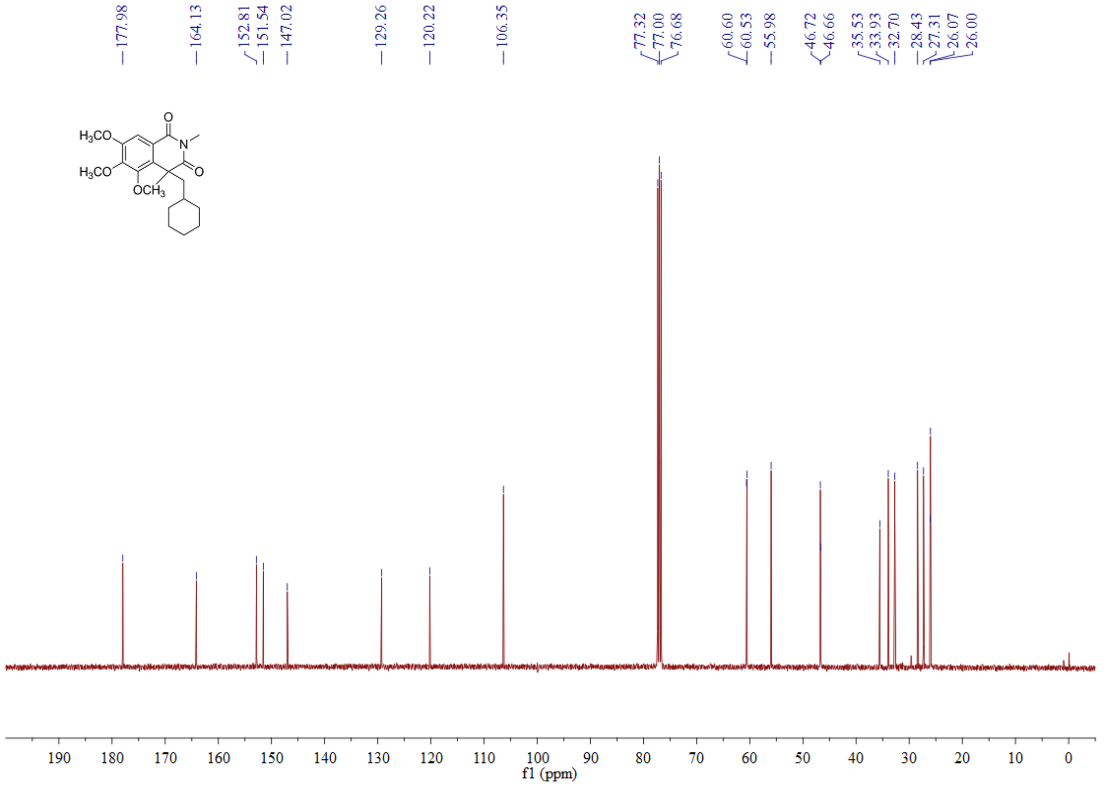

^{1}H NMR and ^{13}C NMR spectrum of **7na**

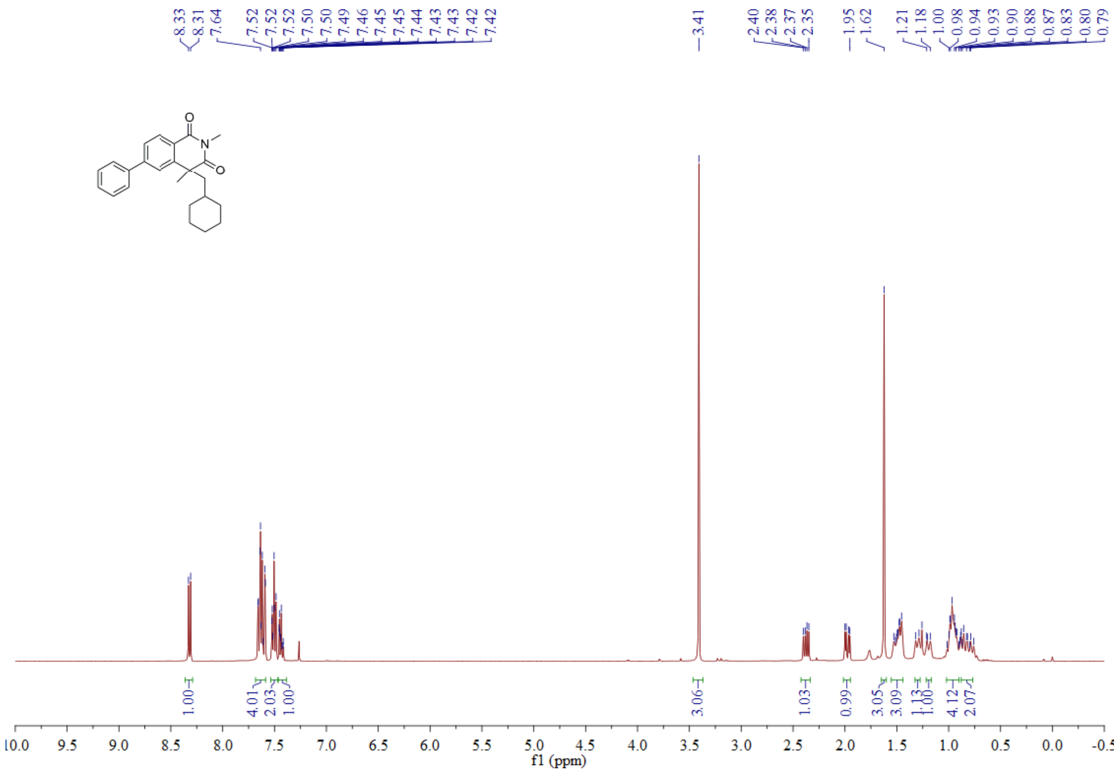

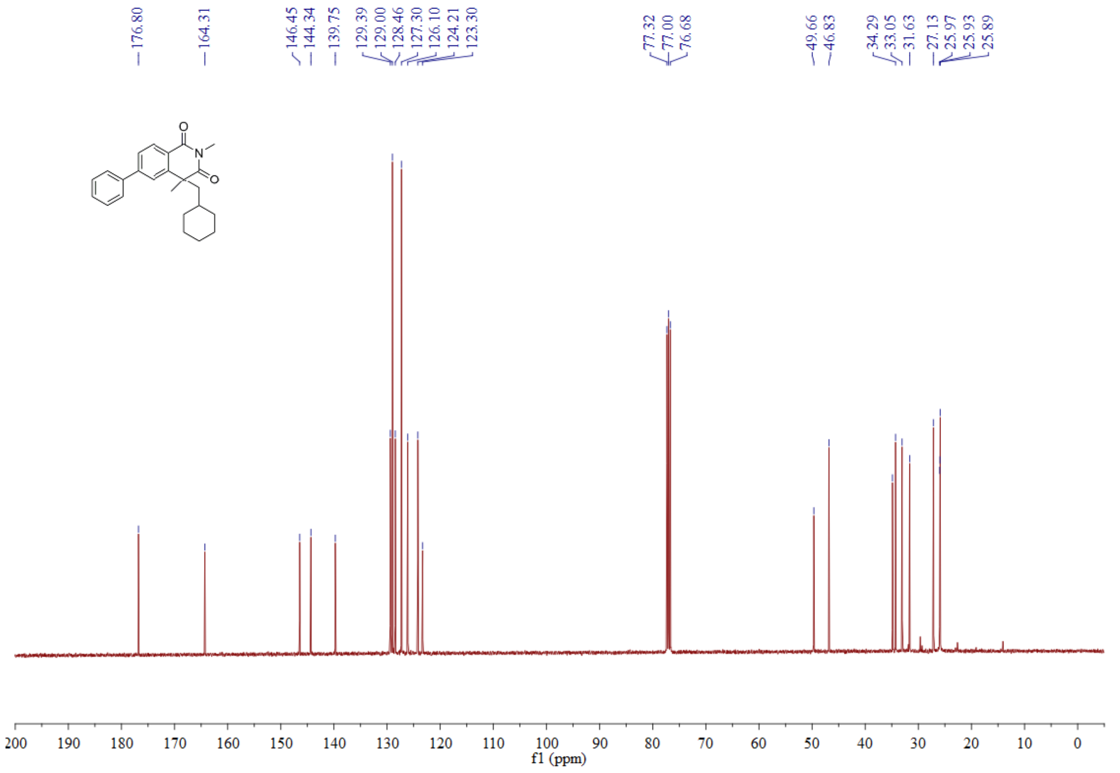

^{1}H NMR and ^{13}C NMR spectrum of **7oa**

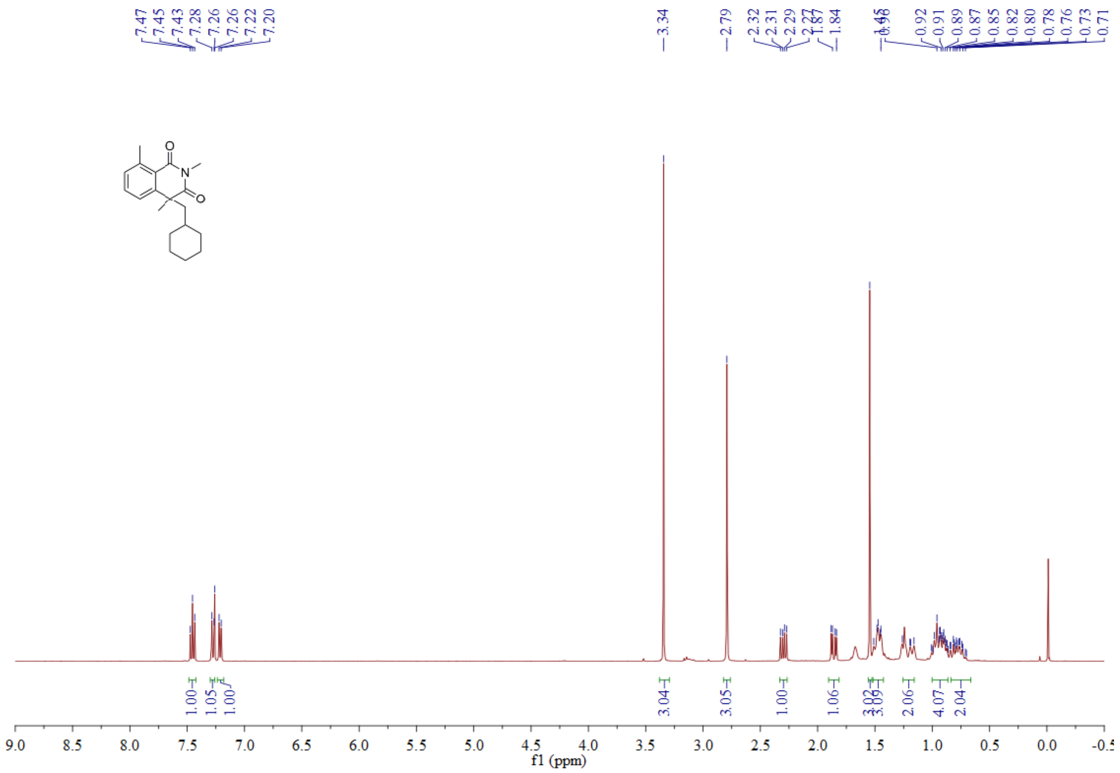

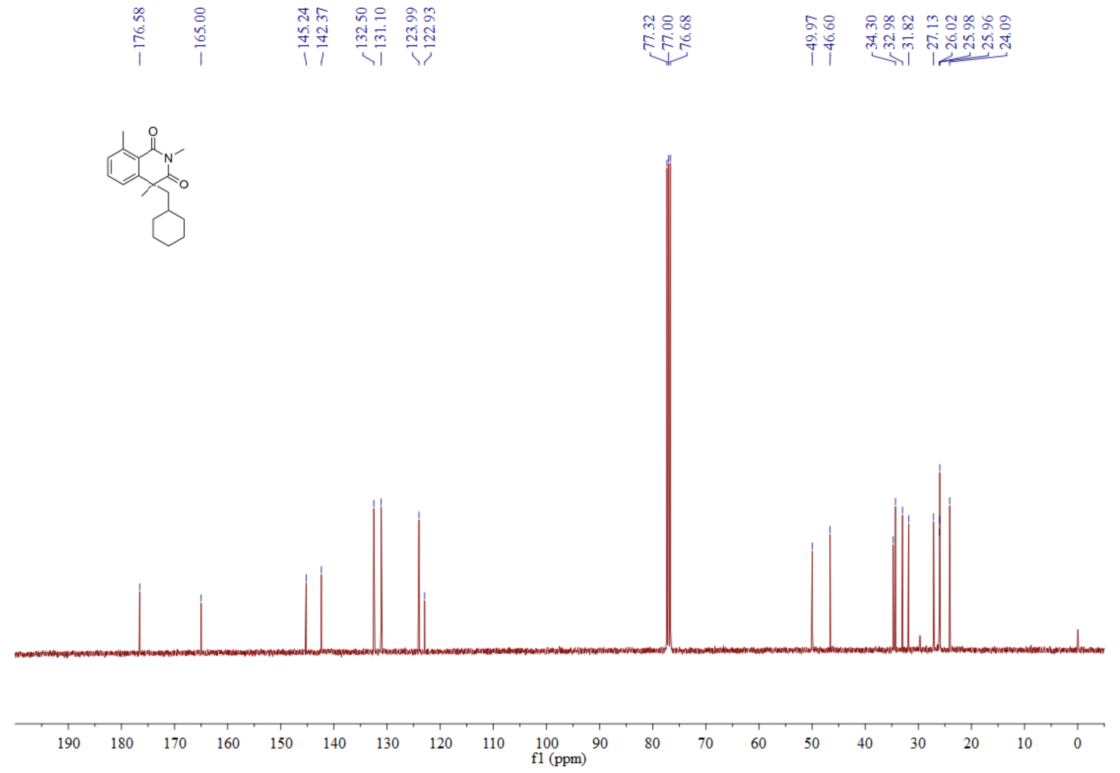

^{1}H NMR and ^{13}C NMR spectrum of **7pa**

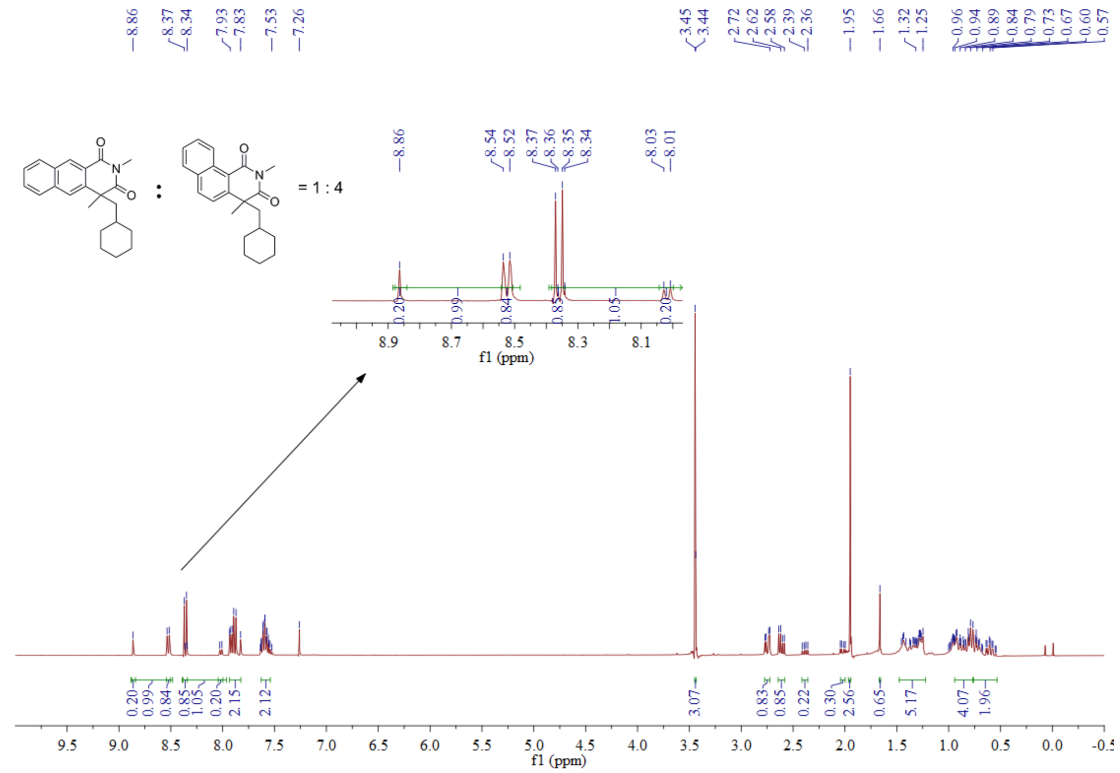

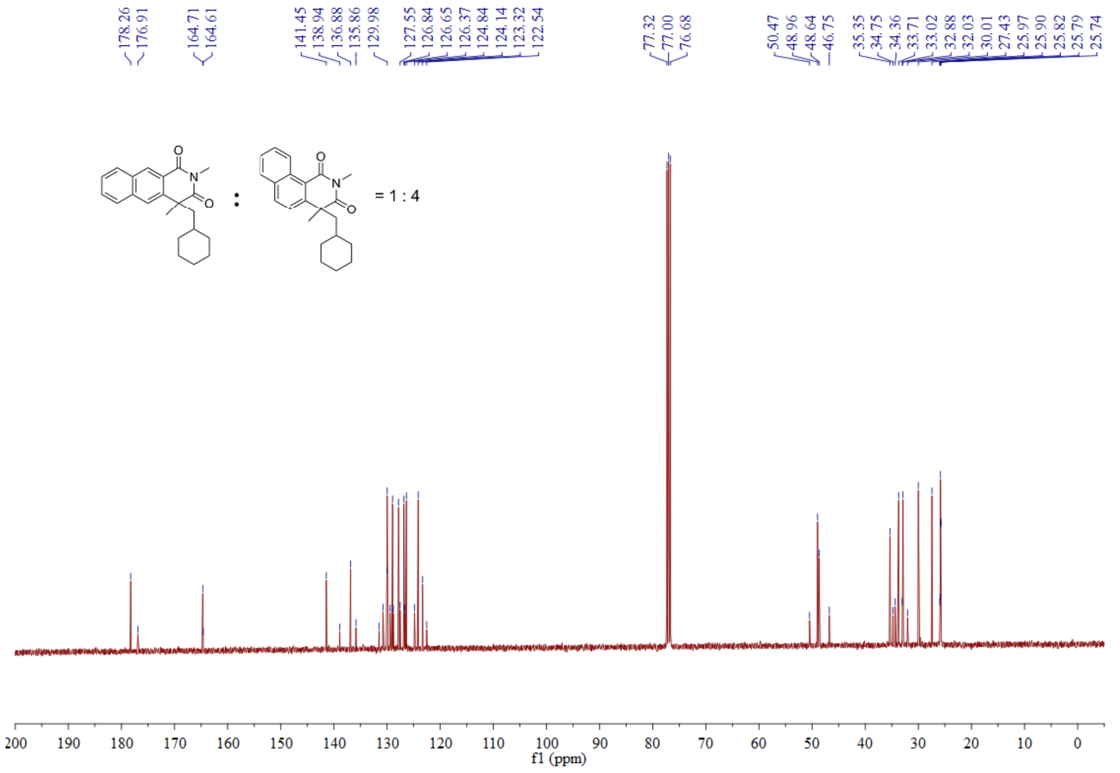

^{1}H NMR and ^{13}C NMR spectrum of **9aa’**

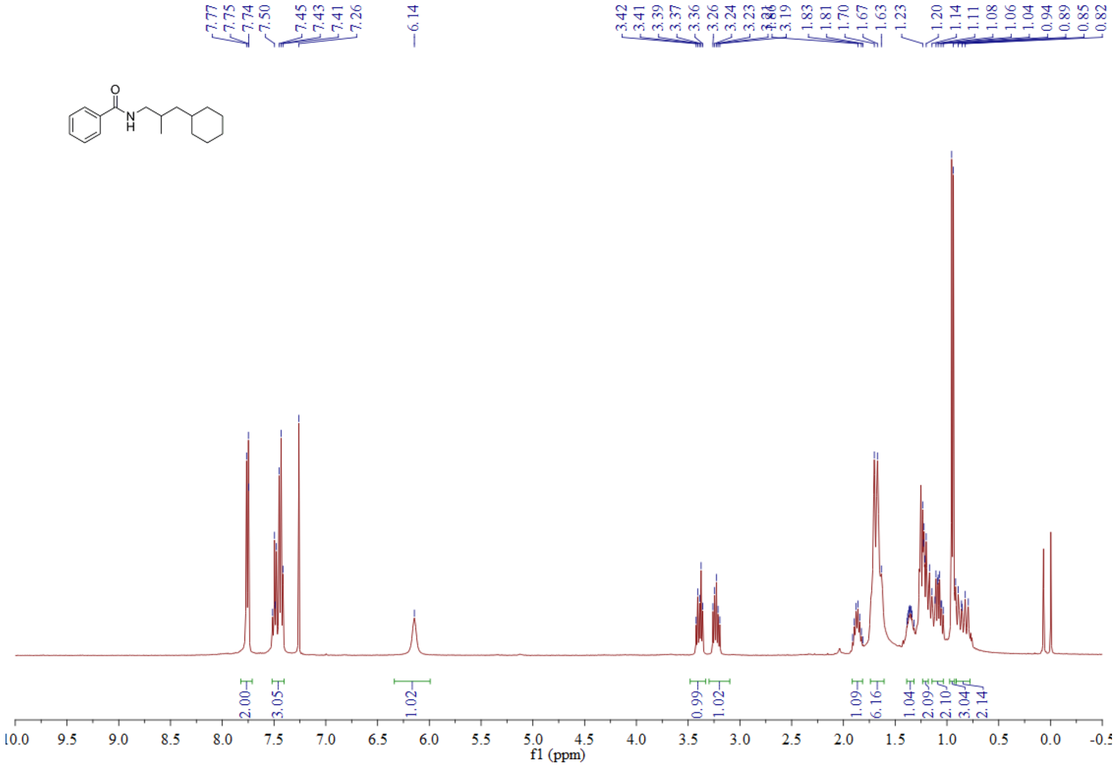

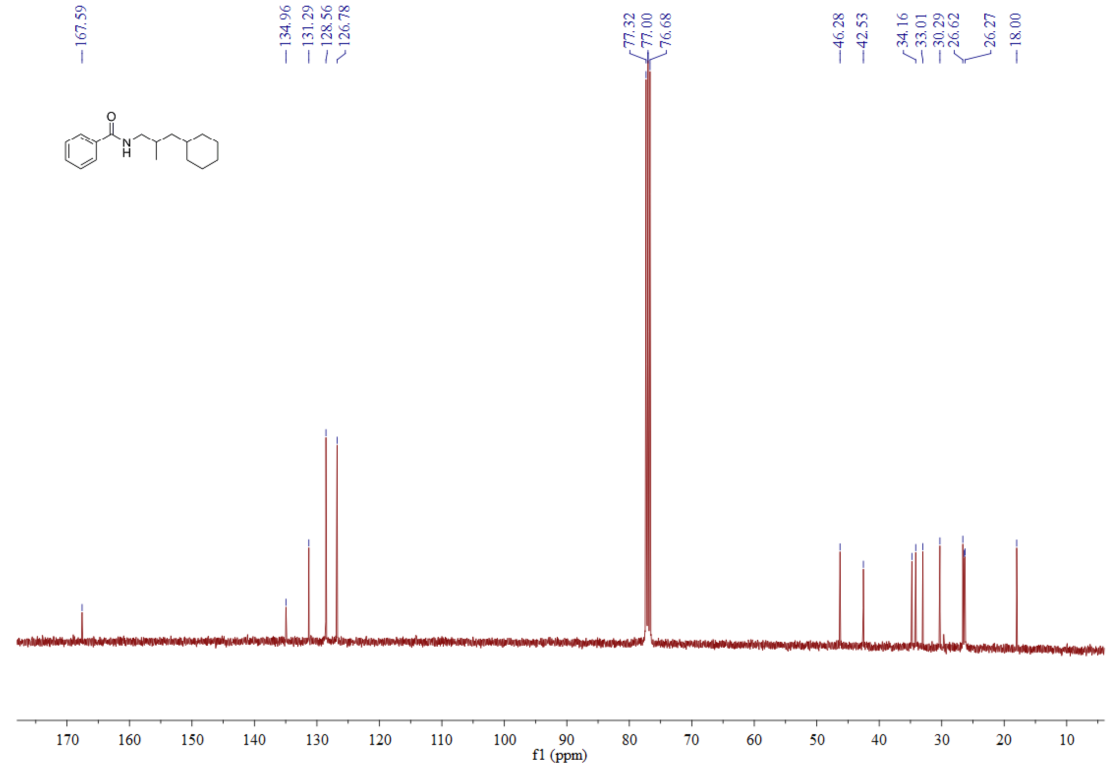

Supplement: File 1 — Experimental details and spectral data. [file Beilstein_J_Org_Chem-12-301-s001.pdf]
